# Curing the brain: in search for new astrocyte-specific therapies

**DOI:** 10.1038/s12276-026-01712-4

**Published:** 2026-04-24

**Authors:** Alexei Verkhratsky, C. Justin Lee, Heejung Chun, Christian Göritz, Tibor Harkany, Jae-Hun Lee, Sangkyu Lee, Maria Lindskog, Wuhyun Koh, Jan Mulder, Min-Ho Nam, Ole Petter Ottersen, Marcela Pekna, Milos Pekny, Aleksandra Pękowska, Hoon Ryu, Chang Ho Sohn, Evgenii O. Tretiakov, Verena Untiet, Tim J. Viney, Wongu Youn, Chenju Yi, Robert Zorec, Mijin Yun, Eunji Cheong, Agneta Nordberg

**Affiliations:** 1 Faculty of Biology, Medicine and Health, The University of Manchester, Manchester, UK; 2 International Joint Research Centre on Purinergic Signalling of Sichuan Province Chengdu University of Traditional Chinese Medicine, Chengdu, China; 3 Guangdong Provincial Key Laboratory of Digestive Cancer Research, The Seventh Affiliated Hospital of Sun Yat-sen University, Guangdong, China; 4 Celica, BIOMEDICAL, Ljubljana, Slovenia; 5 Department of Forensic Analytical Toxicology, School of Forensic Medicine, China Medical University, Shenyang, China; 6 Center for Memory and Glioscience, Institute for Basic Science, Daejeon, Republic of Korea; 7 Collage of Pharmacy, Yonsei-SL Institute, Yonsei University, Incheon, Republic of Korea; 8 Department of Cell and Molecular Biology, Karolinska Institutet, Stockholm, Sweden, Stockholm, Sweden; 9 Center for Neuromusculoskeletal Restorative Medicine, Hong Kong Science Park, Shatin, Hong Kong; 10 Department of Molecular Neurosciences, Center for Brain Research, Medical University of Vienna, Vienna, Austria; 11 Department of Neuroscience, Karolinska Institutet, Solna, Sweden; 12 Department of Medical Cell Biology, Uppsala University, Uppsala, Sweden; 13 Center for Brain Disorders, Brain Science Institute, Korea Institute of Science and Technology, Seoul, Republic of Korea; 14 Institute of Basic Medical Sciences, University of Oslo, Oslo, Norway; 15 Oslo New University College, Oslo, Norway; 16 Department of Clinical Neuroscience, Karolinska Institutet, Stockholm, Sweden; 17 Department of Clinical Neuroscience, Institute of Neuroscience and Physiology, Sahlgrenska Academy at the University of Gothenburg, Gothenburg, Sweden; 18 Dioscuri Centre for Chromatin Biology and Epigenomics, Nencki Institute of Experimental Biology, Polish Academy of Sciences, Warsaw, Poland; 19 Graduate School of Medical Science and Engineering, Korea Advanced Institute of Science and Technology, Daejeon, Republic of Korea; 20 Division of Astrocyte Driven Ionostasis, Center for Translational Neuromedicine, University of Copenhagen, Copenhagen, Denmark; 21 Department of Pharmacology, University of Oxford, Oxford, UK; 22 Department of Geriatrics, Seventh Affiliated Hospital of Sun Yat-sen University, Shenzhen, China; 23 Institute of Pathophysiology, Laboratory of Neuroendocrinology and Molecular Cell Physiology, University of Ljubljana, Ljubljana, Slovenia; 24 Department of Nuclear Medicine, Yonsei Univserity College of Medicine, Seoul, Republic of Korea; 25 Department of Biotechnology, College of Life Science and Biotechnology, Yonsei University, Seoul, Republic of Korea; 26 Department of Neurobiology, Care Sciences and Society, Center for Alzheimer Research, Karolinska institutet, Stockholm, Sweden; 27 Theme Inflammation and Aging, Karolinska University Hospital, Stockholm, Sweden

**Keywords:** Astrocyte, Mechanisms of disease

## Abstract

Astroglia, an extended class of homeostatic and defensive cells of the central nervous system (CNS), contribute to the pathogenesis of all known neurological and neuropsychiatric disorders. The pathophysiology of astrocytes is complex, mutable, disease and disease-stage specific. In neuroinflammatory lesions and in various chronic conditions, astrocytes undergo an evolutionary conserved defensive remodeling known as reactive astrogliosis, which produces highly heterogeneous reactive astrocytic phenotypes. Broadly, reactive astrogliosis can be classified into proliferative anysomorphic barrier-forming astrogliosis characteristic of traumatic CNS lesions and nonproliferative isomorphic gliosis widely manifested in chronic neuropathologies. In addition, in many pathologies, astrocytes undergo atrophy and asthenia with resulting loss of homeostatic support and neuroprotection precipitating neuronal damage. Reactive and atrophic astrocytes may coexist or emerge in sequence in a disease-stage-dependent manner. Several classes of astrocyte-specific molecules and processes implicated in various diseases of the CNS represent therapeutic targets. Astrocyte-specific therapeutic strategies may improve both disease-preventing and disease-modifying therapeutic outcomes.

## Understanding astrocyte roles in neurological disorders and treatments

Cognitive impairments are a major challenge in treating brain diseases. Many brain disorders, including Alzheimer’s and Parkinson’s, lack effective treatments. The brain consists of neurons and neuroglia, including astrocytes, which play crucial roles in maintaining brain health. Astrocytes help regulate neurotransmitters, protect neurons and maintain the blood–brain barrier. However, when astrocytes malfunction, they can contribute to diseases. For example, in Alzheimer’s disease, astrocytes become reactive and may worsen the condition by releasing harmful substances. Researchers are exploring ways to target astrocytes for treating cognitive disorders. This includes developing drugs that can modulate astrocyte activity or enhance their protective functions. By focusing on astrocytes, scientists hope to find new treatments for brain diseases that currently have no cure. In the future, understanding astrocyte functions could lead to breakthroughs in preventing and treating cognitive impairments.

This summary was initially drafted using artificial intelligence, then revised and fact-checked by the author.

## From neuronocentrism to the inclusive brain—the key for therapeutic success

Cognitive impairments are caused by many pathologies affecting the ability to think, concentrate, remember or make decisions. Diseases of the brain, which lead to cognitive and neurological deficiencies and limit the quality of life in the aging global population, represent the main therapeutic challenge of the twenty-first century. There are no effective therapeutic strategies for most of the major disorders of the brain, including ischemic, toxic, autoimmune, neurodevelopmental, neuropsychiatric, malignant and neurodegenerative pathologies; for many of these, neither disease-preventing nor disease-modifying medicines exist. This reflects the complexity of the human nervous system forged by ~500 million years of evolution, which assembled 200 billion of highly heterogeneous cells into intricate networks capable of lifelong structural and functional plasticity. The brain cells include executive neurons capable of long-range fast signaling connecting the brain to the body, the homeostatic and defensive neuroglia, and cells of the brain vasculature. The segregation of functions between neurons and neuroglia emerged early in evolution^1^. Invertebrates possess many types of neuroglial cells that create brain–body barriers, protect and support axons, and produce complex defensive responses to external insults. In vertebrates and mammals, neuroglia underwent further advancement, becoming the main element responsible for nervous tissue homeostasis and protection.

The human central nervous system (CNS) contains three main types of neuroglia: the homeostatic and neuroprotective astroglia, the myelinating oligodendroglia and the defensive microglia^2–7^. All cells in the brain are linked by multiple feedforward and feedback connections, which maintain the function, versatility and plasticity of the neural tissue^8^. In response to pathological insults, neuroglial cells actively change to protect the nervous tissue; at the same time, loss of function of neuroglia compromises the resilience of the brain and causes neuronal damage^9^. The last century witnessed seminal discoveries of ion channels that explained neuronal excitability, information processing, and plasticity, leading to the predominance of neuron-centric views in neurology, psychiatry and neuropharmacology. Consequently, drugs solely targeting neuronal pathways were conceived and developed, whereas neuroglia remained overlooked. This represents a therapeutic gap, which needs to be addressed. In this Review, we focus on astroglia; we briefly introduce these cells from an evolutionary and functional perspective, we present the general pathophysiology of astroglia, and finally we discuss how to target astroglial cells in cognitive brain disorders.

## Astrocytes—guardians and housekeepers of the brain

**Fig. 1:**
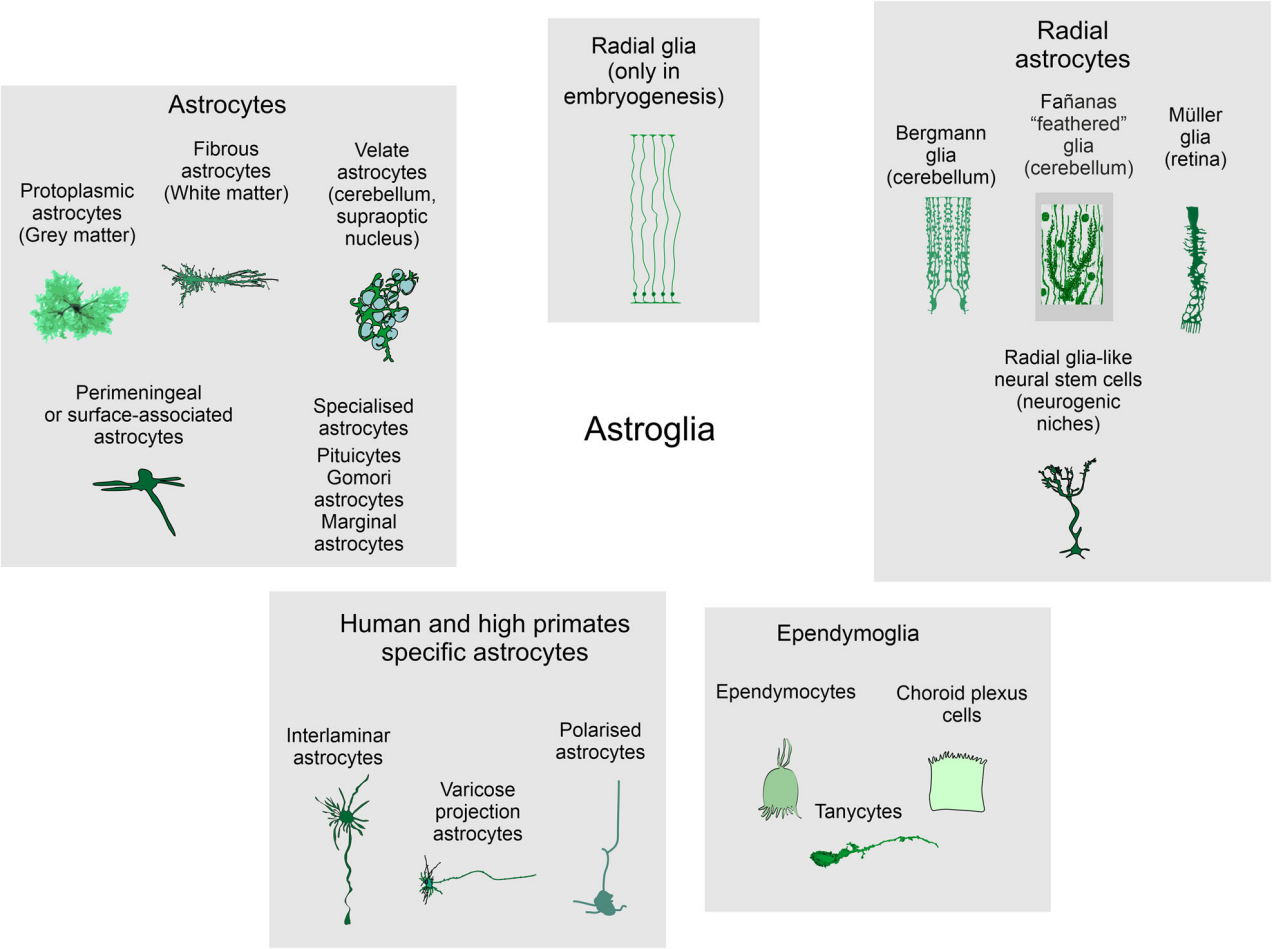
Classification of astroglia. The plates show diff feremt types of astroglial cells with typical morphology.

**Fig. 2:**
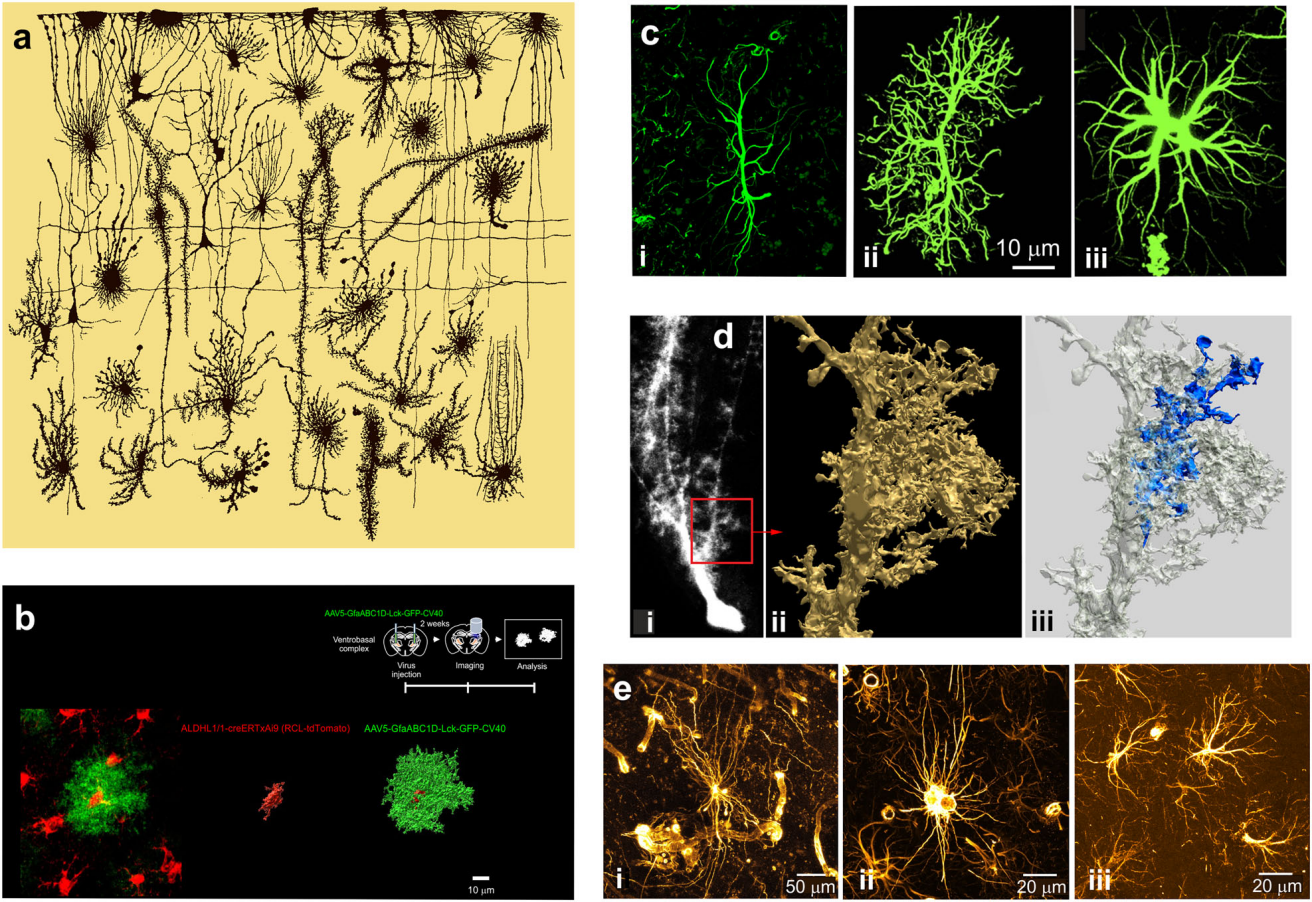
Morphological heterogeneity of astroglia. **A** Diversity of astrocyte shapes in the human fetal cortex revealed by Golgi staining. Reproduced from ref. ^673^. **B** Morphology of thalamic astrocytes labeled with membrane-targeted GFP. Astrocytes were transduced with AAV-GfaABC1D-Lck-GFP, which drives astrocyte-specific expression under the minimal GfaABC1D promoter, enabling clear visualization of fine membrane processes and leaflet structures. Image is courtesy of Prof. Eunji Cheong, Yonsei University, Republic of Korea. **C** Rodent astrocytes from different brain regions (i, entorhinal cortex; ii, prefrontal cortex; iii, CA1 area of hippocampus) immunolabeled with antibodies against GFAP, which visualizes soma and major branches. Reproduced, with permission, from ref. ^14^. **D** Morphology of the cerebellar Bergmann glial cell. (i) Fluorescence light micrograph of a dye-injected Bergmann glial cell is shown; the red square (20 × 20 mm) corresponds to the portion that was reconstructed from consecutive ultrathin sections. (ii) One of the lateral appendages, arising directly from fiber with all the other side branches omitted for clarity. (iii) The same appendage as shown in (ii), but with one of the appendages marked by blue. Reproduced, with permission from ref. ^674^. **E** Astrocytes from human and mouse brains. (i) Astrocyte in the human anteroventral thalamic nucleus with long processes contacting blood vessels. (ii) Astrocyte in the human presubiculum. (iii) Astrocytes in the mouse subicular complex. All images show vimentin immunoreactivity (maximum intensity *z*-projections: (**i**) 20 μm thick; (ii) and (iii) 17.4 μm thick). Note the difference in size between human and mouse astrocytes. Images courtesy of Prof. Tim Viney, University of Oxford, UK.

The concept of neuroglia as a connective tissue of the CNS was introduced by Rudolf Virchow^10,11^. The introduction of the Golgi black staining technique^12^ revealed the diversity and heterogeneity of neuroglia populating the brain and spinal cord. Many of these cells, when stained with the Golgi technique, have a star-like appearance, which prompted Michal von Lenhossék to coin the name astrocyte^13^. Astrocytes belong to a larger class of astroglia (Fig. 1), which includes various types of parenchymal astrocytes (protoplasmic, fibrous, velate, marginal and so on), radial astrocytes (Bergmann glia of the cerebellum, Műller glia of the retina, and radial stem astrocytes of the neurogenic niches) and ependymoglia (ependymocytes, tanycytes and choroid plexus cells). The shapes of astroglia are diverse and vary across brain regions^14^. Protoplasmic astrocytes have a highly complex spongioform (not star-like, which is an artifact of staining with the Golgi technique or immunolabeling with cytoskeletal antibodies) shape defined by a mass of peripheral processes known as leaflets^15,16^; long processes of fibrous astrocytes align with axons in the white matter and contact nodes of Ranvier^17^, whereas Müller glia resemble pillars that span the whole thickness of the retina and integrate retinal neurons into independent functional units and acting as light guides^18,19^. Although morphologically heterogeneous (Fig. 2), the common function of astroglia is the preservation of the brain homeostasis, neuroprotection and brain defense. The core functions of astrocytes include controlling the homeostasis of neurotransmitters (through uptake, catabolism and the supply of precursors), K^+^ buffering, metabolic support, scavenging of reactive oxygen species (ROS), regulation of water transport and interstitial fluid, formation—through perivascular endfeet—of the parenchymal component of the blood–brain barrier (glia limitans perivascularis), contribution to chemosensing, regulation of energy homeostasis, participation in transmitophagy, and many other processes^14^. For that, astrocytes are equipped with many receptors (to sense the environment) and numerous transporters that are the backbone for astrocytic homeostatic function; many of these transporters are Na^+^-dependent, and hence, the astrocytic α2-containing Na^+^–K^+^ pump is central for astrocytes homeostatic capacity^20^. The complement of receptors and transporters varies substantially between brain regions and is regulated by the immediate neurochemical environment.

Parenchymal astrocytes (protoplasmic, fibrous, Bergmann glia, Müller glia and so on) establish intricate contacts with synapses, creating a multipartite (and multicellular) synaptic complex—also known as the synaptic cradle—that oversees synaptogenesis, synaptic maturation, maintenance and extinction^21–24^. Astrocytes support and regulate synaptic transmission through many astrocyte-specific mechanisms^25^. Astrocytic glutamate transporters, for example, define the kinetics of glutamate in the synaptic cleft, while astrocytic glutamine is obligatory for neuronal production of glutamate and GABA^26,27^. Astrocytes are intimately interconnected with all cells of the nervous tissue (the brain active milieu^15^), with numerous feedback and feedforward signals contributing to the integration of a broad variety of cellular functions in the CNS.

## The evolutionary advancement of astroglia is a defining feature of the human brain

The evolutionary history of neuroglia begins with the advent of bilateralia; the existence of neural supportive cells in cnidarians and ctenophores remains debatable^28–30^. The first neuroglial cells were associated with sensory organs known as sensilla in roundworms and flatworms; notably, these glia–neuron sensory units are evolutionarily conserved from *Caenorhabditis elegans* to humans^31^. Parenchymal glia emerged in some Platyhelminthes; in particular, glial cells associated with the neuropil and synapses populate the brains of planarians. Neuroglia are present in all Ecdysozoa and Lophotrochozoa, are well developed in Annelida, and are even more developed and highly diverse in Arthropoda, particularly in insects and crustaceans. In *Drosophila*, for example, up to 36 distinct glial cell types were distinguished; some of these cells create brain–body barriers (perineurial and subperineurial surface glial cells), others cover neuronal somata (cortex glia) and some ensheath synapses in neuropil (astroglia-like cells)^32,33^. In Echinodermata (which share the common ancestor with Chordata), the radial glia emerged, which signals the appearance of the layered cytoarchitecture. Increase in the brain thickness took place in parallel with the emergence and diversification of parenchymal astrocytes, which become larger and more complex in the brain of *Homo sapiens*.

**Fig. 3:**
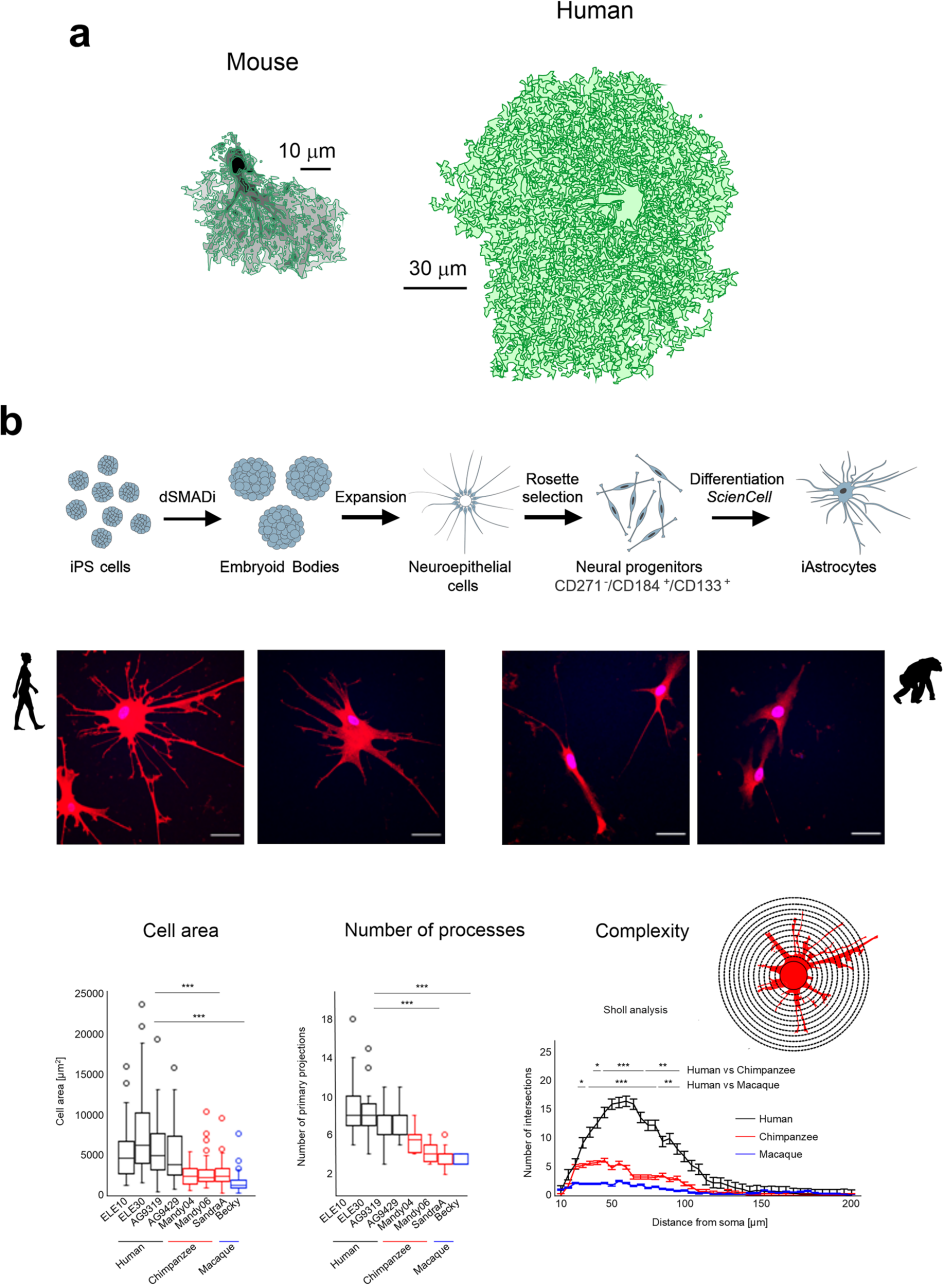
Evolutionary advance of astrocytes. **a** 3D reconstructions of protoplasmic astrocytes filled with fluorescent dye Alexa 594 from adult mouse and human. Modified and reproduced from refs. ^134,135^. **b** Primate iAstrocytes recapitulate evolutionary features of fetal brain astrocytes. Top: experimental strategy to obtain primate (human, chimpanzee and macaque) iAstrocytes. Middle: human iAstrocytes feature more complex morphology than higher primates. Representative examples of CD44-immunostained iAstrocytes from Human (left two images) and chimpanzee (right two images). Scale bar, 50 μm. Bottom right: total cell area of iAstrocytes derived from distinct donors (ELE10, *n* = 51; ELE30, *n* = 50; AG93, *n* = 62; AG94, *n* = 50; SandraA, *n* = 49; Mandy04, *n* = 49; Mandy06, *n* = 51; Becky, *n* = 47. *P*: two-sided *t*-test; ***P < 0.001). Bottom middle: number of primary projections in iAstrocytes per line. Only cells featuring clear-cut projections were considered (ELE10, *n* = 35; ELE30, *n* = 26; AG93, *n* = 17; AG94, *n* = 19; SandraA, *n* = 13; Mandy04, *n* = 18; Mandy06, *n* = 22; Becky, *n* = 13. *P*: two-sided *t*-test; ***P < 0.001. Bottom: Sholl analysis of iAstrocytes. Intersections were measured every 5 μm in 200-μm radius from the cell soma (concentric circles; ncells 20 (human), 14 (chimpanzee); 8 (macaque); *P*: two sample Kolmogorov-Smirnov test: Hs versus Pt *P* = 0.008; Hs versus Mm *P* = 0.00004; Individual intersections pairs: *t*-test (***P < 0.0001, **P < 0.001, * **P < 0.01). Modified and reproduced from ref. ^45^.

Cell morphology is deeply entwined with function, and interspecies comparisons of cell shape may instruct on broad trends in evolution. Likewise, while many evolutionary changes may be related to the generation of new genes, it is broadly recognized that the bulk of interspecies differences arise corollary to changes in gene activity. Moreover, brain evolution is intimately linked to neurological disorders, many of which are specific to humans^34,35^. Comparative studies of morphology demonstrated an explosion of astrocytes of various sizes, specific shapes (interlaminar astrocytes) and complexity in the human brain^36–40^ (Fig. 3). Single-cell RNA sequencing (RNA-seq) further shows that, across the large evolutionary distances separating humans from monkeys and rodents, astrocytes, like other neuroglial cells, exhibit greater transcriptomic changes than neuronal cells^41–44^. Hence, changes in the astrocyte lineage are instrumental for brain evolution.

Hundreds of differentially expressed genes (DEGs, both protein-coding and noncoding) between human and nonhuman primate samples were identified by RNA-seq of stem cell-derived astrocytes (iAstrocytes)^45^. In particular, the upregulation of the Hippo-pathway-regulated transcription factor TEAD3 in the human lineage contributes to the increased size and complexity of human astrocytes. Moreover, human astrocytes showed upregulation of genes implicated in the formation of extracellular vesicles (EVs), and indeed human iAstrocytes produce more EVs than chimpanzee and macaque iAstrocytes. Remarkably, treating macaque iAstrocytes with human iAstrocyte-derived EVs leads to increased size and complexity of the macaque cells, highlighting a previously overlooked role of EVs—and, evidently, the cargo they carry—in brain evolution^45^. Altogether, numerous genes are activated in astrocyte evolution translating into the gain of human-specific features of these cells. Some of these genes can be linked to human-specific neurological disorders, and understanding how they change in evolution can help to explain the natural history of these diseases.

## Diversity of astrocytes—cellular, genetic and molecular features

The remarkable morphological diversity of astroglia has been noted from the very early studies; the invention of Golgi ‘*reazione nera*’^12,46^ allowed visualization of many morphotypes of radial (such as Bergmann glia or interlaminar astrocytes) and parenchymal astrocytes (protoplasmic, fibrous, velate and others; Figs. 1 and 2). Whether this morphological diversity translates into molecular heterogeneity and, consequently, functional specialization remained less clear. For a long time, astrocytes were considered a rather homogeneous cell population across the entire brain.

As discussed earlier, the archetypal role of astrocytes is to support and protect nervous tissue. In doing so, astrocytes dynamically modulate their interactions with neurons and synapses, with other glial cells, and with the vascular network that supplies nutrients to neurons, synapses and glia, while also regulating local blood flow^15,47,48^. To serve these manifold functions, astrocytes retain remarkable, life-long cellular plasticity. Moreover, subsets of astrocytes recruited to neuronal circuits under focal demand can episodically undergo cell-state changes, reflected in bursts of transcriptional activity that rapidly re-express gene sets—whether encoding cytoskeletal proteins, transmembrane transporters or antioxidant enzymes—tailored to their microenvironmental stimuli^49–51^. For this very reason, the molecular classification of astrocytes, and the definition of the spatial segregation of their subtypes, if any, are not only more complex than previously thought, but also paramount to dissect the diversity and specificity of mechanisms underlying their cellular plasticity.

Even though the morphological classification of astrocytes is well developed (Fig. 1), these cells have long been—and still often are—functionally classified by default as either ‘resting-state’ or ‘activated’ in response to a stimulus, whether toxic or benign. These definitions are confusing, because astrocytes never ‘rest’ in the healthy brain, while their shape and function are tightly linked and interdependent. Instead, they dynamically respond to physiological stimuli and mount multiple signaling, homeostatic, morphological and secretory responses^14,52–54^. The classification of healthy astrocytes based on their molecular signatures, together with their specific locations, has only begun to evolve—particularly through lineage-tracing studies of radial glia domains, whose neuronal and astrocyte progeny express remarkably similar homeobox gene sets, migrate along identical routes and form microcircuits^55–57^. Despite these remarkable observations, an ensuing avalanche of single-cell RNA-seq studies first molecularly separated astrocytes as a quasi-homogeneous cell group from neurons, oligodendroglia, microglia and vascular cell lineages. More recently, the subgrouping of astrocytes through single-most important molecular features in physiological states or upon genetic manipulations was accomplished^58–60^. Nevertheless, ambiguities remain regarding the bona fide subclasses of astrocytes and their spatial distribution in the nervous system under physiological conditions. This is largely due to the difficulty of distinguishing ‘cell identity feature sets’ from ‘cell-state markers’ in transcriptomic data, with classical correlation analyses relying heavily on statistical differences to segregate cell identities. For astrocytes, however, the most pronounced changes in gene expression are typically associated with their engagement in specific tasks, rather than with their developmental trajectories or spatially defined identities.

**Fig. 4:**
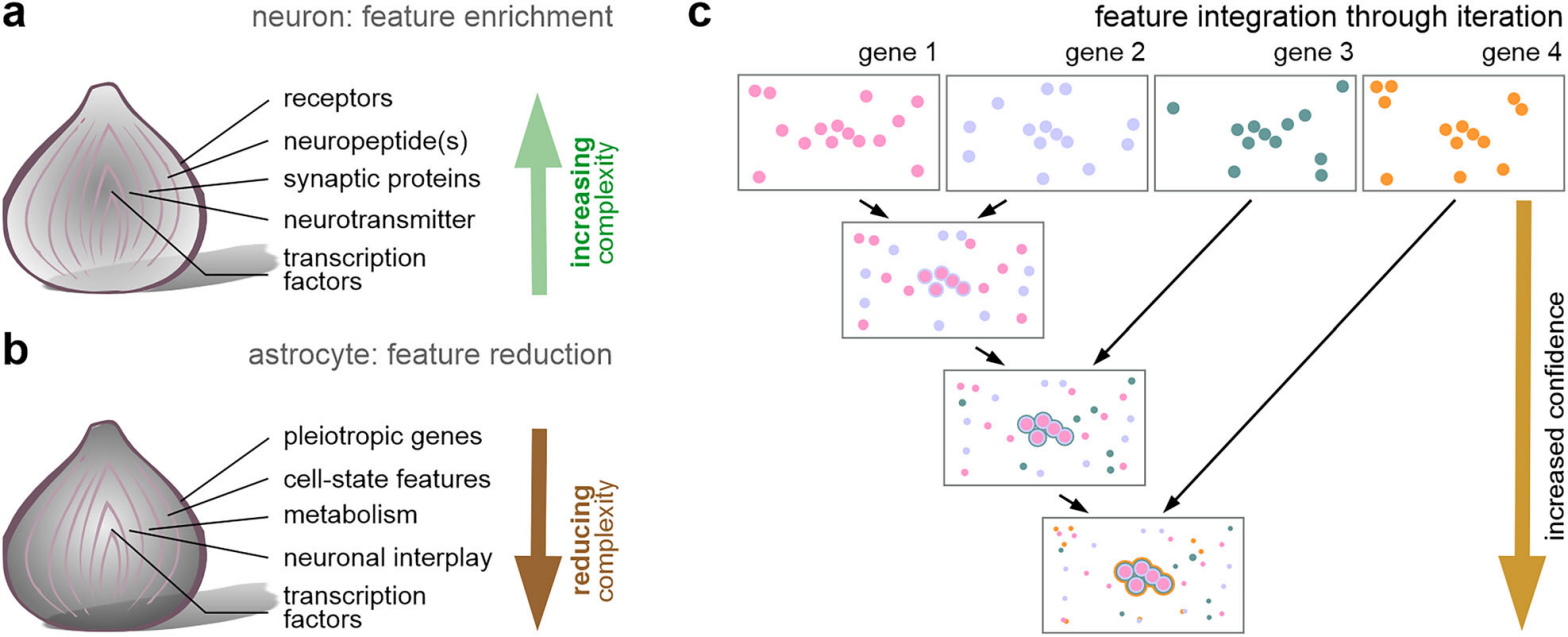
A feature-selection approach to interrogate the molecular make-up of astrocytes. **a** Single-cell biology for neurons stipulates an enrichment approach built on the cohesive alignment of gene regulatory networks driving cell identification developmentally, to the molecular underpinnings of neurotransmitter and neuropeptide systems that set neurons apart from their peers. **b** Such gene enrichment is unlikely to suffice for astrocytes because their most distinct gene regulatory networks are related to cellular metabolism and homeostatic adaptation. Therefore, a reductionist approach, in which layers of complexity are sequentially removed, could reveal genuine transcriptionally distinct and positionally segregated subtypes through bona fide subtype-defining genetic features. **c** Feature-based reconstruction of astrocyte identity. On average, five or more unique features specify spatially segregated astrocytes at a confidence level of >98%. Thus, by iteratively overlaying genes through either single-molecule fluorescence in situ hybridization or spatial transcriptomics, can reveal spatially segregated astrocyte subsets. Note that the addition of each gene increases fidelity while proportionately reducing noise.

The above gaps in appreciating astrocyte diversity arguably reflect the neuron-centric strategies for the molecular interrogation of cellular features used today, ranging from genetic access to astrocyte subgroups and their interrogation by chemical probes or optical stimulation. A restriction that long existed in most algorithms for the analysis of single-cell RNA-seq data is dependence on the gradual enrichment (aggregation) of features for cell annotation when performing unsupervised feature selection based on variability. For neuronal features, a priori assumptions include (1) regionally specific transcriptional signatures, (2) stable developmental endpoints (that is, no dedifferentiation and activity-dependent neurogenesis), (3) stable subsets of function-defining markers, be these neurotransmitter transporters, metabolizing enzymes or neuropeptides^61,62^, and (4) receptor repertoires for prospective circuit embedding (Fig. 4a). These characteristics do not apply to most astrocytes, except for a few specialized subtypes (for example, tanycytes, ependymocytes that line the walls of the ventricles and central canal, and Bergmann and Müller glia^63^).

It is therefore appealing to harness a reverse approach that relies on the stepwise restriction of features^64^ (Fig. 4b). First, the removal of genes indiscriminately expressed by astrocytes to retain their cytoarchitectural and fundamental signaling layouts (for example, *S100b*) or to encode their generic functions (for example, neurotransmitter metabolism and uptake (*EAAT1,2*, *Adcyap1*, *Glul*, *Aqp4* and *Slc38a3*), syncytial connectivity (*Gja1*), energy metabolism (*Apoe*, *Aldh1l1* and *Aldoc*) and trophic factors (*Ntrk2*, *Egfr* and *Fgf3*) eliminates ‘stability bias’. Second, context-dependent genes that are historically called ‘inducible’ can be filtered out by comparing RNA-seq datasets on astrocytes under experimental versus control conditions, regressing out inducible genes that either change stochastically from one experiment to another (for example, *Ntrk2* and *Apoe*) or are commonly associated with specific activity states (for example, *Gfap* and *Fos*). At this point, the remaining gene set integrates both developmental information—such as transcriptional signatures that characterize distinct proliferative domains—and spatial information, including gene regulatory networks restricted to anatomical loci, along with a flexible repertoire of metabolic and allostatic genes. In other words, such regressive analysis reveals stable molecular marks unifying relatively small numbers of astrocytes that otherwise would remain deeply buried in the conventional RNA-seq data. Henceforth, the expression of stable yet divergent gene regulatory networks can be assigned to specify locations without being negated due to the ‘dilution effect’ of direct unsupervised analysis. By analogy, astrocyte identities might be best resolved by consecutively peeling away layers—like an onion—until the core structure is revealed (Fig. 4b). Once reaching the innermost make-up of astrocytes, not only homeobox genes but also those that are harmonious with the roles of local circuits (hormone receptors, neuropeptide receptors and synapse-specific neuromodulatory units) may define the genuine spatial heterogeneity of astrocytes. Nevertheless, single genes will not suffice to segregate and spatially confine astrocytes. Instead, an iterative process of overlaying core genes should be used to achieve sufficient confidence in identifying molecularly and spatially segregated stable astrocyte subclasses (using five or more genes can provide near-absolute confidence in regions where a high-fidelity reference is available; Fig. 4c).

Thus, efforts of the neuroscience community through molecular and experimental profiling of astrocytes open opportunities to redefine ‘astrocyte identity’ by using an iterative classifier. This approach recognizes astrocytes as progeny of radial glia—born after neurons—and retaining unique positional genes that enable their regionalization, allowing them not only to co-evolve with neurons derived from the same progenitor niche but also to optimally support specialized circuit functions. It is no longer surprising, therefore, that focal silencing (or ablation) of astrocytes renders neurons inapt to control specific behaviors, causally implicating astrocytes in driving higher-order brain functions^65–68^.

## Astrocytes dynamically control excitation and inhibition of neuronal networks

Coordination of glutamatergic (mainly excitatory) and GABAergic (mainly inhibitory) inputs is of fundamental importance for the activity of neuronal networks and therefore required for normal cognitive function. Both processes localize to the plasmalemma and arise from the activity of ion channels and ionotropic receptors, both being further tuned by a panoply of metabotropic receptors. Maintaining stable electrochemical gradients and preserving ion homeostasis in the brain are of fundamental importance for proper neuronal function and cognitive processes. The activity of ion channels is tightly regulated by transmembrane ion gradients, and even modest fluctuations in extracellular ion concentrations can markedly affect neuronal excitability and synaptic transmission and integration. Astrocytes are central for regulating the ion composition of interstitium, the ionostasis, which is intimately associated with neuronal excitability. Recent in vivo mouse studies demonstrate that the composition of interstitial ions shifts according to the prevailing brain state. During wakefulness, extracellular K⁺ concentration is increased, whereas Ca²⁺ and Mg²⁺ concentrations are decreased; the reverse is observed during sleep^69^. Astrocytic cytosolic Cl⁻ concentration ([Cl⁻]_i_) also undergoes marked state-dependent variations: [Cl⁻]_i_ remains consistently high during sleep but experiences pronounced fluctuations in wakefulness, culminating in lower mean levels of [Cl⁻]_i_ (ref. ^70^).

Voltage-gated ion channels that mediate membrane excitability and action potential generation and propagation are highly sensitive to ion gradients and membrane polarization; even relatively small fluctuations in extracellular K^+^ concentration affect membrane potential and, hence, neuronal excitability. Astrocytes control extracellular K^+^ primarily through the astrocyte-specific α2-subunit containing Na^+^–K^+^ pump and astrocytic inward rectifying K_ir_4.1 K^+^ channels^71^. Inhibition (that is, membrane hyperpolarization) in the adult CNS is mediated by Cl^−^ ions fluxes through several types of Cl^−^ channels and ionotropic GABA and glycine receptors. Cytosolic Cl^-^ in neurons is low (~5 mM), and hence, Cl^−^ channels mediate Cl^−^ influx and hyperpolarization, while the long-lasting activity of these channels depletes the extracellular Cl^−^. Astrocytes to the contrary have high cytosolic Cl^−^ (30–50 mM)^70,72^, and when astrocytic Cl^−^ channels are opened (incidentally, astrocytic perisynaptic membranes contain GABA_A_ receptors) Cl^−^ efflux is generated to maintain high extracellular Cl^−^ concentration and sustain inhibitory transmission^70^.

Synaptic transmission, a fundamental aspect of neuronal coordination and cellular excitability, is dependent on both glutamatergic (excitatory) and GABAergic (inhibitory) systems. These systems are regulated by astrocytes, which, through the glutamate(GABA)–glutamine shuttle, remove extracellular glutamate and supply neurons with glutamine, from which both glutamate and GABA are synthesized^73–75^. At glutamatergic synapses, astrocytes remove the majority of the released glutamate by excitatory amino acid transporters 1 and 2 (EAAT1 and EAAT2/SLC1A2 and SLC1A3)^75^, which is crucial for maintaining synaptic transmission^76,77^.

At the same time, glutamate can be released from astrocytes^78^ through several mechanisms including exocytosis^53,79^, Sxc^−^ cystine/glutamate antiporter^80^ or diffusion through plasmalemmal channels, such as Bestrophin-1 (Best-1) anion channels or two-pore-domain potassium channel TREK-1^81^. Astrocytes also regulate the neuronal excitability by modulating tonic *N*-methyl-D-aspartate (NMDA) receptor currents, by releasing glycine and contributing to extracellular level of D-serine, both being allosteric co-agonists of NMDA receptors through the release of glutamate and contributing to extracellular levels of NMDA receptors^82,83^. Tonic NMDA receptors currents increase intrinsic neuronal excitability by facilitating action potential generation and integration of dendritic excitatory inputs^84^; similarly, astrocytes can release homocysteic acid, which acts as an agonist of NMDA receptors^85,86^.

Astrocytes mediate tonic GABA inhibition in several brain regions, including the thalamus and cerebellum^87,88^, and supply extracellular Cl^−^ to maintain inhibitory Cl^−^ currents^70^. It is also noteworthy that the astrocytic cytosolic Cl⁻ concentration [Cl^−^]_i_ varies not only with wake–sleep cycles but also in response to movement onset and sensory stimulation^70^. Such dynamic changes highlight the importance of tightly maintained astrocytic Cl− homeostasis for supporting normal neuronal function, especially under prolonged or repetitive bouts of synaptic activation and inhibition. Although the molecular machinery responsible for regulating astrocytic [Cl^−^]_i_ and orchestrating its brain-state dependence has yet to be fully deciphered, a deeper understanding of these processes may offer valuable therapeutic avenues for modulating brain states and addressing a variety of neurological disorders.

**Fig. 5:**
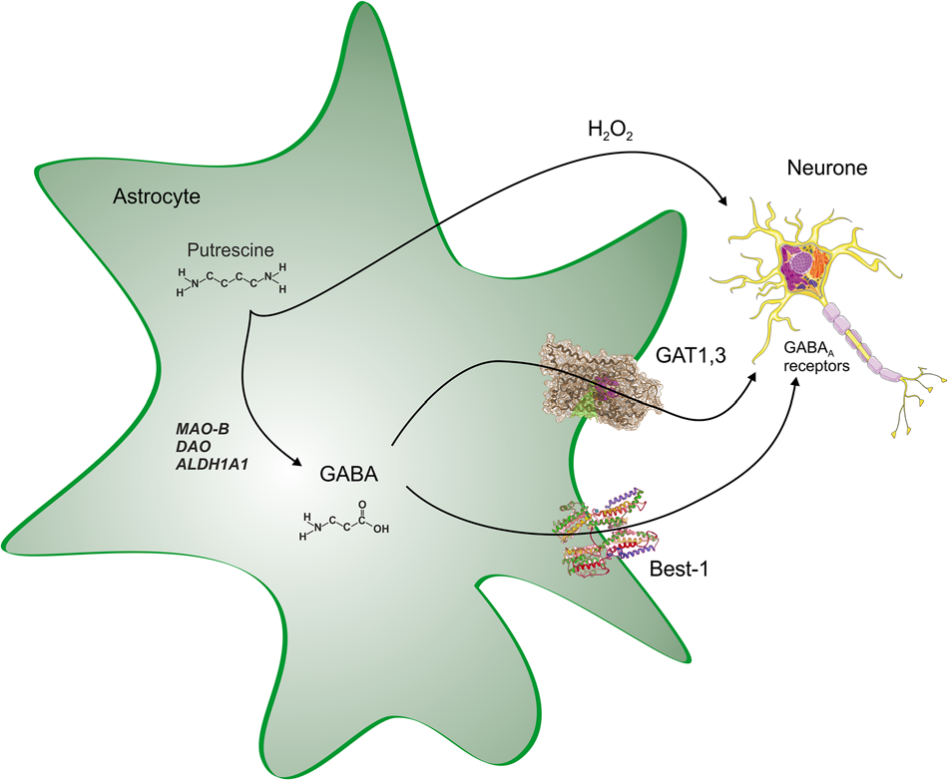
Synthesis of GABA from putrescine in astrocytes. See text for further details.

In the healthy brain, astrocytes contribute to tonic inhibition by synthesizing and releasing GABA through nonsynaptic mechanisms. Astrocytic monoamine oxidase B (MAO-B) and diamine oxidase (DAO) catalyze the conversion of putrescine to GABA (Fig. 5). Release of GABA from astrocytes is mediated by nonvesicular pathways, including diffusion through Best-1 channels or through reversed GABA transporters; astrocyte-derived GABA acts through extrasynaptic GABA receptors. The Best-1 channels are localized in astrocytic leaflets and are opened by relatively moderate increases in [Ca^2+^]_i_ at the resting membrane potential^89^; notably, Best-1 channel also localize to the plasma membranes of neurons^90,91^. Astroglial GABA transporters (mainly GAT3/SLC6A11^92^) contribute to tonic inhibition by regulating extracellular GABA levels. While GAT3 is primarily responsible for GABA uptake, it can also function in reverse mode to release GABA^93–95^. The reversal of GAT3 is controlled by [GABA]_i_, membrane potential and [Na^+^]_i_: both depolarization and an increase in [Na^+^]_i_ favor the reverse mode^20^. Neuronal tonic GABA currents tend to reduce neuronal excitability, decrease neuronal spiking and refine information processing by dampening excitatory potentials^88,96–98^; transporter-mediated GABA release has been implicated in this tonic inhibition^99–102^, although some studies suggest that such a mechanism may not be physiologically relevant^103,104^. Moreover, GABA transporters are expressed in oligodendrocytes and microglia, which may also contribute to extracellular GABA dynamics^105,106^.

Because astrocytic [Cl^−^]_i_ is critical for regulating neuronal inhibition, a key question arises: are [Cl^−^]_i_ shifts merely a byproduct of distinct neuronal activity patterns in different brain states, or do they actively shape neuronal excitability on a state-dependent basis? The equilibrium potential of GABA_A_ receptors (*E*_GABA_, which is essentially *E*_Cl_) in pyramidal neurons varies with the time of day, shifting toward hyperpolarization during sleep and adopting more depolarized values in wakefulness^107^. This shift in *E*_GABA_ hinges on neuronal Cl⁻ gradients, governed by the levels of [Cl⁻]ᵢ and [Cl⁻]ₒ. Sleep deprivation induces upregulation of the main Cl⁻-accumulating transporter, NKCC1/SLC12A2, resulting in increased neuronal Cl⁻ accumulation—a reversible process modified by the NKCC1 inhibitor bumetanide^107^. At the same time, *E*_GABA_ also depends on [Cl⁻]ₒ in the synaptic cleft, which is regulated by astrocytic Cl⁻ transport mechanisms and astrocytic Cl⁻ release. Elevated astrocytic [Cl^−^]_i_ during sleep sustains a more hyperpolarized *E*_GABA_, whereas decreased [Cl^−^]_i_ in wakefulness restricts Cl⁻ availability, thereby shifting *E*_GABA_ to more positive values. These findings highlight the fundamental role of astrocytes in coordinating glutamate, GABA and Cl^−^ to adjust neuronal excitability across brain states^70^.

To summarize, astrocytes possess multiple pathways contributing to the regulation, both rapid and long-term, of neuronal excitability.

## Astroglia, neuroprotection and cognitive reserve

Astroglia-driven homeostatic pathways are central for neuroprotection, through both core homeostatic mechanisms and inducible responses to environmental challenges, stress and pathological insults. Moreover, astrocytes contribute to—and to a large extent define—cognitive reserve, which in turn influences the neurological and cognitive outcomes of all CNS disorders as well as physiological aging. The concept of cognitive reserve was introduced by Yaakov Stern^108,109^ to account for a well-known absence of direct correlation between the degree of damage to the CNS and cognitive as well as neurological outcome. In particular, cognitive reserve defines cognitive longevity in aging and neurodegenerative disorders.

Cognitive reserve is made from several components: (1) the brain reserve (which reflects the anatomical individual differences quantified by the number of neurons and synapses), (2) the brain maintenance supported by all homeostatic pathways from cellular to organ level), (3) the brain resilience (the ability to withstand stress without mounting the pathology) and (4) the brain compensation directly linked to the regenerative capacity of the nervous tissue. All these components are shaped by the interplay between genetic factors and lifelong experience: in particular, physical exercise, healthy diet and intellectual engagement increase cognitive reserve, whereas diseases and stressors that lead to the accumulation of pathological burden decrease it^110–112^.

Homeostatic, protective and regenerative functions of neuroglia are central for defining the cognitive reserve^113^. Astrocytes, in particular, contribute to the brain reserve through life-long neurogenesis^114,115^ as well as through initiating and regulating synaptogenesis, synaptic maturation and synaptic extinction^52,116,117^. Astrocytes are central to the brain maintenance through (1) controlling ionostasis of major ions (Na^+^, K^+^, Ca^2+^ and Cl^−^) that define neuronal excitability; (2) providing clearance of major neurotransmitters (glutamate, GABA, and monoamines) and supplying neurons with obligatory precursors of neurotransmitters and neuromodulators (glutamine and L-serine); (3) providing neural cells with energy substrates; and (4) limiting oxidative stress through contributing to biosynthesis of glutathione and recycling of ascorbic acid^113^. Through all these mechanisms, astrocytes also support synaptic transmission and synaptic plasticity. Finally, astrocytes are fundamental for brain resilience and brain compensation through numerous neuroprotective systems (again glutamate clearance, K^+^ buffering and ROS scavenging), through reactive astrogliosis, which protects the nervous tissue against acute lesions and limits chronic disorders^118^, and through supporting postlesional regeneration^119,120^.

## General pathophysiology of astroglia

### Classifying astroglial pathophysiology

The role of astrocytes in neuropathology is complex and mutable; astrocytes may undergo multiple progressive and/or regressive changes, exhibiting numerous pathological phenotypes that can coexist within the same disorder, be context- or region-specific, or influence one another during disease progression. Astroglial pathophysiology can be broadly classified into: (1) astroglial reactivity or reactive astrogliosis; (2) astroglial atrophy and functional asthenia; (3) astroglial degeneration and death; and (4) astrocytopathies with aberrant pathological astrocytes^9^.

Reactive astrogliosis (from Greek *glia* and *-osis*, meaning ‘glial process’), which forms a barrier between the brain parenchyma and acutely injured tissue, was described in detail by Pío del Río-Hortega and Wilder Penfield in the 1920s^121–123^. Reactive astrogliosis is an evolutionarily conserved, graded, context-specific and multistage defensive response of astrocytes to neuropathology governed by complex molecular programs that translate into biochemical, morphological, metabolic and physiological features of astroglia, leading to an upregulation or loss of homeostatic cascades, or to the gain of new protective or regenerative functions^9,118^. There are many distinct or converging reactive phenotypes with idiosyncratic transcriptomic and molecular signatures^124–128^; the popular dichotomic division into A1–A2 astrocytes is misleading and has been refuted by the glial community^118^. Conceptually, reactive astrogliosis is subclassified into (1) proliferative, anisomorphic (that is, with loss of territorial domains and profound morphological remodeling), and (2) nonproliferative isomorphic (that is, with preservation of territorial domain organization). The former is the feature of inflammatory process in response to brain trauma of various etiologies (mechanical, ischemic, bacterial and autoimmune) and results in the formation of perilesional borders, and ultimately of the glia limitans perilaesiones. The latter contributes to pathogenesis of chronic neurological disorders^9,129–132^. This classification is very broad, and it does not account for the diversity of reactive phenotypes; in-depth characterization of the latter is needed for more precise stratification of astrocytic reactivity.

Astroglial structural atrophy and functional asthenia (from Greek *ἀσθένεια*, meaning lack of strength, weakness or feebleness, that is, loss of function) is widely present across neurological disorders. Asthenic and atrophic changes are accumulating with aging^133^, thus reducing neuroprotection and lowering brain resilience and therefore increasing the susceptibility to age-dependent neurodegenerative disorders. Structural atrophy of astrocytes leads to a decrease in synaptic coverage and synaptic maintenance thus affecting both excitatory–inhibitory balance and synaptic plasticity^134,135^. Atrophy of astrocytic endfeet leads to the loss of perivascular coverage and compromises the blood–brain barrier^136^. Deficient glutamate clearance drives neuronal damage in neurotoxic disorders such as Wernicke–Korsakoff encephalopathy or hepatic encephalopathy^137–139^, whereas diminished K^+^ buffering together with insufficient glutamate clearance leads to spreading depression, migraine and epilepsy^140–142^. Structural atrophy of astrocytes is a common sign of stress-induced depression, while manipulation with plasmalemmal linker ezrin, which controls the extension of peripheral astrocytic leaflets, alleviates depressive-like behaviors^143–147^. Functional deficiency of astrocytic glutamate clearance and K^+^ buffering is the primary cause of neuronal damage in neurodegenerative diseases, most notably in amyotrophic lateral sclerosis (ALS) and Huntington’s disease (HD)^148–153^, whereas functional asthenia of astrocytes may exacerbate β-amyloid pathology in the context of Alzheimer’s disease (AD)^154^. Fundamentally, the loss of astrocytic support and neuroprotection rather than the emergence of ‘toxic’ phenotypes takes a leading role in mediating neuronal damage and death across all types of neuropathology.

Astrocytopathies cover the yet poorly characterized pathological changes leading to the emergence of aberrant astrocytes that act as primary pathophysiological entities driving the disease. These are represented by genetic astroglial leukodystrophies, in which aberrant astrocytes fail to support myelination, leading to profound lesions of white matter^155^. Aberrant astrocytes expressing markers of both astrocyte and microglia have been detected in ALS, in stroke and in dementia with Lewy bodies^156,157^. Finally, degeneration of astrocytes leads to profound morphological changes, including process fragmentation or clasmatodendrosis, signaling irreparable damage and cell death; clasmatodendrosis is observed in several neuropathologies, including infections, trauma and neurodegeneration^158^.

### Rethinking CNS scarring

In contrast to the healthy brain, where astrocytes have historically struggled for recognition against the neuronocentric perspective, their role in the CNS scarring following brain tissue damage has been often overstated. For much of the last century, the term ‘glial scar’ has dominated the discourse on this topic. While this term includes microglia and oligodendrocyte precursor cells, it has primarily been used to describe reactive astrocytes flanking the lesion border. Regrettably, the glial scar became an oversimplified descriptor for the entire CNS scar, overlooking the critical contribution of the fibrotic component.

Another long-standing misconception was that the astrocytic ‘scar’ impedes tissue repair and axonal regrowth. However, recent findings indicate that reactive astrocytes play a protective role by containing damage and preventing its spread beyond the lesion site^159,160^. Injuries to the CNS often compromise the integrity of the blood–brain or blood–spinal cord barriers, leading to increased permeability, immune cell infiltration, and the release of inflammatory mediators that exacerbate neurodegeneration. Astrocytes are essential in re-establishing brain barriers after injury^159^. Furthermore, border-forming astrocytes can extend their processes across the lesion, creating a scaffold that bridges the fibrotic scar and facilitates axonal regeneration^161–163^.

This evolving perspective has shifted attention beyond the astrocytic ‘scar’, recognizing the fibrotic core as a distinct component of CNS scarring^119^. In larger lesions, such as ischemic brain injuries or traumatic spinal cord damage, fibroblasts and macrophages heavily populate the lesion site^164,165^. Unless the injury breaches the meninges, scar-forming fibroblasts originate from perivascular sources within CNS tissue. These perivascular fibroblasts, along with pericytes, are recruited locally in a lesion-dependent manner^166^. Fibroblast activation and extracellular matrix deposition are crucial for wound closure and tissue integrity restoration, as well as to the formation of fibrotic scar formation in the adult mammalian CNS^164,167^. Notably, the African spiny mouse remains the only known exception, displaying an alternative regenerative response without persistent fibrotic scarring^168,169^.

Following wound contraction, scar formation is orchestrated through complex astrocyte–fibroblast interactions. Reactive astrocytes express ephrin-B2, while stromal fibroblasts express the congruent receptor EphB2, guiding the formation of a distinct lesion border that segregates glial and fibrotic compartments^170^. Border-forming astrocytes are recruited locally through the remodeling of astrocytes in the vicinity of the lesion, which proliferate, become reactive and upregulate glial fibrillary acidic protein (GFAP)^171^. After spinal cord injuries, border-forming astrocytes may also arise from proliferating ependymal cells, contributing to the formation of a protective glial border^172,173^. These astrocytes secrete growth factors that mitigate secondary damage and promote tissue stabilization^174^. Experimental reduction of border-forming astrocytes leads to an expansion of the fibrotic scar core and further limits axonal regeneration^161,174,175^. Conversely, attenuation of fibrotic scarring enhances axonal regeneration and improves sensorimotor function recovery^176^. A complete blockade of the fibrotic response, however, impairs wound healing and results in structural tissue defects^167^.

This refined understanding of CNS scarring underscores the dynamic roles of both astrocytes and fibroblasts, shifting the paradigm from a simplistic view of the glial scar to a more nuanced appreciation of the interplay between glial and fibrotic components. Future research will be critical in elucidating how these cellular interactions can be modulated to optimize recovery and regeneration after CNS injury.

## Astrocytes in CNS disorders

Cognitive impairment is a frequent outcome of a wide range of disorders, which affect individual’s ability to think, concentrate, remember or make decisions. In this Review, we focus specifically on diseases of the CNS directly linked to cognitive disturbances. Astrocytes are the main actors in all neurological, neurodegenerative and psychiatric diseases (Table 1). Here, we present a brief account of the major pathological roles of astrocytes in several diseases affecting cognition. We included aging as the main risk factor for cognitive disorders; we also included neuropathic pain, which, of course, has peripheral roots, but the main pathology is localized to the spinal cord. Neuropathic pain impairs cognitive abilities through persistent suffering and is largely driven by astrocytic pathology.

**Table 1:** Astrocytes contribution to diseases of cognition with an emphasis on targetable astrocyte-specific molecules and pathways.

Disease/condition	Astroglial contribution	Targetable molecules	References
Physiological aging	Significant reduction in the size, territorial domain, volume and complexity of astrocytes in old animals associated with downregulation of Ezrin, reduction in astrocytic homeostatic cascades including glutamate(GABA)-glutamine shuttle, K^+^ buffering, Ca^2+^ waves propagation in astrocytic syncytium, anti-oxidative defense, cholesterol synthesis, reactive response to damage, water transport, glymphatic system, Increase in GABA synthesis, ROS production and tonic inhibition. Decline of LTP and synaptic plasticity.	EAAT1,2, MAO-B, Ezrin, AQP4, anti-oxidative pathways, glutathione synthesis cholesterol synthesis	^133–135,640^
Neuroinflammatory diseases			
Acute penetrating neurotrauma	Proliferative anisomorphic reactive astrogliosis plays a central role in the pathogenesis of acute neurotrauma. Reactive astrocytes form a perilesional barrier assisting wound closure, resolution of neuroinflammation and postlesional regeneration. Over time, barrier astrocytes form glia limitans perilesiones.	GFAP? Glutathione? MAO-B? Cx43 hemichannels	^120,641^
Diffuse and chronic neurotrauma	Nonproliferative isomorphic astrogliosis with mild hypertrophy. Functional asthenia with loss of glutamate transporters, K_ir_4.1 channels, GS and connexin 43; loss of support of blood–brain barrier	EAAT2, K_ir_4.1 channels, Cx43 hemichannels	^642,643^
Neuropathic pain	BDNF derived from reactive microglia instigates decrease in neuronal KCC2 expression, thus leading to a depolarizing shift in *E*_Cl−_ and turning GABA into an excitable transmitter. Reactive astrocytes contribute to GABA tone through upregulation of MAO-B expression and release various pathogenetic factors through Cx43 hemichannels. Reactive astrogliosis and resistance to neuropathic pain are regulated by astrocyte-specific G protein-coupled receptor 37-like 1 (GPR37L1) Maresin1 receptor	BDNF, TrkB receptors, MAO-B, Cx43 hemichannels, GPR37L1	^265,506,541,644–647^
Stroke/ischemia	Astrocytes form a perilesional barrier and support neuronal survival in the penumbra. Expression levels of EAAT1 and EAAT2 are critical for neuronal protection in the penumbra, whereas ischemia can lead to their downregulation. Astrocytic Cx43 hemichannels contribute to CNS damage during reperfusion.	EAAT1,2; Cx43 hemichannels	^510,648–651^
Autoimmune diseases: multiple sclerosis and neuromyelitis optica	In MS astrocytes undergo reactive remodeling and contribute to the formation of lesions through perilesional barriers. The NMO is a primary astrocytopathy caused by anti-AQP4 antibodies attaching to the astrocytic endfoot. The complement system takes the leading role in subsequent cellular damage. Astrocytic APOE-containing EVs exert neuroprotection in the context of NMO.	C1q, C3, APOE, astrocytic EVs	^187,652–655^
Autoimmune astrocytopathies	Meningoencephalitis; primary astrocytopathy caused by autoantibodies against GFAP and vimentin.	GFAP, Vimentin	^258,260,261,263^
Neurodegenerative diseases			
Alzheimer’s disease	Reactive and atrophic astrocytes coexist; their populations expand and shrink in the course of disease progression. Reactive astrocytes (together with reactive microglia) concentrate around senile plaques, arguably forming protective barriers. Reactive astrogliosis is nonproliferative and isomorphic. Reactive astrocytes upregulate MAO-B-dependent GABA synthesis (to counteract neuronal hyperexcitability) and the urea cycle (to degrade β-amyloid). Both pathways are linked to an increased production of H_2_O_2_ causing neuronal damage. Reactive astrocytes demonstrate an increase in Cx43 hemichannel opening, possibly contributing to neurotoxicity. Astrocytic reactivity changes in the course of AD, being high in early stages and then waning in advanced stages, possibly limiting neuroprotection.	GFAP (?) MAO-B, enzymes of the urea cycle (ornithine decarboxylase 1, ODC1); ROS scavengers, Cx43 hemichannels; α7 nACh receptors	^193,489,545,547,559,656–659^
Amyotrophic lateral sclerosis	ALS is a primary astrocytopathy. Astrocyte-specific silencing of the mutant SOD1^G93A^ gene alleviates ALS symptoms and prolongs lifespan. Grafting of SOD1^G93A^ astrocytes to the healthy spinal cord triggers ALS-like pathology. ALS is characterized by multiple pathological astrocytic phenotypes, including reactive, degenerating, aberrant and atrophic. Neuronal death is precipitated by failure in glutamate clearance due to profound downregulation of EAAT1 expression, by oxidative stress and by possible release of molecules inducing neuronal hyperexcitability through Cx43 hemichannels.	EAAT1,2, Cx43 hemichannels, antioxidant system, glutathione	^240,241,244,245,247,248,660^
Parkinson’s disease	Astrocytic atrophy and reactivity, decreased transmitophagy and oxidative stress.	Antioxidant system, glutathione, MAO-B, Cx43	^208,209,531^
Huntington’s disease	Astrocytopathy is manifested by morphological atrophy, decreased expression of K_ir_4.1 channels, and glutamate transporters.	K_ir_4.1, EAAT1,2	^150–153,219,661^
Frontotemporal dementia	Pathological astrocytes are distinguished by the expression of a specific marker, WDR49; pathological astrocytes form clusters. Some signs of reactive astrogliosis.	WDR49	^225,226,230,231^
Astrocytotauopathies	Primary astrotauopathies, characterized by tau accumulation exclusively in astrocytes are classified, according to histopathology, into (1) astrocytic plaques, (2) tufted astrocytes, (3) ramified astrocytes, (4) globular astroglial inclusions, (5) thorn-shaped astrocytes and (6) granular/fuzzy astrocytes. Cellular pathophysiology is unknown.	??	^253,254,256,257^
Neuropsychiatric diseases			
Schizophrenia	Delayed maturation and astrocytic atrophy, oxidative stress and regulation of GABA.	BMP, SMAD4, REST	^299,302–304^
Major depression; stress-related depression	Morphological atrophy of astrocytes is the primary histopathology in human patients and in stress-induced animal models of depression. Manipulations with astroglial ezrin in depression relevant areas confer resistance to stress-induced depression in mice.	Ezrin, Cx43, EAAT1,2	^145,146,272,280,281^
Post-traumatic stress disorder	Reactive astrogliosis (triggered by the traumatic injury) is prevalent. Mental PTSD is dominated by astrocytic atrophy, detected both in animal models and in human patients (by post-mortem immunohistochemistry and PET imaging of MAO-B). Astrocyte-derived IL-1β can contribute to fear learning. A significant decrease of astrocyte-specific Ca^2+^-binding protein S100B was also detected in post-mortem tissue of PTSD sufferers.	S100B, MAO-B	^370,662,663^
Epilepsy, migraine and spreading depression			
Epilepsy	Astrocytic pathology is manifested by decreased K^+^ buffering linked to a decreased expression of K_ir_4.1 channels or their loss of function mutations. Epilepsy is also associated with a deficient glutamate–glutamine (and GABA–glutamine) shuttle, reflected by a 20–40% decrease in EAAT1 and EAAT2 expression and downregulation of GS, as observed in both post-mortem human samples and experimental animal models. Epilepsy is also associated with the loss of endfeet localization of AQP4 water channels, downregulation of Cx43 connexins, and uncoupling of the astrocyte syncytium. Aberrant Cl^-^ homeostasis in astrocytes may also contribute to the pathogenesis of seizures.	EAAT1,2; GS, K_ir_4.1, AQP4, Cx43	^141,142,309,313,316,319,321,664,665^
FHM type 2, spreading depression	FHM type 2 is a primary astrocytopathy linked to a loss-of-function mutation of the astrocyte-specific α2 subunit of the Na^+^-K^+^ ATPase (NKA). In spreading depression, both reduced astrocytic glutamate clearance and K^+^ buffering contribute to pathogenesis.	α2NKA, EAAT1,2	^328,329,666^
Astroglial leukodystrophies			
Alexander disease	Sporadic mutations of GFAP leading to severe damage of white matter. Compromised glutamate uptake.	GFAP, gene therapy	^331,667,668^
Vanishing white matter disease	Atrophic astrocytes with reduced complexity and aberrant, blunt and coarse processes are mainly localized in the white matter. Expression of mutated eIF2B genes, causal for the disease reduce the number of GFAP-positive astrocytes, impairs GFAP-related structures, suppresses expression of S100B, prevents maturation of astrocytes and results in loss of astrocytic functions.	Causal genes *EIF2B1*, *EIF2B2*, *EIF2B3*, *EIF2B4* and *EIF2B5*) encoding the eukaryotic translation initiation factor eIF2B	^335–337,669–671^
Megalencephalic leukoencephalopathy with subcortical cysts	Primary astrocytopathy is caused by mutant *MLC1* or *HEPACAM* (also known as *GlialCAM*) genes. Pathological astrocytes demonstrate deficient K^+^ buffering, with malfunctioned NKA and K_ir_4.1, deficient glutamate uptake and AQP4-dependent water transport.	Causal genes: *MLC1, HEPACAM* NKA, EAAT2, K_ir_4.1, *AQP4*	^339,342,343^

*LTP* long-term potentiation, *NMO* neuromyelitis optica, *PTSD* post traumatic stress disorder.

### Aging

Physiological aging is not a pathology, and yet it is the major risk factor for many diseases of cognition, most notably for neurodegeneration. Aging could be defined as a progressive decline of structural integrity and functional capacity of all organs and systems that weakens organism defenses and increases the vulnerability to diseases. Physiological aging is characterized by a general decline in neuroglial function^177^, and astrocytes follow this trend. Cell-specific transcriptomics reveal pronounced age-dependent changes in glial gene expression, whereas the neuronal transcriptome shows only minor changes^178–180^. The number of astrocytes does not change in old brains of humans, marmosets and rodents^181–183^; however, the size and complexity of cortical and hippocampal astrocytes decrease substantially with age^134,135,183^. A decrease in astrocytic size, territorial domain, complexity and synaptic coverage impairs homeostatic support of synaptic transmission thus affecting synaptic plasticity^133,134^. All major homeostatic functions of astrocytes—including metabolic support, neurotransmitter and precursor homeostasis and metabolism, cholesterol synthesis, water transport, support of the glymphatic system, neurogenesis, neuroprotection and defense—decline with aging^133,182^.

### Acute neurodegeneration—neurotrauma, stroke, autoimmune diseases and infection

Acute traumatic lesions of the CNS trigger neurological and cognitive symptoms directly linked to the death of neural cells—particularly neurons and oligodendrocytes—destroying both information-processing units and the connectome; thus, such lesions can be considered a form of acute neurodegeneration. These lesions are caused by mechanical, ischemic, infectious or autoimmune attack and are fundamentally characterized by prominent neuroinflammation. Astrocytes are key cellular elements of acute brain trauma, which triggers proliferative, isomorphic astrogliosis^9,118,130,131^. The peak of astrocyte proliferation at the trauma perimeter occurs 2–7 days after the traumatic event; over time, this proliferation gradually subsides^184^. Reactive astrocytes form the perilesional border essential for wound closure, proper scar formation and postlesional regeneration; after the resolution of neuroinflammation, astrocytes form glia limitans perilaesiones^185^.

Astrocytes begin responding to an injury as early as 3 h after ischemic insult. By 3 days post-ischemia, astrocytes exhibit strong reactive phenotypes, including high inducible nitric oxide synthase (iNOS) expression and pronounced morphological changes. Around day 5, astrocytes elongate and maintain iNOS expression, and by day 7, they start to organize a barrier structure, which becomes fully matured within approximately 1 month^186^. During this process, astrocyte-derived type I collagen is upregulated, coinciding with the onset of neuronal death. SPARC (secreted protein acidic and rich in cysteine), a critical regulator of collagen synthesis, cell–matrix interaction and tissue remodeling, is co-expressed with type I collagen during this phase. In vitro experiments using separate primary cultures of astrocytes and cortical neurons demonstrated that type I collagen induces neuronal death through integrin signaling pathways.

In multiple sclerosis (MS), reactive astrocytes similarly surround the lesions, form borders and support the formation of fibrotic scar, although astrocytes present asthenia and loss of homeostatic functions^187,188^. In neuromyelitis optica, astrocytes are primary targets for anti-AQP4 antibodies, and thus undergo death and loss of function; the secondary astrogliosis may be generated around lesions^189,190^. A wall of reactive astrocytes also surrounds brain abscesses, with reactive astrogliosis being a prominent feature of brain infectious damage^191^. Diffuse astrocytic reactivity is also observed in sepsis-induced encephalopathies^132^.

### Chronic neurodegeneration

#### Alzheimer’s disease

Astrocytes undergo complex and spatially segregated changes in the course of AD; these changes include reactive remodeling, atrophy with functional asthenia, astrocyte degeneration and clasmatodendrosis^192–194^. Extracellular deposition of β-amyloid and formation of plaques trigger reactive nonproliferative isomorphic astrogliosis. Reactive astrocytes surround senile plaques (both in post-mortem tissues and in animal models) forming (together with microglia) a loose perimeter distinct from astrocytic palisades in traumatic brain injury. These reactive glial barriers are, arguably, protective against β-amyloid toxicity^195,196^, whereas suppression of reactive astrogliosis exacerbates pathology in AD animal models^197^. Atrophic astrocytes have been detected in human post-mortem brains in all Braak tau disease stages of AD, while the loss of astrocytic homeostatic functions contributes to AD pathogenesis^133,198,199^.

Astrocytes are sensitive to oxidative stress and the astrocyte enzymes glutamine synthetase (GS) and brain creatine kinase (CKB) are among the oxidatively modified proteins in AD^200,201^. CKB is involved in maintaining local ATP reserves, while GS contributes to extracellular glutamate homeostasis and glutathione production. Disturbance of this key function of astrocytes may affect neurotransmission and drive excitotoxicity. Modification of CKB is associated with β-amyloid depositions and a loss of function of APOE as a scavenger for lipid peroxidation-derived aldehydes^202^ as both APOE deletion^203^ and high levels of lipid peroxidation products are associated with increased lipoxidative modifications of CKB, GS, vimentin and GFAP^204^.

#### Parkinson’s disease

Astrocyte pathology in Parkinson’s disease (PD) is mainly manifested by functional deficiency and loss of neuroprotection; all in all, reactive astrogliosis is limited and most likely secondary, induced primarily by neuronal death^205,206^. In some familial forms of PD, astrocytic atrophy was detected. In particular, in PD associated with *PRKN* mutation, a significant decrease in GFAP expression was found in both human post-mortem samples and organoids^207^. Similarly, astrocytes derived from induced pluripotent stem cells reprogrammed from PD patients with LRRK2 mutation showed morphological atrophy^208^. A decrease in astrocytic complexity was also observed in the late-stage post-mortem PD brains^209^. Pathological astrocytes in the context of PD have deficient mitochondrial function^208^ and decreased transmitophagy, the latter being instrumental for supporting energy production in dopaminergic neurons^210^. Protoplasmic astrocytes may accumulate and remove α-synuclein, thus exercising neuroprotection^211^, although overload with α-synuclein may cause mitochondrial damage while aggregated α-synuclein may spread through astrocytic syncytia^212,213^.

#### Huntington’s disease

HD is a monogenetic neurodegenerative disorder caused by a single dominant allele of the huntingtin gene containing an expanded number of CAG (cytosine, adenine, guanine) repeats; the disease develops when the number of repeats exceeds 40 (refs. ^214,215^). Astrocytes derived from human embryonic stem cells obtained from mutant Huntingtin (mHTT) embryos and grafted into mouse corpus callosum demonstrated aberrant differentiation and atrophy^216^. Morphological atrophy and retraction of astrocytic processes from cortico-striatal projection synapses (the first to be affected in HD) have also been reported in a HD mouse model^217^. In HD, astrocytes undergo functional asthenia manifested in the deficient K^+^ buffering and glutamate clearance. The transcriptome of HD mouse model striatal astrocytes reveal significant downregulation of K_ir_4.1, glutamate transporters and molecules of Ca^2+^ signaling. These astrocytes have a depolarized resting membrane potential and higher input resistance, reflecting a decrease in their size^150^. A decrease in the expression of K_ir_4.1 in striatal astrocytes was also verified in human HD samples^151,218^. Likewise, the expression of EAAT2 is significantly downregulated in human HD tissue and in mouse HD models^152,153^; HD astrocytes also produce less glutamine, thus affecting glutamate and GABA neuronal pools^219^. Specific expression of mutated Huntingtin with 160 CAG repeats in astrocytes resulted in a decrease in the expression of EAAT2 and in the emergence of a HD phenotype^220^. At the same time, astrocyte-specific ablation of mutant Huntingtin in mice that constitutively express this protein in all cells mitigated disease symptoms and slowed disease progression^221^, thus highlighting astrocytic contribution to the pathogenesis of HD.

#### Frontotemporal dementia—clustering of pathological astrocytes

Frontotemporal dementia (FTD) is the second most prevalent cause of early-onset dementia and is characterized by profound region-specific neurodegeneration. Anterior brain regions (for example, frontal and temporal lobes) are severely damaged, whereas posterior brain regions (for example, occipital lobe) are seemingly unaffected. FTD can occur sporadically (late-onset) or due to genetic mutations. About one-third of the genetic FTD cases are caused by an autosomal-dominant genetic mutation in either progranulin (GRN), microtubule-associated protein tau (MAPT) or chromosome 9 open reading frame 72 (C9orf72)^222–224^.

Astrocytes, arguably, are among the most affected cells in the brains of patients with FTD-GRN^225,226^. Pathological astrocytes are distinguished by the expression of a specific marker WD Repeat Domain 49 (WDR49), while immunohistochemistry demonstrated that WDR49-positive astrocytes form clusters that are scattered randomly throughout the cortex of patients with FTD^225,226^. These WDR49-positive astrocytes were exclusively found in brain regions with neurodegeneration (frontal and temporal cortex), and not in the occipital cortex of patients with FTD, indicating that these cells are topically related to neuronal loss. A follow-up study investigated the distribution of WDR49-positive astrocytes in other subtypes of FTD and in AD, and showed that WDR49-positive astrocytes are most abundant in FTD-GRN, have a different morphology in FTD with TDP43 versus TAU pathology, and colocalize with senile plaques in AD^226^.

At the same time, evidence for astrogliosis in mouse models for FTD is variable and probably reflects underlying genetic mutations^227^. In *Grn*^−/−^ mice, but not *Grn*^+/−^ mice, increased GFAP immunoreactivity was observed in the thalamus, hippocampus, cortex and amygdala^228^. Conversely, no increase in GFAP immunoreactivity was identified in *C9orf72*^−/−^ mice^229^. The brains from (G4C2)66 mice, another model for C9-FTD/ALS, showed signs of astrogliosis in the cortex^230^. In vitro, astrocytes derived from induced pluripotent stem cells obtained from patients with FTD-MAPT showed hypertrophy, elevated GFAP protein levels, disease-associated changes in TAU expression, increased vulnerability to oxidative stress, and protein ubiquitination^231^. The presence of GFAP was detected in the cerebrospinal fluid (CSF) and plasma from symptomatic FTD-GRN, and this correlated with age^227,232^. In the presymptomatic period, higher GFAP plasma concentrations correlated with a lower cognitive score and lower brain volumes, suggesting that GFAP expression is increased in the late presymptomatic period^227^.

#### Amyotrophic lateral sclerosis

Functional insufficiency and pathological remodeling of astrocytes play a central role in driving motor neuron death in ALS. Several pathological phenotypes including reactive, degenerating, aberrant and atrophic astrocytes are present in ALS^156,233–236^. Astrocytes derived from ALS post-mortem tissues trigger the death of neurons in co-cultures^237,238^, whereas transplantation of astrocytes derived from pluripotent stem cells obtained from patients with ALS into the spinal cord of mice resulted in the degeneration of motor neurons and motor deficits^239^. Using the human ALS-linked superoxide dismutase 1 (SOD1^G93A^) mutant gene to replicate ALS in mice further highlighted the primary role of astrocyte: astrocyte-selective silencing of SOD1 alleviated ALS symptoms and increased life span^240,241^, whereas restricted expression of SOD1 in neurons did not cause pathology^242,243^. Moreover, grafting fetal astrocytes bearing SOD1G93A to the spinal cord of healthy mice precipitated ALS-like pathology^244^, while transplanting healthy astrocytes slowed ALS progression in rats carrying mutant SOD1^G93A^ (ref. ^245^). The primary damaging effect is associated with a profound loss of astrocytic glutamate clearance, leading to excitotoxicity^246,247^. In patients with ALS, the expression of EAAT2 is decreased by up to 90% (refs. ^149,248^), and a similar decrease was observed in SOD1^G93A^ mice^148,249,250^. Aberrantly proliferating astrocytes that express both astrocytic and microglial markers exhibit toxicity in vitro. They display low levels of intermediate filaments, numerous microtubules, abundant secretory vesicles and lipid droplets, and lack homeostatic functions. Neurotoxicity of these cells can be mediated by the overproduction of ROS^236,251,252^.

#### Astrocytotauopathies

Astrotauopathies, characterized by tau accumulation predominantly in astrocytes, are classified, according to histopathology, into (1) astrocytic plaques, (2) tufted astrocytes, (3) ramified astrocytes, (4) globular astroglial inclusions, (5) thorn-shaped astrocytes and (6) granular/fuzzy astrocytes^253^. Astrocytic plaques, which appear in the form of fuzzy short argyrophilic processes arranged annularly with fine collaterals at vertical or sharp angles, are the hallmark of corticobasal degeneration, a primary tauopathy^253,254^. Tufted astrocytes identified by phosphorylated-tau immunoreactivity in the proximal segments of astrocytic processes are a histopathological hallmark of progressive supranuclear palsy, another rare primary tauopathy. Ramified atrophic astrocytes distinguish Pick’s disease, also known as frontotemporal lobular degeneration^255^, while globular astroglial tau inclusions are specific for FTD^256^. Finally, thorn-shaped and granular-fuzzy astrocytes are found in aging-related tau astrogliopathy, ARTAG^257^.

### Autoimmune astrocytopathies

Encephalopathies associated with the generation of antiself antibodies against two astrocyte-specific proteins GFAP and vimentin were discovered quite recently. In 1996, encephalopathy triggered by GFAP autoantibodies (GFAP-A) was described^258^. The presence of anti-GFAP antibodies in serum and cerebrospinal fluid is a critical diagnostic criterion, as clinical manifestations are nonspecific and can include movement disturbances, paresthesia, visual impairment, brainstem syndrome and autonomic dysfunction^259–262^. The clinical manifestations of GFAP-A are highly diverse and include encephalitis, myelitis and meningitis^262^. The second autoimmune astrocytopathy caused by anti-vimentin autoantibodies was described in 2025^263^. It has a clinical picture of unidentified meningoencephalitis with the involvement of several brain regions and lesions in bilateral corticospinal tracts. The CSF of patients stained astrocytes and ependymoglia in the rodent brains, again highlighting pathophysiological contribution of astroglial cells. The cellular pathophysiology of these diseases is unknown.

### Neuropathic pain

While neuronal hyperexcitability has traditionally been the focus of neuropathic pain research, the past two decades have seen growing interest in the role of astrocytes. In rodent models of neuropathic pain, reactive astrogliosis was well characterized in the spinal dorsal horn. Expression of GFAP is markedly upregulated after nerve injury and remains elevated for several weeks to months^264,265^. Reactive astrocytes contribute to pain hypersensitivity through activation of the JAK–STAT3 pathway^266^ with consequent release of cytokines, including tumor necrosis factor (TNF), interleukin (IL)-1β (IL-1β) and C–C motif chemokine 2 (CCL2)^267–270^. Reactive astrocytes also contribute to neuropathic pain through MAO-B-mediated GABA synthesis, as described in the following sections.

### Psychiatric disorders

#### Mood disorders and stress-induced depression

Astroglial pathology in major depressive disorders in human patients and stress-induced depressive behaviors in animals is represented by a significant decrease in the numbers and complexity of astrocytes in several brain regions, including, in particular, the prefrontal cortex, which is responsible for stress processing^144,146,271,272^. Other brain regions are affected, too; in the hippocampus, for example, reduced levels of glutamate transporters were found in animal models with depressive-like behavior^273–276^. In the hypothalamus, early-life stress resulted in a marked atrophy of astrocytes, aberrant purinergic signaling and limited supply of L-lactate, leading to a pathological activity of orexin neurons driving aberrant behaviors^277^. A decrease in astrocytic presence in the brain reduces homeostatic support of nervous tissue, while decreased astrocytic synaptic coverage affects synaptic transmission and plasticity, which are translated into depressive symptoms and behaviors^143^. Reduced glutamate uptake^278^ and increased release of GABA^279^ also affect synaptic transmission and plasticity, arguably contributing to the pathophysiology of depression. Astrocytic atrophy and asthenia are causal for depression: selective ablation of astrocytes or downregulation of astrocytic homeostatic molecules such as glutamate transporters or connexins is sufficient to trigger depressive-like behaviors in experimental animals^280–284^. Glutamate homeostasis, supported by astrocytes, is of particular relevance; aberrant glutamate clearance retunes synaptic transmission^77^, promotes dendritic shrinkage and is associated with depression in animals and humans^285–287^. Anti-depressant treatment or pharmacological stimulation of glial glutamate transporters alleviates aberrant behaviors and restores astrocytic morphology^146,284,288^. Thus, depression can be considered a primary astrocytopathy. The emergence of depressive symptoms and behaviors is, however, highly individual, and the same amount of stress produces different outcomes in both patients and animal models^289–292^. This resilience to stress is also linked to astrocytes. Manipulating the expression level of the astrocyte-specific plasma membrane–microfilament linker ezrin alters resilience to stress^147^. In bipolar disorder, the number of GFAP-positive astrocytic profiles decreases, accompanied by astrocytic atrophy^271,293,294^. Asthenic astrocytes may be linked to a hyperactive glutamatergic transmission (due to the reduced glutamate clearance observed in bipolar disorder^295,296^, reduced neuroprotection and metabolic abnormalities^297^.

#### Schizophrenia

Astrocytic pathology in schizophrenia manifests as altered astrocyte-specific protein expression, impaired differentiation and disrupted neuron–astrocyte interactions. Post-mortem analyses of the dorsolateral prefrontal cortex reveal elevated GFAP levels, particularly in individuals with psychotic symptoms, while other astrocytic markers remain unchanged, suggesting astrocyte-specific malfunction unrelated to antipsychotic treatment^298^. Developmental astrocyte abnormalities in humanized glial chimeric mice, transplanted with astrocytes from patients with schizophrenia, showed delayed differentiation and atrophic morphologies^299,300^. Gene expression studies highlight increased cortical astrocytic activity, coupled with parvalbumin interneuron loss, suggesting a role for astrocytes in neuronal network destabilization^301^. Malfunction of astrocyte-mediated GABAergic regulation is supported by findings in *GABRB2*-knockout mice, which exhibit astrocytic atrophy, degeneration of parvalbumin-expressing cortical interneurons, neuroinflammation and oxidative stress, aligning with key pathological features of schizophrenia^302^. Astrocytic differentiation deficits in schizophrenia are driven by upregulated inhibitors of the bone morphogenetic protein (BMP) pathway, which prevent proper maturation. Targeting BMP/SMAD4 and repressor element-1 silencing transcription factor (REST) pathways restored astrocyte function, presenting a potential therapeutic approach^303^. Astrocytes are targets of lithium antipsychotic treatment, with pharmacogenomic analyses identifying lipoxygenases LOX and peroxisome proliferator-activated receptor γ serving as regulators of astrocytic morphology and function, offering novel therapeutic directions^304^. Astrocyte–neuron interactions are crucial for synaptic regulation, with astrocytes enhancing synaptic gene expression and synaptic plasticity. These interactions are disrupted in schizophrenia, as reflected by dysregulation of synaptic adhesion genes and cholesterol metabolism, both being fundamental for synaptic integrity^305,306^. Thus, astrocytes are potential targets for schizophrenia therapy.

### Epilepsy and migraine

#### Epilepsy

Epilepsy and seizures reflect aberrant electrical activity in the brain. In epilepsy, astrocytes undergo a peculiar form of reactivity associated with an increased GFAP expression and atrophy of peripheral processes^307,308^. This atrophy is paralleled with asthenia: astrocytes in epileptic brains show significant downregulation of K_ir_4.1 channels, which affects K^+^ buffering and facilitates seizures^309^. Conditional knockout of K_ir_4.1 channels compromised K^+^ buffering, triggering seizures and an epileptic phenotype^310,311^. In humans, epilepsy is linked to several missense mutations, loss-of-function mutations or single-nucleotide polymorphisms in the *KCNJ10* gene encoding K_ir_4.1^309^.

Pathogenesis of epilepsy also included deficient glutamate clearance and insufficient inhibition; epileptic tissue has high glutamate content^312^ reflecting a 20–40% reduction in the expression of astrocytic glutamate transporters in the hippocampi of patients with temporal lobe epilepsy^313^. Likewise, downregulation of astrocytic glutamate transporters is confirmed in animal models of the disease^142,314,315^, whereas genetic deletion of transporters triggers seizures^313,316^. Epileptic astrocytes are also characterized by partial loss of glutamate synthetase and reduced GABA availability^317^, increased expression of astrocyte-specific adenosine kinase—which limits adenosine levels and further decreases adenosine A1 receptor-mediated inhibition^318^—loss of AQP4 polarization at astrocytic endfeet^319^ and downregulation of astrocytic Cx43; these changes collectively contribute to increased epileptiform activity^320,321^. A gain-of-function mutation in the anion conductance of EAAT1 in astrocytes is associated with spontaneous seizures^322^. This mutation reduces astrocytic Cl⁻ levels and trigger apoptosis^323^. Deletion of NKCC1 in astrocytes, which reduces astrocytic Cl⁻, leads to a lower seizure threshold^324^. Finally, the loss of gap junctional coupling among astrocytes lowers the seizure threshold by disturbing normal ionostasis^325^. Notably, astrocytic uncoupling has also been shown to directly affect inhibitory transmission^326^.

#### Migraine

Monogenic familial hemiplegic migraine (FHM) type 2 is a primary genetic astrocytopathy caused by loss-of-function mutations in the *ATP1A2* gene encoding the α2 subunit of Na^+^/K^+^ ATPase (the NKA) expressed solely in astrocytes^327^. FHM type 2 belongs to migraine with aura, which is associated with spreading depression^328^. The α2-containing NKA is the principal molecule of K^+^ buffering and is linked to regulation of expression of astrocytic glutamate transporters^329^. Astrocytes in FHM type 2-mutant expressing mouse demonstrated deficient K^+^ buffering and glutamate clearance, which also contribute to the initiation of spreading depression^329^.

### Primary astrocytic leukodystrophies

Alexander disease (AxD), the first known monogenic disease originating in astrocytes, is caused by toxic gain-of-function mutations in GFAP manifested by severe white matter damage^330,331^. While the molecular pathogenesis of AxD remains incompletely understood, massive reactive astrogliosis with Rosenthal fibers composed of aggregated GFAP, vimentin, nestin, αB-crystallin and small heatshock proteins together with prominent leukodystrophy are the pathological hallmarks, with the pathophysiological picture including mitochondrial malfunction, redox imbalance and susceptibility to oxidative stress^331–333^. Increased stress susceptibility and impaired differentiation of astrocytes and neurons was found in co-cultures and in neural organoids generated from AxD patient-derived induced pluripotent stem cells with AxD-causing GFAP^R239C^ mutation^334^.

Vanishing white matter disease is an autosomal recessive, polygenic disorder caused by mutations in five genes (*EIF2B1*, *EIF2B2*, *EIF2B3*, *EIF2B4* and *EIF2B5*) encoding the eukaryotic translation initiation factor eIF2B, a key control point for protein synthesis in all eukaryotes^335^. The pathophysiology of vanishing white matter disease is cell specific and primarily targeting astrocytes. Atrophic astrocytes with reduced complexity and blunt and coarse processes are present in the white matter and are characterized by impaired GFAP-related structures, suppressed expression of S100B, aberrant maturation and loss of astrocytic functions ultimately translated into white matter disintegration^336,337^.

Megalencephalic leukoencephalopathy with subcortical cysts (MLC), is another primary leukodystrophy caused by recessive pathogenesis variants in the genes *MLC1* (Modulator of VRAC Current 1) or *HEPACAM* (Hepatic and Glial Cell Adhesion Molecule, previously known as *GlialCAM*)^338^. In astrocytes, GlialCAM and MLC1 assemble into a functional unit, interacting with Na^+^–K^+^ ATPase, SLC ionic transporters, K_ir_4.1 channels, connexins, AQP-4 water channels, volume-regulated anion channels (VRAC) and transient receptor potential vanilloid 4 channel (TRPV4), and are also associated with the dystrophin–glycoprotein complex regulating endfeet channels and transporters^339–341^. In MLC, astrocytes show impaired K^+^ buffering, glutamate uptake and ionic homeostasis, which translate into white matter damage in a yet uncharacterized way^339,342,343^.

## Imaging human reactive astrocytes

**Fig. 6:**
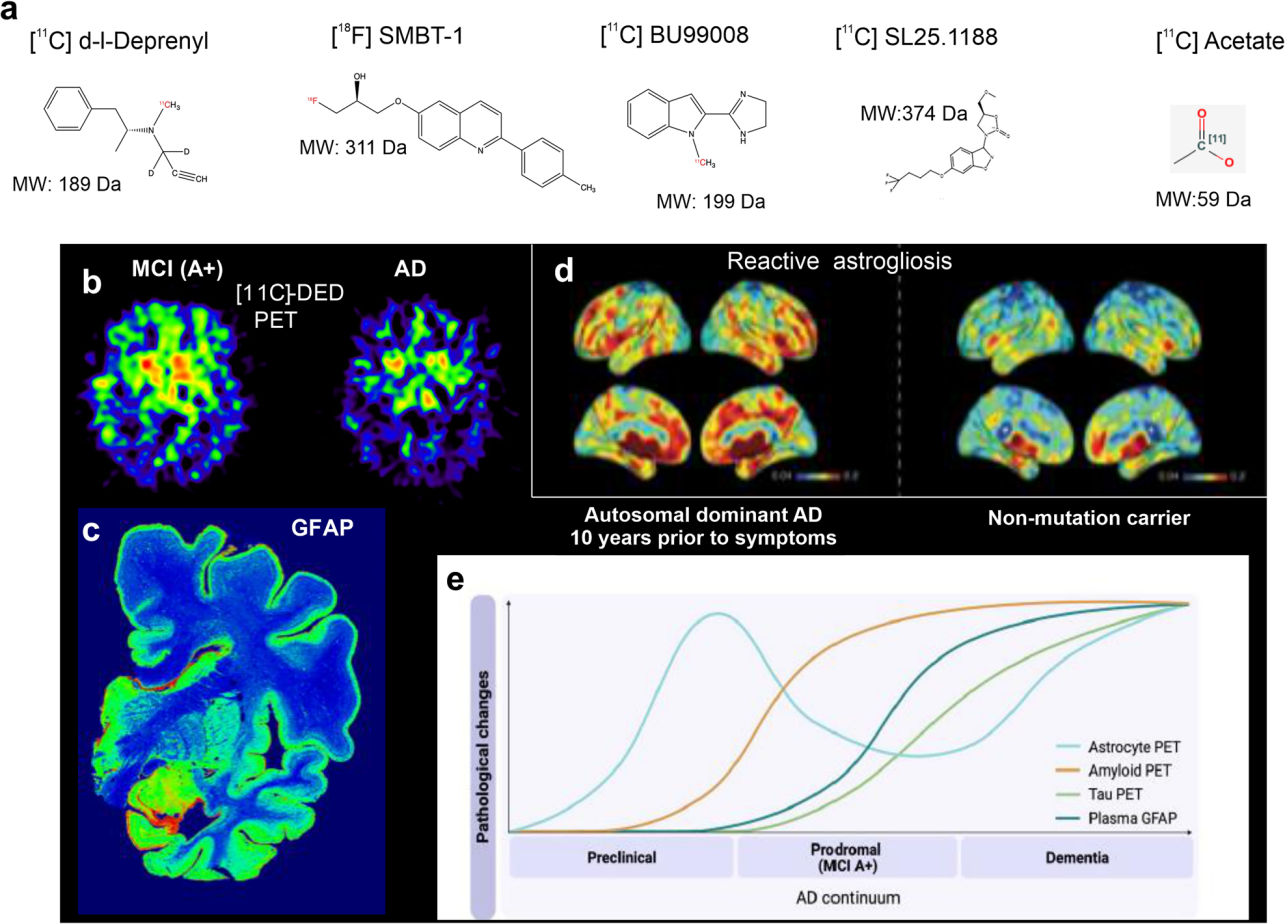
PET imaging of astrocytes in the human brain in AD. **a** Chemical structures of different PET tracers for visualizing astrogliosis in the human brain. **b** Higher [^11^C]deprenyl PET binding (astrogliosis) in a patient with MCI (amyloid positive; prodromal AD) compared with a patient with AD. Reproduced, with permission, from ref. ^353^. **c** GFAP antibody staining of astrocytes in hemispheric section of AD brain showing high astrogliosis in hippocampus/entorhinal cortex. **d** High [^11^C]deprenyl PET binding (astrogliosis) in an APPswe mutation carrier 10 years before expected clinical symptoms compared with a nonmutation carrier (photo courtesy of Nordberg Translational Imaging Lab). **e** Reactive astrogliosis in the AD disease continuum. The light-blue curve illustrates increased [^11^C] deprenyl PET binding in preclinical AD as the first wave of reactive astrogliosis. When plaque deposition (orange curve) increases, the astrocyte PET curve declines. GFAP levels in plasma increase (teal curve) with β-amyloid deposition in the brain. This is followed by tau pathology (green curve) and a second wave of reactive astrogliosis, indicative of the later stages of AD. Reproduced, with permission, from ref. ^360^.

The rapid development of in vivo molecular imaging techniques such as positron emission tomography (PET) has made it possible to visualize astrocytes in the brain of living humans and to study changes in astrogliosis in brain disorders. Several astrocyte PET tracers currently in clinical and research use include [11C]deprenyl, [^11^C] BU99008, [^18^F] SMBT-1, [^11^C] 25.1188 and [^11^C] acetate (Table 2 and Fig. 6) and are described in more detail below. However, developing methods for in vivo visualization of astroglia is challenging, as reactive astrocytes undergo remodeling into diverse states with varying functions and properties^118^. We may therefore expect multiple subtypes of astrocytes in different diseases and stages of different disorders.

**Table 2:** PET imaging of astrocytes in disease context.

PET radiotracer	Molecular target	Disease	Reference
[^11^C] **deprenyl**	MAO-B	Epilepsy	^349^
		ALS	^350^
		JCD	^352^
		MCI	^353^
		AD	^353^
		AD	^357^
		AD	^356^
		AD	^358^
**[**^**18**^**F] SMBT-1**	MAO-B	AD	^366^
		AD	^367^
**[**^**11**^**C] BU99008**	I_2_BS	AD	^363^
		AD	^364^
		PD	^365^
		PD	^672^
**[**^**11**^**C] SL25.1188**	MAO-B	Depression	^368^
		Psychosis	^369^
		Post-traumatic stress disorder	^370^
		Traumatic brain injury	^371^
		COVID-19	^372^
**[**^**11**^**C] acetate**	Acetate	AD	^373^
		MS	^374^

### [^11^C] deprenyl PET labeling astrocytic MAO-B to visualize astrogliosis

MAO-B is a mitochondrial enzyme responsible for the oxidative deamination of monoamines, including dopamine, norepinephrine and phenylethylamine^344^. Although traditionally associated with neuronal neurotransmitter metabolism, MAO-B is expressed predominantly (if not exclusively) in astrocytes, where it plays a central role in reactive astrogliosis and neuroinflammation^345,346^. In the astrocytes, MAO-B is localized to the outer mitochondrial membrane and contributes to metabolic regulation and oxidative stress responses^347^. MAO-B is highly expressed in brain regions susceptible to neurodegenerative pathology, including the striatum, hippocampus and cortex^348^. Increasing evidence suggests an upregulation of MAO-B in reactive astrocytes in neuroinflammatory pathologies as well as in many chronic neurological disorders^345^; this has motivated efforts to measure MAO-B as a marker of astrogliosis and prompted the development of the first PET tracer, [^11^C] d-l-deprenyl -l-dL deprenyl, as a selective target for imaging reactive astrocytes.

### [^11^C] deprenyl PET

Deprenyl is an irreversible MAO-B inhibitor with a well-established use in the treatment of patients with PD. The concentration of the ligand used in PET studies is, however, 100–1,000 times lower than its pharmacological dose. In the initial studies in patients with epilepsy, [^11^C] d-l-deprenyl PET demonstrated a lower uptake in the temporal lobe compared with healthy controls and turned out to be a useful method to diagnose temporal lobe epilepsy^349^. An increased astrogliosis measured as an enhanced uptake of [^11^C] d-l-deprenyl was observed in the pons and white matter of patients with ALS^350^. When patients with Jakob–Creutzfeldt disease underwent PET imaging with [^11^C] d-l-deprenyl and [^18^F]fluorodeoxyglucose ([^18^F]FDG), increased astrogliosis and decreased cerebral glucose metabolism were observed in the frontal, occipital and parietal cortices and cerebellum^351,352^. Autopsy studies confirmed a good correlation between the in vivo measured high [^11^C] d-l-deprenyl binding with high reactive astrogliosis measured by GFAP immunostaining at autopsy.

Hypertrophic reactive astrocytes surrounding senile plaques can be seen in autopsy tissue of patients with AD; hence, it was logical to perform [^11^C] d-l-deprenyl as well as amyloid [^11^C]-labeled Pittsburgh Compound-B ([^11^C] PIB) PET in patients with AD and mild cognitive impairment (MCI)^353^. It was quite unexpected that patients with MCI with pathological β-amyloid load revealed by PIB positive binding demonstrated higher [^11^C] d-l-deprenyl uptake, indicating more prominent astrogliosis compared with patients with AD as well as with MCI amyloid-negative patients and age-matched healthy controls^353^. All individuals also underwent [^18^F] FDG PET, which showed pronounced hypometabolism in patients with AD compared with the patients with β-amyloid positive MCI. These observations created the initial hypothesis of prominent astrogliosis as an early event in the time course of pathology in the AD continuum^354^. This initial observation was followed by multitracer PET imaging with [^11^C] d-l-deprenyl, [^11^C] PIB and [^18^F] FDG studies in members of families with known autosomal-dominant familial AD in both presymptomatic and symptomatic carriers as well as in patients with sporadic AD and MCI and healthy individuals^355^. An inverse relationship was observed in the brain of autosomal-dominant familial AD mutation carriers between [^11^C] d-l-deprenyl and [^11^C] PIB PET demonstrating a high uptake of [^11^C] d-l-deprenyl PET (that is, prominent reactive astrogliosis) already 16–20 years before the onset of clinical symptoms; the [^11^C] d-l-deprenyl signal declined with increasing β-amyloid PIB binding in the brain. The [^11^C] d-l-deprenyl uptake nevertheless was high compared with non-mutation carriers at the onset of first cognitive symptoms and showed a negative correlation with [^18^F] FDG uptake^356–358^. Based upon these observations, a ‘two-wave model of reactive astrogliosis’ in the AD continuum was suggested^359,360^.

### [^11^C] BU99008 PET

[^11^C] BU99008 was developed as a PET tracer with high affinity and reversible binding to imidazol I_2_BS localized in the outer mitochondrial membranes and expressed mainly in astrocytes, with lower neuronal presence^361^. [^11^C] BU99008 is considered as a PET ligand with good penetration to the brain and reversible and highly specific binding to I_2_BS^362^. A high uptake of [^11^C] BU99008 was observed in cortical brain regions of patients with AD and those with β-amyloid positive MCI compared with healthy controls, showing a positive correlation with β-amyloid load^363^. Uptake of [^11^C] BU99008 in the temporal and parietal cortex in β-amyloid-positive patients positively correlated with [^18^F] FDG uptake and gray matter volume, while negatively correlating with β-amyloid [^18^F] florbetaben uptake^364^. These findings were interpreted as showing reactive astrogliosis at the early stages of AD, whith subsequent astrocytes atrophy and loss of reactivity with higher β-amyloid deposition^364^. During the early stages of PD, [11C]BU99008 PET showed an increased uptake in frontal, temporal, parietal, occipital and insula cortices and subcortical regions such as caudate, putamen, thalamus and brainstem as compared with healthy controls, whereas in advanced stages these differences disappeared^365^. Similarly, no differences were observed in [^11^C] BU99008 uptake in patients with PD and healthy controls^365^, again indicating that astrogliosis occurs mainly in early stages of the disease.

### [^18^F] SMBT-1 PET

[^18^F] SMBT-1 is a reversible MAO-B inhibitor developed through lead optimization from the THK-5351 Tau PET tracer^366^. In patients with AD, a significant increase in the uptake of [^18^F] SMBT-1 was found compared with β-amyloid-negative controls^367^. In addition, a positive correlation was observed in patients with AD between astrogliosis measured by [^18^F] SMBT-1 and amyloid PET uptake in the brain^367^.

### [^11^C] SL25.1188

[^11^C] SL25.1188 is a PET tracer based on a reversible MAO-B inhibitor used in psychiatric disorders such as depression^368^, psychosis^369^ and post-traumatic stress disorder^370^, in traumatic brain injury^371^ and in COVID-19 with chronic depressive and cognitive symptoms^372^. In PET imaging of patients with traumatic brain injury, an increased [^11^C] SL25.1188 uptake was observed in the prefrontal and cortical brain regions compared with controls, and this increase showed an inverse correlation with performance on the Trail Making Test, reflecting reduced psychomotor and processing speed^371^. In patients with COVID-19 uptake of the [^11^C] SL25.1188 was higher in several brain regions, including the prefrontal cortex, anterior cingulate cortex, hippocampus, dorsal putamen and ventral striatum, compared with healthy control participants, thus revealing astrogliosis as pathogenetic factor^372^. In patients with psychosis or post-traumatic stress disorder, a slight tendency toward a lower [^11^C] SL25.1188 uptake compared with controls was noted^369,370^.

### [^11^C] acetate

Acetate is a substrate for energy generation in astrocytes associated with their metabolism and activity. A significantly higher [^11^C] acetate uptake was observed in several brain regions, including the entorhinal cortex, hippocampus and temporal cortex in patients with AD compared with controls^373^. Increased [^11^C] acetate uptake was paralleled by a significant decrease in [^18^F] FDG uptake in the same brain regions^373^. A negative correlation was also observed between cognitive mini mental test score (MMSE) score and [^11^C] acetate uptake^373^. In patients with MS, high uptake of [^11^C] acetate was observed in both gray and white matter, which correlated with the number of magnetic resonance imaging (MRI) lesions^374^. Increased uptake of acetate observed in the patients is probably driven primarily by reactive astrocytes, which upregulate monocarboxylate transporter 1 (MCT1) both in vivo in the brains of AD model mice and patients, as well as in vitro^373^. Astrocyte-specific suppression of MCT1 expression significantly reduced the [^11^C] acetate uptake.

To conclude, multiple astrocyte PET tracers indicate reactive astrogliosis in several brain diseases. Most of the PET studies (Table 1) focused on patients with AD at different stages of the disease continuum. When comparing three astrocytic PET tracers ([^11^C] d-l-deprenyl, [^18^F] SMBT-1 and [^11^C] BU99008), some differences in the sensitivity to pick up changes in astrogliosis during the development of AD were noted. In vitro binding studies using the three PET ligands in post-mortem tissues also revealed differences in binding properties, including multiple binding sites with varying affinities^375,376^. Hitherto, [^11^C] d-l-deprenyl is the only astrocytic tracer that demonstrated a high uptake in the presymptomatic carrier of AD 15–20 years before the onset of cognitive symptoms, that is, at the stage when the β-amyloid is rather low. It is therefore possible that soluble oligomeric amyloid triggers the reactive astrogliosis detected by [^11^C] d-l-deprenyl. An inverse correlation was observed between reactive astrogliosis measured by [^11^C] d-l-deprenyl and β-amyloid load measured by [^11^C] PIB PET imaging during the progression of the disease until the onset of clinical symptoms. This highlights the first wave of reactive astrogliosis in AD preceding other pathological markers such as tau deposits, neurodegeneration and cognition. A second wave can also be observed in the later stage of AD with a positive correlation with amyloid load^375,376^. The second wave was also demonstrated with [^18^F] SMBT-1 PET showing a positive correlation with β-amyloid PET in patients with AD^367^. The use of several astrocyte PET tracers might increase the possibility of detecting multiple astrocyte states and lead to the development of subtype-specific astrocytic PET tracers.

## Targeting astrocytic molecules and cascades

In this section, we provide an overview of major molecules and molecular cascades expressed predominantly or exclusively in astrocytes. We also narrate the current state of knowledge on the potential drugs that target these molecular entities, representing astrocyte-specific therapeutic strategies (Table 3).

**Table 3:** Therapeutic targeting of astrocytic molecules and pathways.

Molecular target	Modifying agents/strategies/drugs	References
GFAP	Zilganersen (GFAP-targeted antisense nucleotides); clinical study; Zilganersen is an FDA-approved fast-track drug for AxD	https://clinicaltrials.gov/study/NCT04849741
Ezrin	Physical exercise and enriched environment? Acupuncture?	^146^
EAAT2 glutamate transporter	Animal experiments: (ALS, AD models): Ceftriaxone (β-lactame antibiotic) LDN-212320 (pyridazine derivative). Clinical practice: Riluzole	^440–442,444,447,448^
Complement system	Animal experiments (stroke): intranasal C3a complement fragment delivery	^383^
Connexin 43	Clinical trials (skin wound) Antisense oligodeoxynucleotide Animal experiments (spinal cord injury): fiber-hydrogel scaffold-mediated Cx43-AsODN Animal experiments (neuropathic pain, stroke): Cx43 hemichannel inhibitors Peptide5, Gap26 and Gap27 Animal experiments (epilepsy): BBB-permeable Cx43 hemichannel inhibitors TAT–Gap19 and TAT–Cx43_266–283_ Animal experiments (AD model): BBB-permeable Cx43 hemichannel inhibitor TAT–Cx43@LNP with increased retention in the CNS	^480,496,498,506,507,509,510^ ^516,518^ ^517^
Monoamine oxidase-B (MAO-B)	Irreversible inhibitors: selegiline and rasagiline Reversible inhibitors: safinamide and KDS2010 Preclinical studies showed positive effects of KDS2010 on AD- and PD-like pathology	^531,542^
Ornithine decarboxylase 1 (ODC1); part of the urea cycle	DFMO led to a removal of β-amyloid and cognitive impairment in preclinical studies	^547^
Aquaporin 4	Animal experiments: (spinal cord trauma) trifluoperazine; clinical trial: AER-271	^574^

### GFAP—targeting reactive astrogliosis

GFAP is the main component of astrocyte cytoplasmic intermediate filaments (also referred to as nanofilaments) (Fig. 2C). These nanofilaments are dynamic structures acting as a signaling system regulating cellular stress responses in health and disease^332,377^. The upregulation of GFAP and vimentin are hallmarks of astrocyte reactivity and can be seen in neurotrauma, stroke or neurodegenerative diseases, such as AD or ALS^332,378^, while GFAP mutations result in AxD. Treatment with *GFAP*-targeted antisense nucleotides mitigates white matter loss and motor impairment, in both mouse^379^ and rat^380^ models of AxD. The same approach is being applied to AxD patients in an ongoing phase 1–3 clinical study (Zilganersen, NCT04849741; the drug received FDA fast-track designation; https://ionistrials.com/study/a-study-to-evaluate-the-safety-and-efficacy-of-ion373-in-patients-with-alexander-disease-axd/).

Genetic ablation of *GFAP* affects reactive astrogliosis. The reactivity of astrocytes in *GFAP*^−/−^ mice is attenuated after neurotrauma^381^, whereas *GFAP*^−/−^ astrocytes show decreased motility^382^. Using cellular deconvolution of bulk transcriptomics data, GFAP was identified as the main driver of reactive astrogliosis in the peri-infarct region after ischemic stroke^383^. In *GFAP*^−/−^ mice, vimentin can compensate for the absence of GFAP^384,385^, thus masking some phenotypes, which become apparent only when *GFAP*^−/−^ mice were crossed with a *Vim*^−/−^ background. Astrocytes of *GFAP*^−/−^*Vim*^−/−^ mice completely lack cytoplasmic nanofilaments, and these mice have provided insights into the role reactive gliosis in the brain, spinal cord and retina in a wide range of neurological diseases^332,377,386^. The abundance and domain tiling of astrocytes in the CNS of *GFAP*^−/−^*Vim*^−/−^ mice is normal. However, following injury, *GFAP*^−/−^*Vim*^−/−^ astrocytes do not undergo the characteristic hypertrophy of their main cellular processes^381^ and show a number of molecular features of attenuated reactive gliosis, such as decreased activation of c-FOS and ERK^387^ as well as less prominent upregulation of the 14-3-3 adapter proteins^388^. *GFAP*^−/−^*Vim*^−/−^ Müller cells fail to respond to retinal ischemia by increasing the stiffness of their endfeet and inner processes^389^. *GFAP*^−/−^*Vim*^−/−^ mice have reduced Notch signaling from astrocytes to neural stem/progenitor cells that control neurogenesis in adult mammalian CNS^390,391^, and *GFAP*^−/−^*Vim*^−^^/−^ mice were instrumental for the finding that vesicle trafficking in astrocytes depends on vimentin, GFAP and also nestin, another nanofilament protein, which is present in immature as well as reactive astrocytes^392–396^, but also in some astrocytes in the dentate gyrus of the hippocampus^396^.

*GFAP*^−/−^*Vim*^−/−^ mice have been essential for defining the concept of context-specific roles of reactive astrocytes and characterizing the disease-specific functions of these cells. Depending on the specific context, reactive gliosis can be either beneficial—promoting improved functional outcomes—or detrimental, leading to maladaptive impairments or neurodegeneration^377^.

Genetic attenuation of reactive gliosis in *GFAP*^−/−^*Vim*^−/−^ mice has a number of negative consequences. It decreases the resistance of the CNS to severe mechanical stress^397,398^ and to ischemic injury^399–402^. Ischemic stroke induced in *GFAP*^−/−^*Vim*^−/−^ mice by middle cerebral artery transection leads to a more prominent loss of the ischemic penumbra, with less efficient endothelin-3-induced blockage of gap junction communication between astrocytes and decreased glutamate clearance^401^. Astrocytes in *GFAP*^−/−^*Vim*^−/−^ mice had lower glutamine levels^403^ and were less resilient when challenged with oxidative stress^399^. Thus, GFAP and vimentin nanofilaments support neuroprotective functions of astrocytes in acute ischemic stroke and exemplify the positive role of reactive astrocytes. This contention is further supported by the finding that, after retinal ischemia–reperfusion, fewer cells of the inner retina of *GFAP*^−/−^*Vim*^−/−^ mice survived^402^. The effect of reactive astrogliosis after acute injury depends on the presence of the ischemic penumbra and differs between the immature and adult CNS. The absence of vimentin and GFAP does not affect tissue loss in photothrombotic brain injury—which involves a very limited ischemic penumbra^404^—nor in neonatal hypoxic-ischemic brain injury, a model of birth asphyxia^405^. The formation of post-traumatic perilesional astrocytic border is impaired in *GFAP*^−/−^*Vim*^−/−^ mice and is accompanied by slower wound healing after both brain and spinal cord trauma^381,403^. In addition, *GFAP*^−/−^*Vim*^−/−^ mice show an altered response of astrocytes in neurodegenerative diseases, including limited presence of astrocyte processes in the direct vicinity of β-amyloid plaques, impaired cytokine production, aberrant astrocyte–microglia interactions and faster progression of both AD and Batten disease in the respective mouse models^127,197,406^. Regeneration of crushed sciatic nerve takes longer in *GFAP*^−/−^*Vim*^−/−^ mice; however, the final outcome is comparable to that of wild-type mice^407^. The slower wound healing after brain or spinal cord trauma in *GFAP*^−/−^*Vim*^−/−^ mice^381,403^, may ultimately lead to a better functional outcome, or contribute to neurodegeneration, malfunction and disability^377^. Perilesional astrocytic border contributes to post-traumatic functional recovery^9,161,408^. Attenuation of reactive astrogliosis also affects post-ischemic neural plasticity responses: corticospinal tract remodeling and axonal regeneration following photothrombotic stroke are delayed in *GFAP*^−/−^*Vim*^−/−^ mice^404^. The *GFAP*^−/−^*Vim*^−/−^ mice exhibit maladaptive post-stroke neuronal connectivity, characterized by increased synaptic plasticity in the perilesional region and an altered balance between lost and newly generated neuronal connections in sensorimotor networks, resulting in impaired recovery of sensorimotor functions^409^.

Genetic attenuation of reactive gliosis in *GFAP*^−/−^*Vim*^−/−^ mice also has a number of positive consequences. Pathological and detrimental neovascularization in oxygen-induced retinopathy is mitigated in *GFAP*^−/−^*Vim*^−/−^ mice^397^, whereas photoreceptor cell death and monocyte infiltration are both reduced in *GFAP*^−/−^*Vim*^−/−^ mice with sodium hyaluronate-induced degeneration of retina^387^. In addition, *GFAP*^−/−^*Vim*^−/−^ mice exhibit increased neurogenesis in the dentate gyrus of the hippocampus in the absence of any challenge^391,410^ and increased memory extinction, conceivably due to the increased rate of the reorganization of the hippocampal circuitry^411^. The *GFAP*^−/−^*Vim*^−/−^ mice also show increased neurogenesis following hypoxic-ischemic injury to the immature brain^405^ and after hippocampal deafferentation in adults^391^, as well as improved regeneration of neuronal axons and synapses after trauma^381,412,413^. The CNS environment in *GFAP*^−/−^*Vim*^−/−^ mice is more supportive for integration and survival of neural grafts in the retina^414^ while supporting increased differentiation of transplanted neural stem cells into neurons and astrocytes in hippocampus^415^. These results suggest that limiting graft-induced reactive gliosis might be the way to increase the success of transplantation of neural grafts or neural progenitor cells in the brain, spinal cord or retina. It remains unclear to what extent the improved graft integration is a consequence of attenuated reactive gliosis or of an altered Notch signaling between resident astrocytes and neural grafts or grafted neural stem/progenitor cells. The appeal of treatment strategies that target and modulate reactive astrogliosis in a context-dependent manner is further supported by recent findings showing that pharmacological modulation of astrocyte reactivity improves neuronal connectivity in the peri-infarct cortex and accelerates motor function recovery after stroke^383^. Thus, understanding disease-specific astrocyte responses and targeting and modulating them presents an innovative treatment strategy for stroke and other neurological disorders.

### Ezrin—controlling astrocytic morphological plasticity

Astrocytic leaflets are highly dynamic; their association with synapses can change rapidly, thus affecting the volume of the synaptic cleft, as well as the efficacy of ion and neurotransmitter buffering and removal. These changes may facilitate or limit neurotransmitter spillover, thus contributing to synaptic plasticity^416^; retraction of astrocytic processes from synapses may cause aberrant transmission and plasticity^134^. Swift morphological remodeling of astrocytic leaflets requires plasmalemma–cytoskeleton interactions, mediated in particular by plasmalemmal linker ezrin. Ezrin belongs to the ezrin–radixin–moesin (ERM) family of proteins, which are involved in the assembly and stabilization of membrane–cytoskeletal complexes. This role is critical for controlling membrane dynamics that define cell shape and polarity, the formation of membranous protrusions, and the motility of cellular processes^417,418^. Ezrin in particular is enriched in microvilli of various types of epithelial cells from the placenta to renal ducts^419^; ezrin connects plasmalemma with F-actin, thus mediating morphological plasticity^420^. In the CNS, ezrin is highly expressed in microvilli of ependymal glia and in astrocytic leaflets^421^; in general, most of ezrin in the CNS is localized to astrocytes^422^. Experiments in vitro showed that ezrin regulates extension of astrocytic philopodia (analogs of leaflets), while in situ astrocytic ezrin expression follows circadian changes in glutamatergic transmission, thus changing synaptic coverage^423^. Various learning paradigms increase the expression of ezrin in hippocampal astrocytes, potentially enhancing synaptic coverage^424^. Depletion of astrocytic ezrin translated in shorter leaflets, decreased astrocytic synaptic coverage and glutamate spillover^425^. Ezrin also interacts with glutamate transporters and may regulate the efficacy of glutamate uptake^426,427^.

**Fig. 7:**
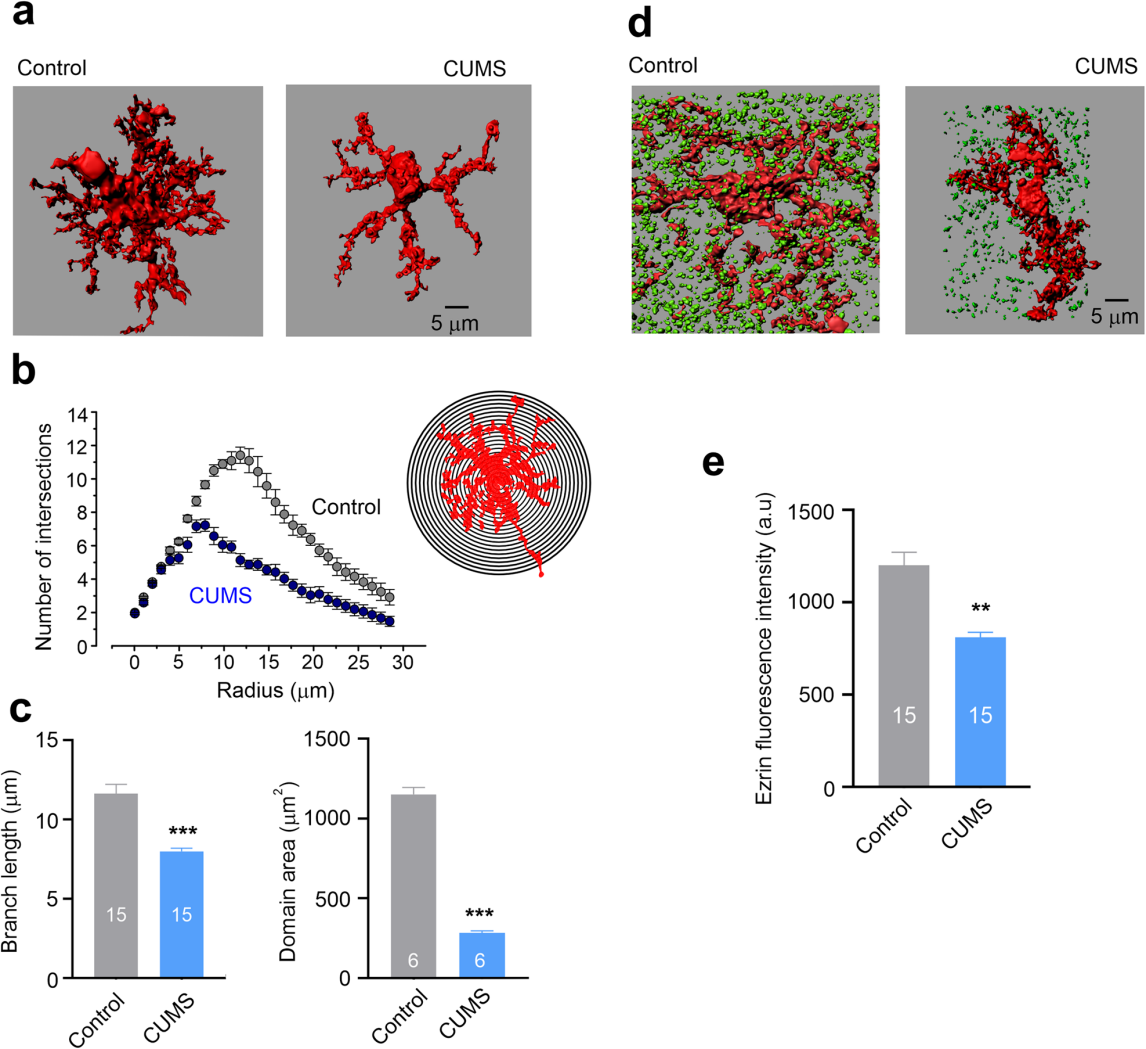
Ezrin and morphological atrophy of astrocytes in stress-induced depression. **a** Representative 3D reconstruction of astrocyte in control mice and mice subjected to chronic unpredictable stress (CUMS), which triggered depressive-like behaviors. **b** Sholl analysis of astrocytic morphology for control and CUMS groups shows the number of intersections of astrocytic branches with concentric spheres centered in the middle of cell soma. **c** Left: average length of astrocytic processes (branches). Right: average size of astrocytic domain areas. **d** Representative 3D reconstruction of astrocytic profiles (red; astrocytes were labeled with virally transfected with mCherry) with Ezrin puncta (green; immunostaining) for control and CUMS groups. **e** Average fluorescence intensity of ezrin associated with astrocytic profiles. All data are presented as mean ± s.e.m. *P < 0.05, **P < 0.01, ***P < 0.001. The number of experiments is indicated in each column. Reproduced from ref. ^146^.

In pathology, downregulation of ezrin expression is typically associated with—and probably contributes to—astrocytic atrophy, reduced number and volume of astrocytic leaflets, leaflet retraction and decreased synaptic coverage. Astrocytic atrophy, in turn, is linked to cognitive impairments. Astrocyte-specific ezrin knockout triggered cognitive impairments and anxiety-like behaviors^424^. Decreased ezrin expression coincides with astrocytic atrophy in aging in humans and mice^135,424^, with stress-induced depression^146^ (Fig. 7), with systemic inflammation and postoperative cognitive impairments^424^, and with general anesthesia-induced cognitive abnormalities^428^. Overexpression of ezrin in astrocytes results in an increased astrocytic morphological presence and improved cognition^424^. Manipulation with ezrin expression in astrocytes also indicated the resilience of mice to chronic stress: ezrin downregulation increased susceptibility to stress and facilitated emergence of depression-like behaviors, whereas overexpression of ezrin made mice resistant to chronic stress^147^. Treatment with antidepressants as well as acupuncture at a specific acupoint alleviated depression-induced behaviors in response to chronic stress, increased expression of ezrin and rescued astrocytic atrophy^146^. In summary, ezrin is an astrocyte-specific molecular target to manage cognitive disorders.

### Glutamate–glutamine cycle—preventing excitotoxicity

**Fig. 8:**
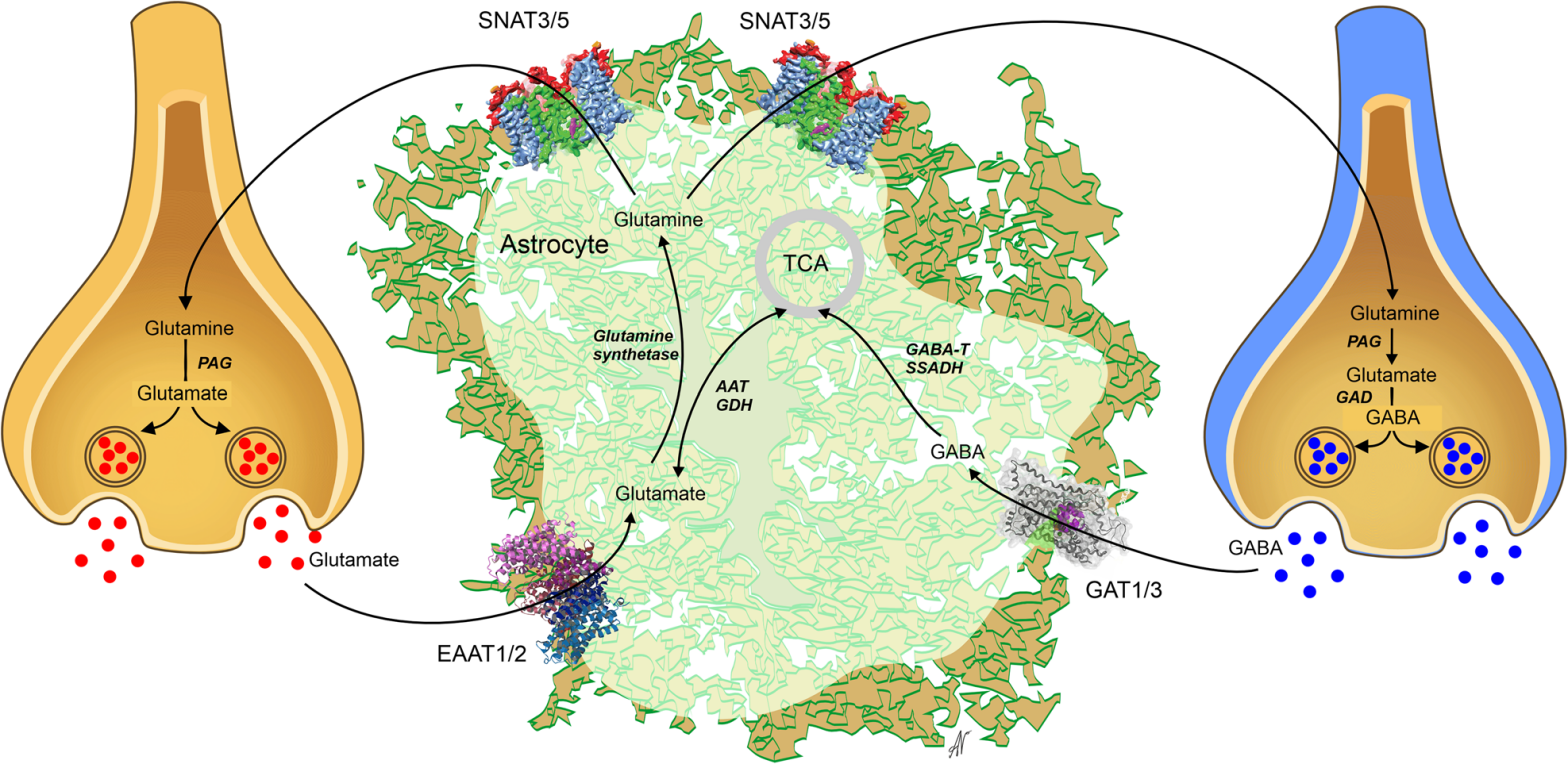
Neuronal–astrocytic glutamate (GABA)–glutamine shuttle. AAT, aspartate aminotransferase; EAAT1/2, excitatory amino acid transporters 1 and 2; GABA-T, GABA-α-ketoglutaric acid aminotransferase; GAD, glutamic acid decarboxylase; GAT1/3, GABA transporters 1 and 3; GDH, glutamate dehydrogenase; PAG, phosphate-activated glutaminase; SNAT3/5, sodium-dependent neutral amino acid transporters 3 and 5; SSADH, succinic semialdehyde dehydrogenase; TCA, tricarboxylic acid cycle. Reproduced with permission from ref. ^4^.

Astrocytes are indispensable for CNS synaptic transmission supplying neurons with glutamine, an obligatory precursor for glutamate and (by proxy) GABA as well as clearing glutamate and, to a lesser extent GABA, from the synaptic cleft and interstitium. The exchange of glutamate, glutamine, and GABA between neurons and astrocytes is mediated by the glutamate(GABA)–glutamine cycle, which is critical for maintaining excitatory and inhibitory neurotransmission and preventing excitotoxicity^27,73,429,430^. The cycle is composed of plasmalemmal glutamate transporters, GS, glutamate-synthesizing enzymes and glutamine transporters mediating the export (astrocytes) and import (neurons) of the latter (Fig. 8). Astrocytes remove ~80% of glutamate released during synaptic transmission by Na^+^-dependent EAAT1 and EAAT2 (SLC1A2 and SLC1A3); EAAT1 is mainly expressed in the cerebellum, retina and circumventricular organs, whereas EAAT2 predominates in all other brain regions. Glutamate transporters are mainly localized to perisynaptic astrocytic leaflets at a high density reaching 8,500–12,000/μm^2^ in the hippocampus^431–433^. GS, expressed specifically in astrocytes, converts glutamate (which is synthesized in astrocytes from glucose) to biologically inactive glutamine, which is exported to extracellular space by Na^+^-dependent neutral amino acid transporters SNAT3 and SNAT5 (SLC38A3 and SLC38A5) and imported into neurons by SNAT1 and SNAT2 (SLC38A1 and SLC38A2)^434,435^. All components of the glutamate(GABA)–glutamine cycle are regulated by transmembrane Na^+^ gradients, which drive the transporters, and by cytosolic Na^+^ concentration, which influences GS activity^436,437^.

Deficient glutamate clearance, usually associated with downregulation of EAAT transporters is one of the primary mechanisms of glutamate excitotoxicity leading to neuronal death (see Table 2 and the previous section). Failure of glutamate uptake is particularly prominent in ALS, in Wernicke encephalopathy and in neurotoxic poisoning with trace metals^138,148,248,438^. Downregulation of EAAT2 also contributes to the pathogenesis of neurodegenerative diseases, epilepsy, migraine and depression, as described in the previous sections. Incidentally, cognitively preserved patients with AD pathology exhibited higher astrocytic expression of EAAT2^439^.

Targeting glutamate clearance by boosting the expression and function of EAAT2 is considered to be a promising therapeutic strategy. Expression of EAAT2 protein can be induced by β-lactam antibiotic ceftriaxone, which attenuated symptoms and increased survival of SOD^G93A^ ALS model mice^440^. Ceftriaxone also showed beneficial effects in β-amyloidosis mouse models^441,442^. A pyridazine derivative LDN-212320, which appears to potentiate EAAT2 translation, demonstrated beneficial effects in SOD^G93A^ mice^443^ and improved cognitive performance in the APP_Sw,Ind_ AD mouse model^444^. Trolox, a derivative of vitamin E, was shown to normalize β-amyloid-induced mislocalization of EAAT2 in astrocytes under the action of^445^. Treatment with minocycline, dexamethasone and histamine may also increase the expression of EAAT2^446^. Finally, the benzothiazole Riluzole, which enhances EAAT2 expression and glutamate clearance, has been approved and shown modest beneficial effects on ALS progression^447,448^.

### Complement system—alleviating post-ischemic damage

Whereas the importance of the complement system in defense against pathogens has been recognized for over a century, its homeostatic and non-immune functions—particularly in the CNS—have only begun to be elucidated recently. Astrocytes are the major source of complement system proteins, including the third complement component (C3), and through complement receptors expressed on their cell membrane, astrocytes receive and respond to complement-derived signals sent by other cells^449^.

Astrocytes express a G-protein-coupled receptor for a complement peptide C3a, C3aR^450–452^ and TLQP-21, a neuropeptide derived from the neurotrophin-inducible protein VGF^453,454^. The expression of C3aR by astrocytes is increased by ischemia^450,451^. Activation of C3aR inhibits the adenylyl cyclase pathway, and stimulates the phospholipase C pathway, leading to Ca^2+^ signaling originating from the endoplasmic reticulum Ca^2+^ store^455^. C3a–C3aR signaling modulates the activity of the extracellular signal regulated kinase 1/2 (ERK1/2) cascade including Ras and c-Raf^456,457^. By inhibiting ERK signaling-mediated apoptotic pathway and caspase-3 cleavage, C3a promotes survival of astrocytes exposed to ischemic stress^458^. In vitro, C3a induces astrocyte expression of cytokines such as IL-6, IL-8 and nerve growth factor (NGF)^459–461^. Astrocytes are involved in or directly mediate the neuroprotective effects of C3a in vitro^462^. A study assessing the effects of C3a on expression of *Gfap*, *Nes*, *C3ar1*, *C3*, *Ngf*, *Tnf* and *Il1b* in naive astrocytes, astrocytes challenged with ischemia and astrocytes exposed to lipopolysaccharide showed that C3a downregulated the expression of *Gfap*, *C3* and *Nes* in astrocytes after ischemia, and increased the expression of *Tnf* and *Il1b* in naive astrocytes and the expression of *Nes* in astrocytes exposed to lipopolysaccharide, but did not affect the expression of *C3ar1* or *Ngf*^463^. Evidently, the responses of astrocytes to C3a are context dependent.

**Fig. 9:**
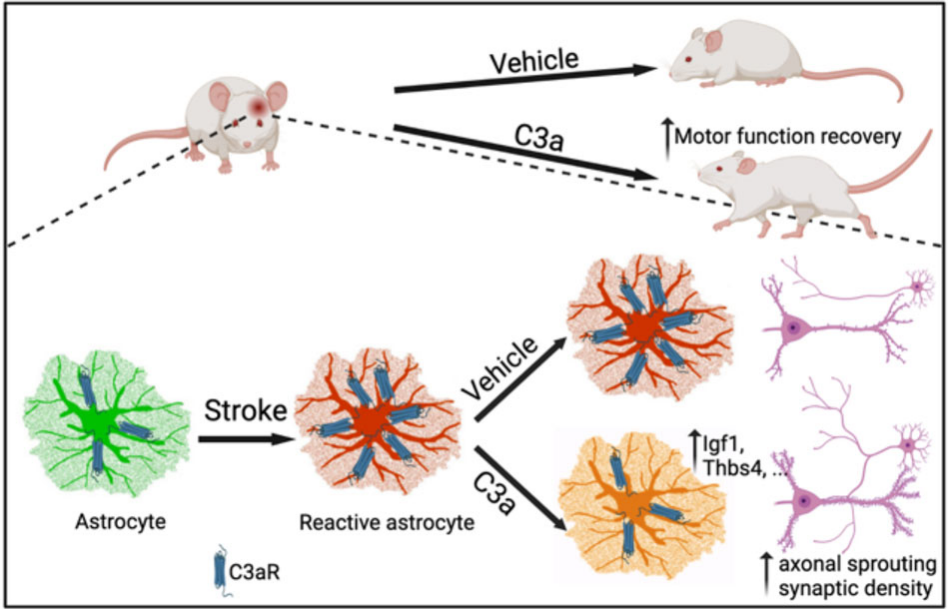
C3aR modulates astrocyte reactivity after stroke, and mice treated with C3a show better recovery of motor function. C3a treatment starting 1 week after stroke reduces astrocyte reactivity and enhances the expression of positive regulators of neural plasticity, increasing synaptic density and axonal sprouting. Figure created in BioRender.

Combining genetic loss-of-function (constitutive C3aR ablation) and gain-of-function (transgenic overexpression of C3a in the brain) approaches showed that C3aR downregulates peri-infarct astrocyte reactivity, as assessed by reduced immunoreactivity of GFAP^383^. Pharmacological modulation of C3aR signaling by daily intranasal treatment of wild-type mice with the C3a peptide for 2 weeks starting 1 week after stroke reduced peri-infarct expression of GFAP (Fig. 9), with this effect being similar to that of transgenic C3a overexpression. Peri-infarct GFAP expression was also reduced in mice that received daily intranasal C3a treatment for 3 weeks and were followed up for 4 weeks after treatment cessation^383^. Expression of GFAP in peri-infarct cortex at this time point was inversely correlated with improved motor function^383^. Cellular deconvolution of bulk transcriptomic data from the peri-infarct cortex showed that cells with molecular signature similar to the so-called disease-associated astrocytes, originally described in the vicinity of β-amyloid plaques in a mouse model of AD and in the aged brain^464^, are present also in post-stroke tissue^383^. This astrocyte subpopulation, expressing high levels of *Gfap*, *Vim* and *C3ar1*, was reduced in mice treated with intranasal C3a. Notably, the expression profile of peri-infarct astrocytes was enriched in genes involved in the regulation of inflammatory response and responses to virus and wounding^383^. By contrast, the subpopulation of astrocytes expressing low levels of *Gfap* and enriched in genes involved in neural plasticity was reduced in the peri-infarct cortex, but only in mice treated with physiological saline^383^.

Reduced peri-infarct astrocyte reactivity in C3a-treated mice was associated with upregulation of *Igf1* and *Thbs4*, coding for insulin-like growth factor 1 (IGF-1) and thrombospondin-4 (THBS4), respectively, both of which are highly expressed in peri-infarct astrocytes^383^. IGF-1 stimulates axon outgrowth ^465^, and THBS4 promotes synaptogenesis of excitatory synapses^466^. The C3a-treated mice had also higher density of excitatory synapses and higher expression of the membrane phosphoprotein growth-associated protein 43^467^, which is an established marker of axonal sprouting and plasticity^468,469^. Treatment with C3a starting 7 days after stroke accelerated recovery of motor function^383,467^, stimulated global white matter reorganization and increased peri-infarct structural connectivity^383^. The increase in expression of positive regulators of neural plasticity provides a plausible mechanistic link between the modulation of astrocyte reactivity, enhanced neural plasticity in the peri-infarct motor cortex, and improved motor function recovery in C3a-treated mice.

In conclusion, GFAP-expressing reactive astrocytes appear as negative regulators of neuronal functioning in the chronic phase after stroke, and C3aR signaling modulates astrocyte reactivity in the post-stroke brain. C3a administered intranasally starting 1 week after stroke reduces astrocyte reactivity, with a positive effect on recovery of motor function. As pharmacological treatments to enhance rehabilitation and improve outcomes in the post-acute and chronic phase after stroke are lacking, clinical testing of C3aR agonists is warranted. Given the broad therapeutic window, the majority of stroke survivors, including those who received but were not helped by the acute interventions, would potentially benefit from such a treatment.

### Connexins—suppressing hemichannels

Connexins (Cx) are a family of membrane channel proteins that, in vertebrates, assemble into connexons. These connexons can form intercellular gap junction channels or plasmalemmal pores, known as hemichannels^470^. Specific to astrocytes are connexins Cx43 (the most abundant) and Cx30^470^. In the CNS, gap junctions connect astrocytes and oligodendrocytes into ‘panglial’ syncytia, enabling long-distance functional integration of glial cells, supporting intercellular signaling via ions, metabolites and second messengers, and maintaining glial isopotentiality^471–473^. Nonjunctional or unpaired connexons, generally known as hemichannels, act as plasmalemmal channels, distinguished by permeability to relatively big (~1 kDa) molecules^474^. Connexins also exert numerous nonchannel functions: they regulate astrocyte morphology and interactions with synapses, influence gene expression, control glial reactivity and modulate mitochondrial function and ATP production^475–477^. In pathological settings, connexins are redistributed from gap junctions to hemichannels; the latter contribute to pathogenesis, and their pharmacological suppression provides beneficial outcomes.

#### Acute brain damage—trauma and ischemia

Acute trauma, ischemia and stroke are characterized by prominent increases in the presence and activity of Cx43 hemichannels in astrocytes, contributing to the secondary damage of the nervous tissue^478^. Increased hemichannel activity is associated with the release of glutamate and ATP, which can trigger excitotoxicity^479^. Furthermore, in the context of brain trauma, Cx43 appears to activate cytolytic P2X_7_ purinoceptors and downregulate astrocytic expression of EAAT1, both events promoting excitotoxicity^480^. In hypoxia and ischemia, opening of Cx43 hemichannels can lead to astrocytic depolarization and death^481^. In cerebral ischemia, Cx43 hemichannels do not contribute to an acute phase injury but rather propagate secondary damage in the aftermath^481^.

#### Neurodegeneration and AD

Increased presence and activity of astrocytic Cx43 hemichannels seems to be a common feature of all major neurodegenerative pathologies, including AD, PD, HD, ALS and MS^482–487^. Exposure of astrocytes to various β-amyloid peptides, and β-amyloid_25–35_ in particular, increased Cx43 expression and appears to redistribute connexins from gap junction assemblies to hemichannels^486,488,489^, the latter, in turn, mediating excessive release of glutamate and ATP resulting in excitotoxicity^489–491^. Expression of Cx43 is significantly upregulated in AD post-mortem brain tissue and in AD murine models of β-amyloidosis such as APP_swe/_PS1_dE9_ mice and 5xFAD mice^489,492,493^. Post-mortem studies also revealed increased Cx43 protein levels in astrocytes surrounding β-amyloid plaques^494^, although, paradoxically, levels of *Gja1* (encoding Cx43) mRNA were decreased^492^, suggesting post-transcriptional dysregulation. Besides potentiating the expression of Cx43, exposure to β-amyloid opens Cx43 hemichannels, as shown by ethidium bromide uptake assay^489,490^. Activity of Cx43 hemichannels is high in reactive astrocytes surrounding β-amyloid plaques^495^. The opening of Cx43 hemichannels can be induced by cytosolic Ca^2+^ signals or by a reduction in cholesterol^491^. Notably, the gap junctional connectivity of astrocytes in the hippocampus of APP_swe/_PS1_dE9_ mice remains unaffected^489^. Finally, a profound decrease in astrocytic expression of Cx43 was found in the post-mortem samples of patients with advanced-stage PD; the degree of the Cx43 loss correlated with the severity of symptoms, including nonmotor symptoms such as depression and sleep irregularities^209^.

#### Neuropathic pain

In the spinal cord, astrocytic Cx43 expression is upregulated following nerve injury, chemotherapy or spinal cord injury^496–499^. An increase in the activity of astrocyte Cx43 hemichannels contributes to the central sensitization of pain. Genetic ablation of astrocytic Cx43 reduces neuronal hyperactivity and mechanical allodynia in rodent models of neuropathic pain^500^. Similarly, the knockdown of Cx43 by small interfering RNA intrathecal administration reduced mechanical allodynia in a spinal nerve ligation model^501^. Mechanistically, opening of Cx43 hemichannels leads to the release of ATP and glutamate, which promotes spinal neuronal hyperactivity, reactive astrogliosis and microgliosis^498,502,503^. Suppression of Cx43 hemichannels via intrathecal injection of blockers such as Gap26, Gap27 or Peptide 5 reduces the production of chemokines CXCL1 and CXCL12, as well as cytokines IL-1β and IL-6, in various neuropathic pain models, including nerve injury, chemotherapy-induced and bone cancer models^496,498,504,505^.

#### Therapeutic targeting of Cx43 hemichannels

**Fig. 10:**
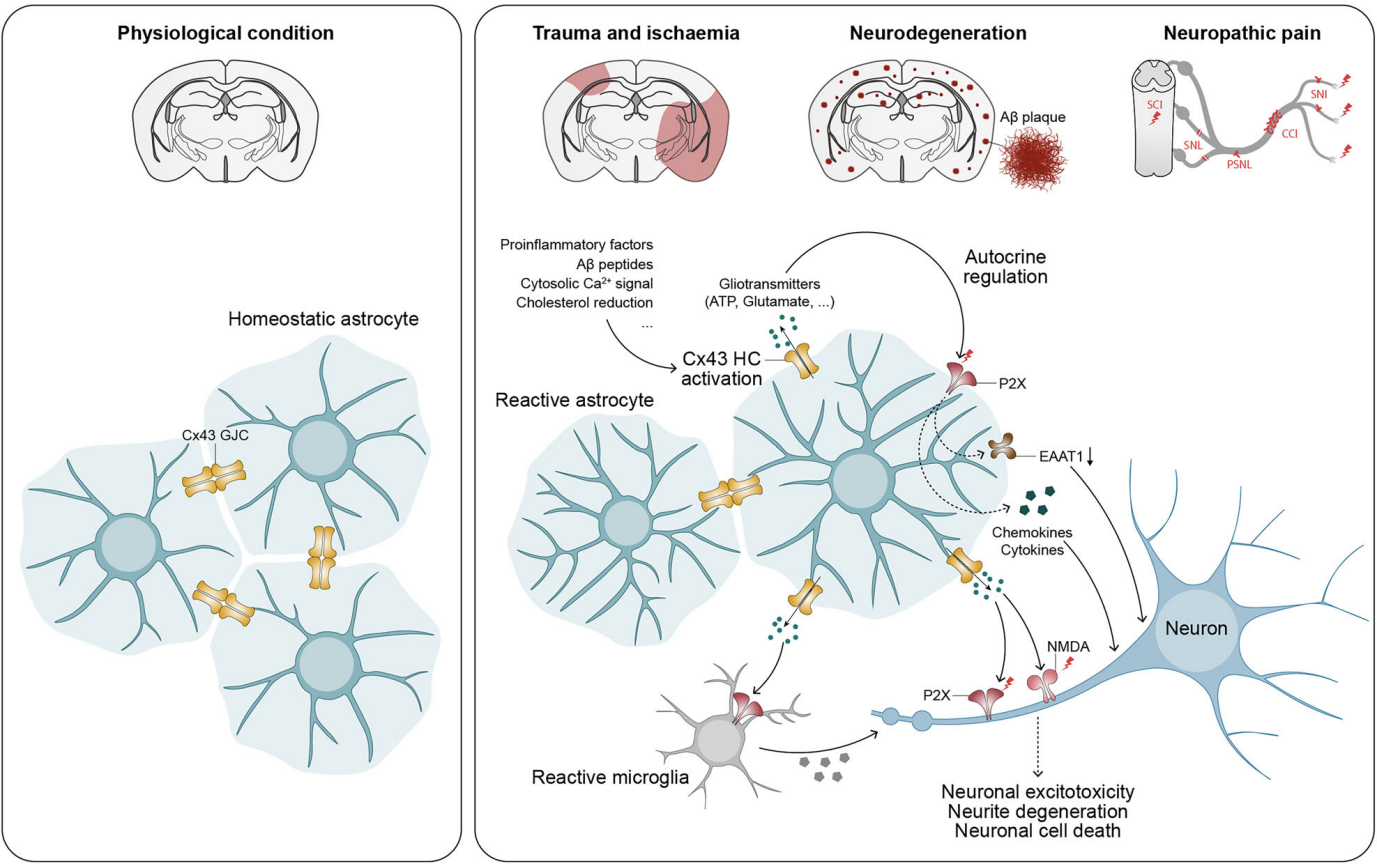
Astrocytic connexin (Cx) in health and diseases. In physiological conditions, Cx43 forms gap junction channels (GJCs) that mediate the formation of astrocytic network. However, in pathological conditions, such as trauma, ischemia, neurodegeneration and neuropathic pain, pathogenic insults and neuroinflammation trigger reactive astrogliosis, accompanied by the activation of Cx43 hemichannels (HCs), with its GJC function unaltered or downregulated. Cx43 hemichannels contribute to the release of ATP and glutamate. ATP can activate P2X receptors on astrocytes in an autocrine manner, suppress EAAT expression and increase the release of chemokines and cytokines. ATP can also activate P2X receptors on microglia, promoting reactive microgliosis. In addition, ATP and glutamate activate P2X and NMDA receptors on neurons, respectively. These processes downstream of Cx43 HC activation collectively contribute to neuronal excitotoxicity, degeneration and even cell death.

Targeting astrocyte connexin hemichannels has disease-modifying effects in various neurological diseases (Fig. 10). Several Cx43 targeting drugs underwent clinical trials^506^. Antisense oligodeoxynucleotides (AsODNs) targeting Cx43 expression have proven effective in the treatment of skin wounds, corneal injuries, and retinal disorders^506^. Recently, a fiber-hydrogel scaffold-mediated Cx43-AsODN delivery system was tested for the treatment of spinal cord injury and was shown to suppress reactive gliosis and reduce neuronal loss^507^. A peptide mimetic, aCT1, which reduces Cx43–ZO1 interaction to preserve gap junction connectivity while inhibiting Cx43 hemichannels, has been tested in clinical trials for skin wounds, corneal injuries and retinal disorders^506,508^. However, the effect of these drugs in neurological diseases requires further testing in vivo.

Several other peptides suppress Cx43 hemichannels and alleviate neurological symptoms. Intrathecally injected Cx43 blockers Peptide5, Gap26 and Gap27, for example, reduced neuropathic pain^496,498,509^. Similarly, Gap26 and Gap27 prevented increases in extracellular glutamate and caspase-3 and significantly reduced cerebral infarct volume^480^. Infusion of Peptide 5, a selective Cx43 hemichannel blocker, 90 min after a 30-min period of bilateral carotid artery occlusion, thus limited brain damage^510^.

All these peptide hemichannel inhibitors, however, cannot cross the blood–brain barrier, which limits their clinical potential. In addition, these peptides are mimetic peptides targeting the extracellular loop of Cx43, which have prominent off-target effects, interfering with the channel functions of Cx32, Cx37 and Cx40^511^. Gap26 and Gap27 could also inhibit gap junction channel function of Cx43^512,513^, possibly by blockage of the docking sites of opposing hemichannels. Gap19, a mimetic peptide of the Cx43 intracellular loop, inhibits hemichannel opening by suppressing the tail–loop interaction without impacting on the gap junction function^514^. As the target of Gap19 is intracellular, an addition of cell-permeable sequence TAT to Gap19 produced a TAT–Gap19 peptide, which improves the hemichannel inhibitory efficacy (IC_50_ ~7 μM versus 50 μM for Gap19) and makes this formulation to cross blood-brain barrier^515^. Another peptide, TAT–Cx43_266–283_, mimics the Cx43 C terminus and can suppress Cx43 hemichannels without affecting gap junction communication^516,517^. Both TAT–Gap19 and TAT–Cx43_266–283_ effectively alleviate symptoms in mouse models of epilepsy^516,518^. However, peptide drugs suffer from a major drawback of short half-life in the circulation, which limits their bioavailability and their therapeutic potential in chronic neurodegenerative diseases^519^. This problem was recently solved by using lipid nanoparticle to package TAT–Cx43_266-283_ (TAT–Cx43@LNP), which achieve long-term retention in the brain and effectively alleviate cognitive decline in a mouse model of AD^517^.

Alternative strategies utilize small molecules to target Cx43 hemichannels. Boldine, a natural alkaloid that blocks hemichannels, reduced the release of ATP and glutamate, and alleviates neuronal damage in APP/PS1 mice^520^. A carbenoxolone derivative INI-0602 with high blood–brain barrier penetration blocks hemichannels and alleviates memory deficits in APP/PS1 mice after intraperitoneal administration^521^. A recent study used computational screening to develop a small-molecule compound D4, which shows high efficiency in blocking Cx43 hemichannels (10 nM) without affecting gap junction function (up to 200 μM)^522^. D4 could be orally administrated, readily crosses the blood–brain barrier and effectively suppresses reactive gliosis in an epilepsy model^523^. However, D4 also shows off-target effects blocking other connexin channels, including Cx26, Cx30 and Cx45^522,523^.

### MAO-B—reversing tonic inhibition and oxidative stress

Considering that reactive astrocytes contribute to various neurological disorders, including neurotrauma, AD, PD, stroke, metabolic disorders and epilepsy, targeting MAO-B and astrocytic GABA tonic inhibition may provide a broad-spectrum therapeutic strategy.

#### Alzheimer’s disease

In AD, reactive astrocytes cluster around β-amyloid plaques and exhibit increased MAO-B expression, resulting in heightened GABA synthesis and tonic inhibition of neuronal circuits^524^. Excessive astrocytic GABA release suppresses excitatory transmission, impairs synaptic plasticity and contributes to memory dysfunction^524^. In contrast to the healthy brain, where tonic inhibition fine-tunes neuronal excitability, in disease, excessive astrocytic GABA secretion suppresses excitatory transmission, reduces glutamatergic input and disrupts memory-related processes^524–527^. Under physiological conditions, hippocampal astrocytes contain low [GABA]_i_. However, in APP/PS1 mice, which develop AD-related β-amyloid plaques, periplaque reactive astrocytes exhibit significantly increased GABA immunoreactivity; notably, β-amyloid directly upregulates MAO-B expression, linking β-amyloid pathology with aberrant astrocytic regulation of neurotransmission^510^. In the APP^NL-F^ mouse model of β-amyloidosis, increased tonic GABA tone was observed even before the development of plaques^276^. Elevated astrocytic GABA is associated with increased GFAP immunostaining characteristic of reactive astrocytes^524,528^. In reactive astrocytes, Best-1, the GABA-releasing channel, redistributes from the perisynaptic leaflets to the soma, probably reflecting a pathological rearrangement of gliotransmission that favors GABA over glutamate^529^. A positive correlation between GABA levels and proximity to senile plaques suggests localized induction of MAO-B activity, reinforcing its pathological significance^524^). Imaging studies using PET tracers such as [^18^F]SMBT-1 confirm that MAO-B is associated with reactive astrogliosis in AD and shows strong correlations with β-amyloid and tau pathology^367,530^. Besides GABA-related effects, H_2_O_2_ generated in the process of MAO-B-catalyzed putrescine conversion evokes oxidative stress and consequent neuronal damage^531^. Increased H_2_O_2_ amplifies reactive astro- and microgliosis, potentiates lipid peroxidation and perpetrates mitochondrial malfunction, further exacerbating AD progression^346^. Pharmacological occlusion of MAO-B with selective inhibitors reduces astrocyte reactivity, restores synaptic function and mitigates cognitive deficits in amyloidosis model mice^532,533^.

Excessive astrocytic GABA synthesis and release lead to abnormal synaptic transmission and cognitive deficits. Tonic GABA release in the dentate gyrus disrupts synaptic plasticity by persistently activating extrasynaptic GABA_A_ and GABA_B_ receptors, thereby diminishing excitatory neurotransmission and long-term potentiation, favoring synaptic depression and memory impairment^534^. Blocking MAO-B with deprenyl restores synaptic transmission and plasticity in APP^NL-F^ mice^276^. Chronic synaptic inhibition reduces neuronal excitability, leading to synaptic depression and memory impairment^535^. Post-mortem human studies confirmed this mechanism, demonstrating elevated GFAP, MAO-B and GABA expression in reactive astrocytes within the temporal cortex, with a strong correlation between GFAP and MAO-B levels^345^.

#### Parkinson’s disease

MAO-B contributes to the pathogenesis of PD by boosting astrocytic GABA transmission and contributing to oxidative stress. Expression of MAO-B is upregulated in reactive astrocytes in the substantia nigra, leading to excessive GABA production and oxidative stress. This astrocyte-derived GABA mediates tonic inhibition, thus suppressing the excitability of neighboring dopaminergic neurons. As a result, the synthesis and release of dopamine is blocked, markedly reducing dopamine levels and worsening motor impairments^348,536–538^. Post-mortem analyses of PD brains consistently demonstrated elevated MAO-B activity in reactive astrocytes^346^. The loss of dopaminergic neurons is also exacerbated by oxidative stress triggered by H_2_O_2_ produced by MAO-B activity^539^. Neuronal death, in turn, amplifies secondary reactive gliosis, creating a feedforward loop exacerbating neurodegeneration in PD.

#### Neuropathic pain

Astrocytic MAO-B contributes to neuropathic pain by providing for an excessive GABA release in the spinal dorsal horn, thus altering chloride homeostasis and promoting neuronal hyperexcitability^265,540^. The pathogenesis of the chronic pain involves BDNF (microglia-derived)-dependent downregulation of neuronal KCC2 transporter leading to a depolarizing shift of Cl^−^ reversal potential (*E*_Cl_). Thus, opening of GABA_A_ receptors generates Cl^−^ efflux and neuronal hyperexcitability^541^. Increased astrocytic GABA synthesis, driven by upregulated MAO-B activity, further amplifies this pathological excitation by acting on extrasynaptic GABA_A_ receptors containing the α5 subunit. Furthermore, MAO-B-dependent astrocytic GABA release contributes to metabolic dysregulation in neuropathic pain. [^18^F]FDG-microPET imaging of chronic pain animal models revealed increased glucose metabolism in the ipsilateral dorsal horn, correlating with heightened neuronal excitability and glial reactivity^265^. Beyond its role in neurotransmission, MAO-B contributes to reactive astrogliosis and neuroinflammation. Thus, MAO-B-mediated astrocytic GABA synthesis and its involvement in neuronal tonic excitation augments synaptic transmission and regional metabolism, contributing to the development of neuropathic pain.

#### Therapeutic targeting of astrocytic MAO-B

Given its prominent role in neurodegeneration and inflammation, astrocytic MAO-B represents a promising therapeutic target. Pharmacological inhibitors such as selegiline, rasagiline and newer reversible inhibitors such as KDS2010 are effective in mitigating MAO-B-mediated pathology^531^. These inhibitors reduce astrocytic reactivity, normalize GABAergic neurotransmission and limit oxidative stress, offering potential treatment strategies for several neurological disorders including AD, PD, neuropathic pain, stroke and others. Furthermore, gene-silencing approaches targeting astrocytic MAO-B have demonstrated neuroprotective effects in preclinical models, highlighting potential for future genetic therapies^538^.

Pharmacological inhibitors of MAO-B are classified into irreversible (selegiline and rasagiline) and reversible inhibitors (safinamide and KDS2010)^531,542^. While irreversible inhibitors were widely used in clinical settings due to their high potency (IC_50_ ~10 nM), their limited selectivity (selegiline, ~150-fold; rasagiline, ~52-fold over MAO-A) results in potential off-target effects, complicating their long-term efficacy^348^. Moreover, prolonged treatment with irreversible inhibitors can trigger compensatory mechanisms, including the upregulation of DAO-dependent GABA synthesis, thus undermining the therapeutic impact^530,531^. By contrast, highly selective reversible inhibitors, such as KDS2010 (~12,500-fold selectivity for MAO-B over MAO-A), demonstrated superior efficacy in mitigating aberrant astrocytic GABA release and oxidative stress without inducing compensatory pathways^543^. Preclinical studies demonstrated that KDS2010 restores dopaminergic function, alleviates motor deficits and reduces neuroinflammation in PD models, positioning it as a promising candidate for future disease-modifying interventions^531^. The ongoing development of highly selective MAO-B inhibitors and gene-silencing approaches targeting astrocytic MAO-B further underscores the potential of this enzyme as a therapeutic target in PD. Pharmacological inhibition of MAO-B using compounds such as selegiline or safinamide reduces oxidative stress, restores synaptic function and improves cognitive performance in AD models^544^.

Similarly, suppression of MAO-B activity is useful in the treatment of neuropathic pain. Pharmacological inhibition of MAO-B using KDS2010 restores GABAergic neurotransmission and alleviates mechanical allodynia and chronic pain symptoms^540^. Treatment with KDS2010 also reduces regional hypermetabolism, suggesting that astrocytic GABA synthesis plays a key role in sustaining pathological neuronal activity. The suppression of neuroinflammation and reduction of astrocyte-mediated excitatory signaling through MAO-B inhibition highlight its therapeutic potential for chronic pain management, reinforcing the importance of targeting astrocytic MAO-B as a novel strategy in neuropathic pain treatment^540^.

### Urea cycle—restoring neuroprotection and limiting oxidative damage

**Fig. 11:**
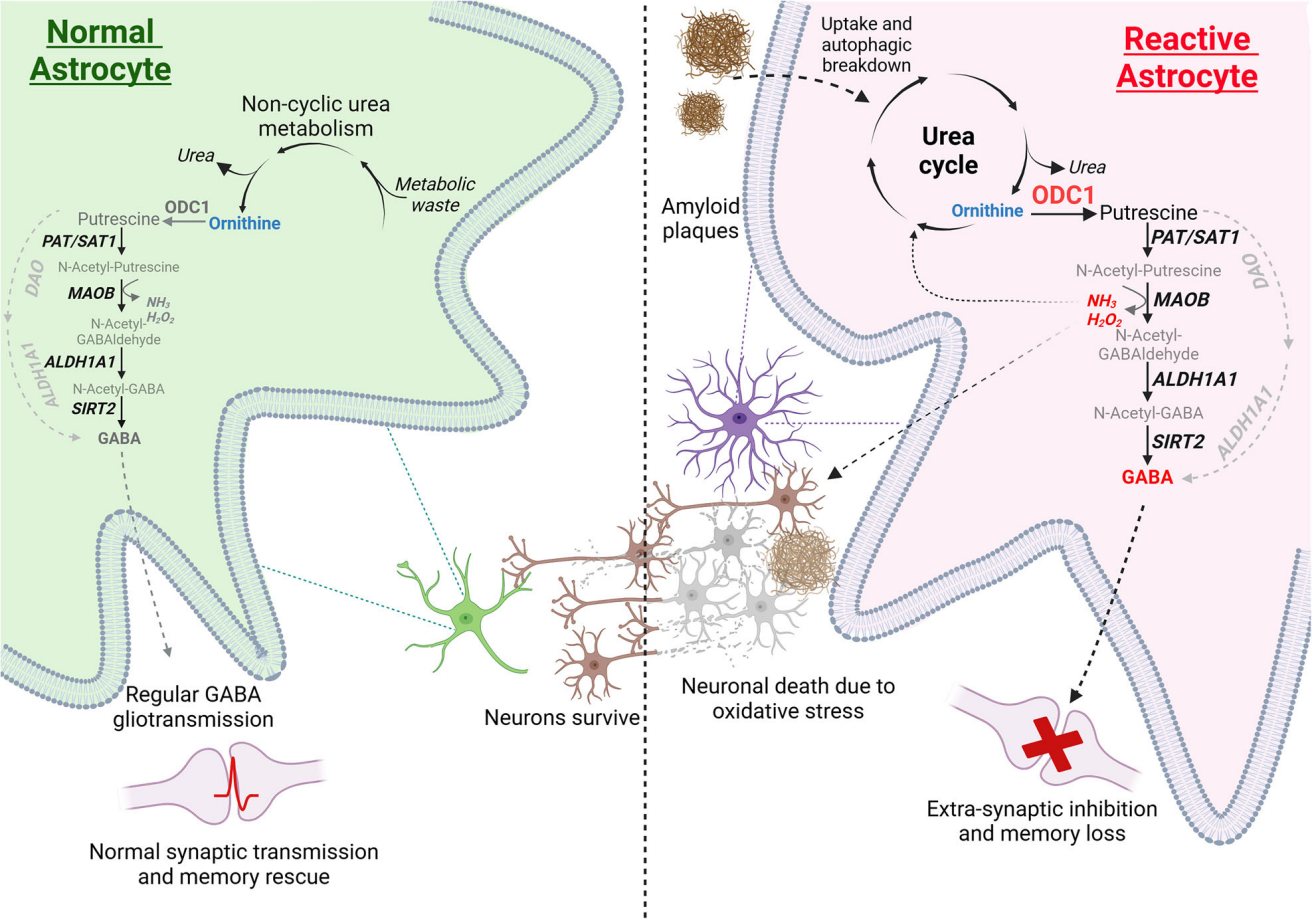
Urea cycle and putrescine degradation in reactive astrocytes in the context of AD. Schematic of the astrocyte putrescine degradation pathway involved in the late steps of toxic protein degradation process in normal (left) and AD (right) conditions. Healthy cortical and hippocampal astrocytes show low levels of activity in putrescine degradation metabolism, while reactive astrocytes show high levels of putrescine and, thus, high activity of putrescine degradation enzymes, including MAO-B, which produces H_2_O_2_, NH_3_ and, ultimately, GABA.

Astrocytes use the urea cycle to clean up excessive β-amyloid; in the context of AD, the urea metabolism undergoes a metabolic switch^545^. The urea cycle, discovered by Hans Krebs and Kurt Henseleit^546^, converts toxic ammonium (obligatory product of amino acids catabolism) to urea. This cycle is highly expressed in hepatocytes, whereas all components of the cycle are also selectively expressed in astrocytes^545^. In the healthy brain, astrocytes process urea in a noncyclic manner; in AD, this enzymatic cascade is upregulated and switched to the urea cycle mode, resulting in both helpful and harmful outcomes^545^ (Fig. 11). The upregulated urea cycle removes β-amyloid and ammonia on one hand, while on the other, overproduction of the urea cycle metabolite ornithine leads to the overexpression of the enzyme ornithine decarboxylase 1 (ODC1), which produces excessive putrescine that subsequently degrades into GABA, H_2_O_2_ and ammonium (see previous section). The produced ammonium feeds back into the urea cycle, thus creating a vicious loop that exacerbates neurotoxicity. An increase in the expression of urea cycle enzymes and ODC1 was detected in astrocytes from patients with AD and mouse models of AD^545^.

Inhibition of ODC1 with difluoromethyl ornithine (DFMO) reduced the production of toxic byproducts and improved memory performance in APP/PS1 mice, a widely used transgenic model of AD. Suppression of ODC1 activity appears to redirect ornithine metabolism toward urea production rather than putrescine, and because urea can be efficiently eliminated from astrocytes via the bloodstream and excreted in urine, no adverse effects were observed under the reported treatment regimen. Notably, DFMO-treated APP/PS1 mice also exhibited a reduction in hippocampal β-amyloid plaque burden, suggesting enhanced clearance mechanisms^547^. With prolonged treatment, sustained inhibition of astrocytic ODC1 was associated with near-complete removal of β-amyloid deposits from the brain in this model. While these findings are encouraging, further studies are needed to evaluate the long-term safety, reproducibility and translational applicability across additional models and treatment paradigms. Besides reducing β-amyloid load, treatment with DFMO coerced astrocytes into a neuroprotective state. Incidentally, these ‘neuroprotective’ astrocytes had genetic similarities with the astrocytes after environmental enrichment and physical exercise, supporting regeneration of the nervous tissue^548^. The involvement of astrocytic autophagic plasticity upstream to the urea cycle was recently confirmed^549^. In summary, pharmacological inhibition of ODC1 prevents harmful effects downstream to the urea cycle while preserving its beneficial role in clearing toxic substances. At the same time, while DFMO shows promise in animal models, it has failed in a single-subject case study^550^ and causes side effects during long-term administration, prompting the need for better therapeutics.

### Astrocytic α7 nicotinic acetylcholine receptors—containing β-amyloid deposition

**Fig. 12:**
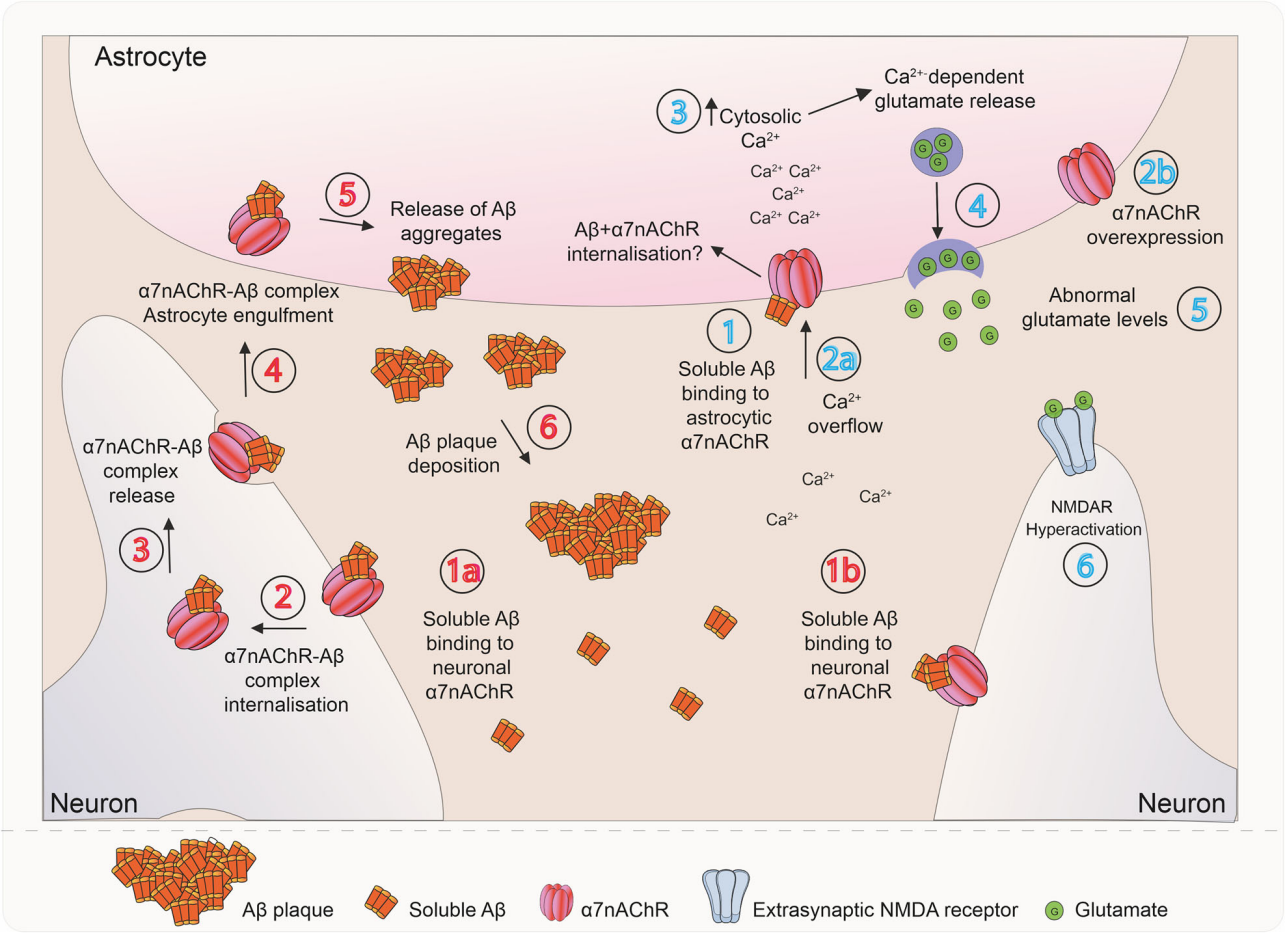
Hypothetical model of the pathophysiological role of astrocytic α7nACh receptors in AD. Soluble β-amyloid forms a complex with α7nACh receptors (step 1a,b) on the neuronal membrane followed by internalization of the complex (step 2). The neuron releases the α7nACh receptor/β-amyloid complex (step 3), which is taken up by reactive astrocyte (step 4) and undergoes lysis, and β-amyloid aggregates are secreted to the extracellular space and form senile plaques (steps 5 and 6). In addition, soluble β-amyloid interacts and forms a complex with α7nACh receptors on the astrocytic membranes (step 1), inducing α7nACh receptors overexpression (step 2b). High β-amyloid causes increased Ca^2+^ influx through α7nACh receptors into astrocytes (steps 2a and 3), which triggers a Ca^2+^-dependent glutamate release (steps 4 and 5), followed by hyperactivation of extrasynaptic NMDA receptors leading to glutamate excitotoxicity and neuronal death (step 6). Based on ref. ^559^.

The α7 nicotinic acetylcholine (α7nACh) receptors highly expressed in the human brain mediate cholinergic neurotransmission and neurobiological processes fundamental for memory consolidation and inflammatory responses^551^. Homomeric α7nAChR is assembled from five α7 subunits forming a cation channel with high permeability to Ca^2+^ with P_Ca_/P_monovalent_ ≈ 6 (ref. ^552^). Opening of α7nACh receptors thus generates Ca^2+^ influx modulating various signaling cascades and inflammatory responses essential for cell survival and memory consolidation. The α7nACh receptors are also expressed in astrocytes and are linked to generation of Ca^2+^ signals^553^. In the early 2000s, it was noted that α7nACh receptors bind β-amyloid with high (picomolar range) affinity^554,555^. In cortices and hippocampi of sporadic AD, expression of α7nACh decreases in neurons, while increasing in astrocytes^556,557^. Even higher increases in astrocytic α7nACh receptors were found in autosomal-dominant AD, with significant positive correlation between numbers of senile plaques and α7 nACh receptor expression^558^. A recent hypothesis postulated that overexpression of α7 nAChRs in astrocytes is an early modulator of β-amyloid pathology in the context of AD^1,559^ (Fig. 12). Because reactive astrogliosis in AD precedes β-amyloid deposition^355,356^, it was proposed that astrocytes are involved in the spreading of β-amyloid pathology. Soluble β-amyloid is suggested to form a complex with α7nACh receptors on neuronal membrane, leading to the release of this complex to be taken up by the astrocytes. Subsequently, β-amyloid is released to the extracellular space as an aggregate and forms senile plaques (Fig. 12, step 1). Uptake of the soluble α7ACh receptor/β-amyloid complex by astrocytes triggers Ca^2+^ influx, followed by glutamate secretion, which induces excitotoxicity and neuronal death (Fig. 12, step 2).

To further understand the functional role of astrocytic α7nACh receptors in the healthy brain as well as in different disorders, it is essential to develop highly specialized molecular tools capable of detecting α7nACh receptors in the brain of living individuals. [^18^F]ASEM was developed as a first-generation PET radiotracer that binds to α7nACh receptors with nanomolar affinity^560^. The PET scans demonstrated increased binding in patients with MCI across all brain regions^561^. A second-generation tracer [^11^C] Kln83 with improved selectivity was subsequently designed and is now used in clinical settings^562,563^.

### Aquaporin 4—manipulating astrocytic water fluxes to manage edema

**Fig. 13:**
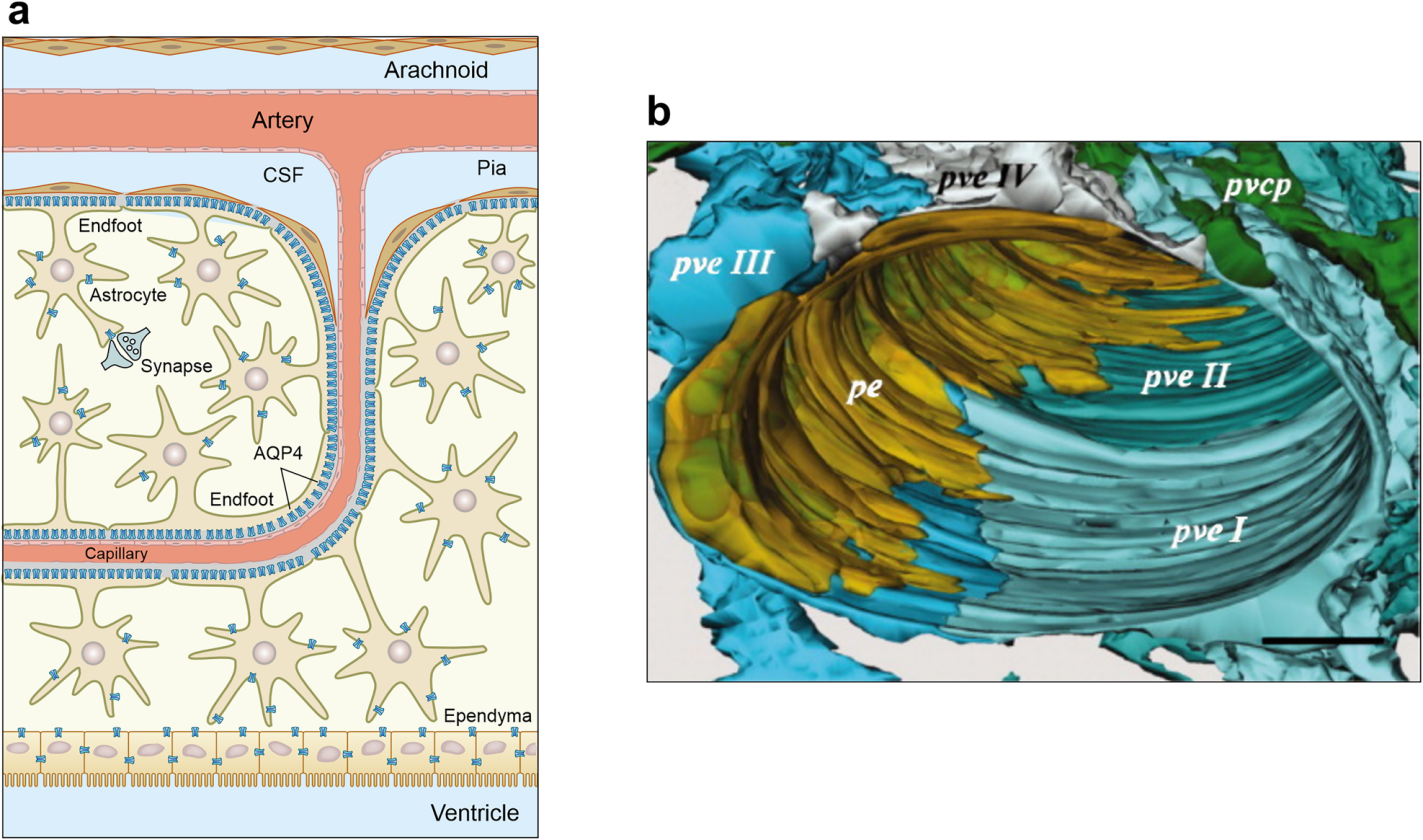
Aquaporin 4 and astrocyte–vascular interface. **a** Schematic illustration of AQP4 expression in brain. AQP4 (blue symbols) is expressed in astrocytes and ependymocytes, and is particularly enriched in astrocytic endfeet abutting on brain microvessels and in astrocytic processes underneath the pial surface. Lower levels of AQP4 are found in perisynaptic astrocytic membranes. Modified from ref. ^571^. **b** A 3D reconstruction based on serial electron micrographs indicating that capillaries are completely covered by a perivascular glial sheath. Four astrocytic endfeet (pve I–IV) are shown together with a pericyte (pe) that is sandwiched between the capillary and the endfeet. A cell process (pvcp), probably of microglial origin, is seen peripheral to the glial sheath. Reproduced, with permission from ref. ^568^.

The water channel Aquaporin-4 (AQP4) is abundantly expressed in astrocytes and ependymoglia; it is particularly enriched in the astrocytic endfoot membranes that abut brain capillaries^564^ (Fig. 13). As such, AQP4 is uniquely positioned to impact the exchange of water between blood and the brain. Attesting to such a role, specific deletion of the perivascular pool of AQP4 significantly reduces the size of experimentally induced edema^565^, mimicking the effect of a global AQP4 knockout^566^.

Non-invasive MRI based on multiple echo time arterial spin labeling has confirmed that water flux between blood and brain tissue is reduced in animals following targeted deletion of AQP4^567^. That perivascular endfoot membranes and their AQP4 pools are rate limiting when it comes to water exchange across the brain–blood interface, is consistent with three-dimensional (3D) electron microscopic reconstructions showing that astrocyte endfeet provide a complete covering of brain microcapillaries^568^. The wealth of data supporting a role for AQP4 in mediating water flux across the brain–blood interface suggests the potential use of AQP4 inhibitors in the treatment of stroke and other conditions associated with brain edema. In theory, such inhibitors would curb edema formation, but also impact infarct size. Thus, deletion of AQP4 has been found to reduce infarct volume not only after middle cerebral artery occlusion (which is associated with a massive edema^565^), but also in experimental models where edema is much less of a confounding factor (distal middle cerebral artery occlusion^569^). The mechanisms underlying the specific effect on infarct volume remain to be identified, but one possibility is that the protection is mediated through increased astroglial activation in the border zone of the infarct^569^.

The development of clinically useful AQP4 inhibitors has proved difficult for several reasons^570^. Any attempt to inhibit AQP4 in a clinical setting must take into account the fact that AQP4 allows bidirectional transport of water^571^. Thus, the theoretical time window for treatment will close when the edema transits to its resolution phase and AQP4 switches from being an influx route for water to support water efflux^572^. In the latter phase, treatment with AQP4 inhibitors would make matters worse and slow down recovery rather than speeding it up. These complications notwithstanding, the potential upside of identifying clinically useful AQP4 inhibitors is promising and should inspire further studies and clinical trials.

As an alternative to the search for new inhibitors, one could look for signaling pathways that regulate AQP4 expression at the brain–blood interface or in the brain at large. Recently, it was shown that canonical BMPs—and BMP2 and 4 in particular—cause an upregulation of AQP4 expression in mature astrocytes and dysregulate the associated dystrophin complex by differentially affecting its individual members^573^. BMP signaling pathways could be considered as a target for putative treatment strategies. Another strategy relies on the redistribution of AQP4, which is at least in part regulated by calmodulin. Inhibition of calmodulin by trifluoperazine (a drug licensed to treat schizophrenia) suppressed AQP4 localization at the blood–spinal cord barrier. In a model of spinal cord injury, treatment with trifluoperazine resulted in attenuation of edema and acceleration of functional recovery^574^.

Any attempt to pharmacologically manipulate AQP4—directly, through inhibitors, or indirectly, through targeting signaling pathways—must be discussed in the context of the roles that AQP4 normally subserves. It is well known that the function of AQP4 goes far beyond that of a specific water channel. AQP4 is involved in cell volume control and K^+^ homeostasis (through interaction with other membrane proteins^575,576^) and appears to be pro-inflammatory in different experimental models of neurological disease^577,578^. It is also well documented that AQP4 serves as an autoantigen in neuromyelitis optica^579^. Most relevant, however, is the role that AQP4 appears to play in propelling or facilitating the glymphatic system of the brain. The glymphatic system mediates influx of CSF into the brain tissue from vascular spaces around arteries and efflux of interstitial fluid through perivenous spaces. It is well documented that this fluid flow contributes to the clearance of waste products from the brain^580^. Early studies showed that deletion of AQP4 reduces clearance of macromolecular substances from the brain after intrastriatal injections^581^, and a more recent study demonstrated reduced clearance of extracellular solutes after specific deletion of the perivascular pool of AQP4^582^. The perivascular AQP4 pool appeared to be primarily relevant for clearance of larger molecules such as 500-kDa dextran. Pharmacological inhibition of AQP4 by AER-271 suppressed glymphatic influx to brain tissue^583^, supporting the conclusions based on the genetic studies referred to above. Amyloid peptides are among the molecules whose clearance from the brain is sensitive to AQP4 deletion and suppression of the glymphatic system^581^. Potential mechanistic coupling between AQP4 and amyloid clearance is supported by early experimental studies showing that amyloid deposits are typically associated with vessels that lack perivascular AQP4^584^.

It follows from the discussion above that AQP4 has multifarious roles in the brain. Pharmacological manipulation with AQP4 function or expression holds promise in several clinical settings but might have side effects that deserve careful consideration. Most notably, any pharmacological intervention targeting AQP4 could affect functions as diverse as cell volume control, K^+^ homeostasis and clearance of waste products including peptides associated with neurodegenerative disease. After 30 years of research on AQP4, we have a solid grasp on the role of this water channel in brain physiology and pathophysiology. Recent breakthroughs have set the stage for new attempts to target AQP4 for new therapies.

### Astrocytic autophagy plasticity—boosting degradation of pathological proteins

**Fig. 14:**
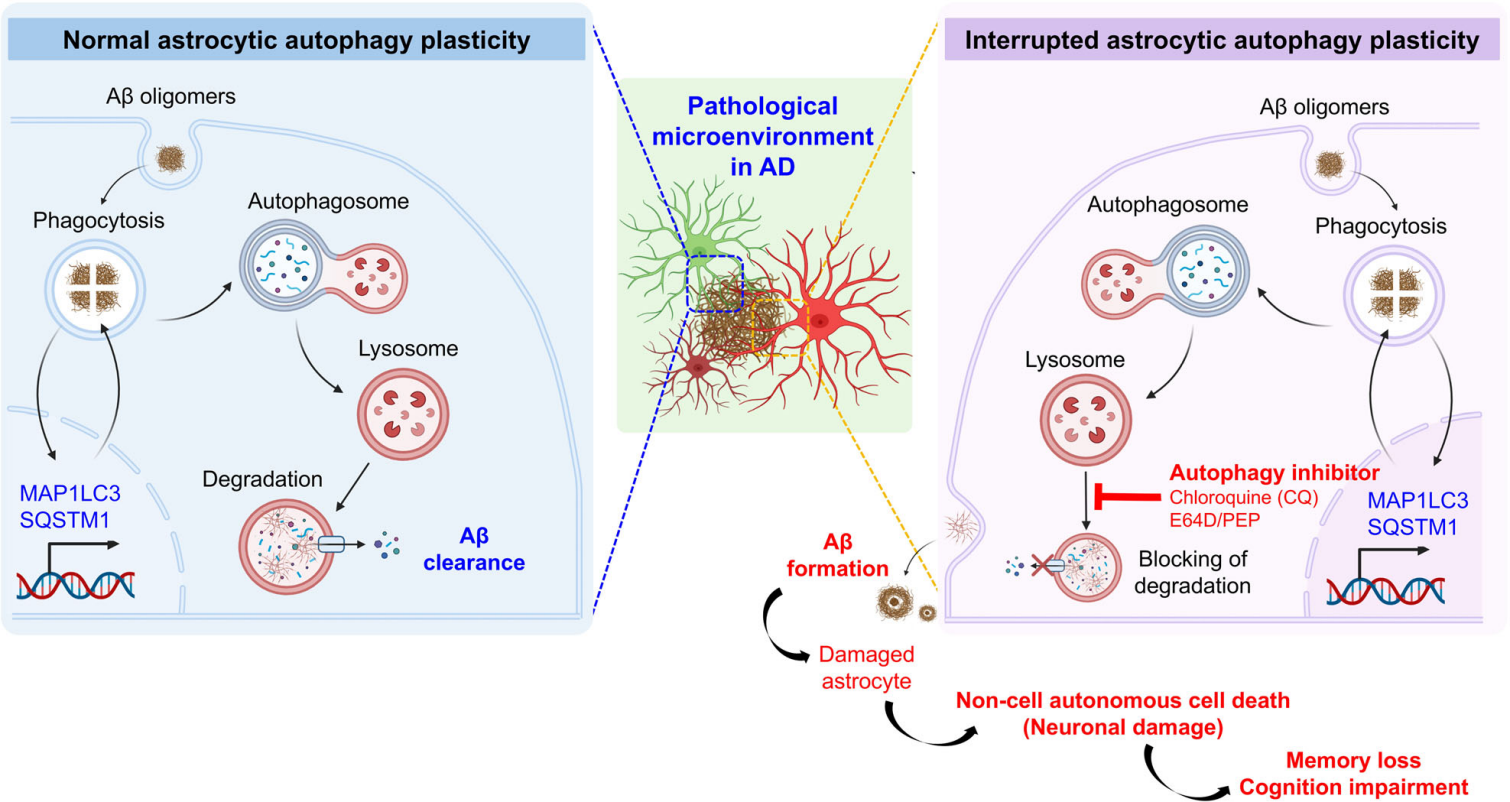
Astrocyte autophagic plasticity in amyloid clearance and degradation. Normal astrocytic autophagy plasticity is activated in response to AD-related stressors such as β-amyloid (Aβ) oligomers through upregulation of MAP1LC3B/LC3B and SQSTM1/p62, promoting phagophore formation, enhancing β-amyloid clearance, supporting astrocyte survival and contributing to improved cognitive function in AD mouse models. By contrast, interrupted astrocytic autophagy plasticity impairs mitochondrial function, increases oxidative stress and induces astrocytic cell death, leading to neuronal damage, exacerbated Aβ plaque accumulation and worsened memory deficits. Drawn based on ref. ^585^.

Neurons exposed to β-amyloid oligomers fail to cope with this proteotoxic stress, leading to a reduction in the expression of autophagy-related genes (MAP1LC3B/LC3B and SQSTM1/p62, two key autophagy-related genes) alongside a decrease in microtubule-associated protein 2 (MAP2). By contrast, astrocytes respond to β-amyloid oligomers by upregulating autophagy-related gene expression. Notably, the expression of MAP1LC3B/LC3B and SQSTM1/p62 is elevated in parallel with the increase in GFAP immunoreactivity. Thus, astrocytes respond to β-amyloid proteotoxic stress by undergoing plastic changes in both morphology and gene expression to clear β-amyloid oligomers through phagocytic autophagy^585^. Astrocytes orchestrate the autophagy response gradually, coordinating the sequential expression of autophagy components. This astrocytic autophagy plasticity facilitates β-amyloid clearance by modulating the expression of autophagy-related genes. Inhibition of autophagic function impairs mitochondrial health, increases oxidative stress and exacerbates astrocytic cell death. These findings suggest that autophagy plasticity plays a role in astrocyte survival under neurodegenerative conditions. Moreover, suppression of astrocytic autophagy plasticity increases the size of β-amyloid plaques and the number of hypertrophic GFAP-positive astrocytes in a mouse model of β-amyloidosis^585^. These pathological sequelae are associated with hippocampal neuronal damage and memory loss (Fig. 14). In summary, astrocytes—unlike neurons—exhibit adaptable autophagy plasticity to increase astrocyte survival and clear β-amyloid, thereby improving cognition. Therefore, boosting astrocytic autophagy plasticity may be therapeutically relevant.

### Aerobic glycolysis—restoring astrocytic metabolic excitability

The brain mass represents around 2% of the total human body mass. However, its metabolic demands are one order of magnitude higher—the consumption of bodily glucose by the brain amounts to 20% of total body energy expenditure. This consumption increases further during cognitive tasks and memory formation and learning^586^. Under normal conditions, neurons lack energy reserves in the form of glycogen or lipid droplets. Neuronal resilience to energy-demanding states is therefore limited and depends on energy substrates supplied by neuroglia, particularly astrocytes^587–590^. Astrocytic energy metabolism is regulated by noradrenergic innervation of the brain which mainly comes from locus coeruleus^586^. Noradrenergic neurons dwelling in the locus coeruleus are sensitive to various pathological insults and become altered or malfunctional in many neurological disorders, including attention-deficit/hyperactivity disorders, sleep-associated disturbances^591^, congenital central hypoventilation syndrome, depression and anxiety, and in neurodegenerative diseases, including AD and PD^592,593^. Recent clinical research has revealed that locus coeruleus neurons are among the first to degenerate in many, if not all, neurodegenerative diseases^594^. Thus, under conditions of locus coeruleus neurodegeneration, levels of noradrenaline in the CNS are reduced, as demonstrated in an animal model of ß-amyloidosis^595^, which affects energy provision to neuronal networks. Indeed, a hypoenergetic state is regularly recorded in patients with AD^356^.

Astrocytes are regulators of CNS energy metabolism^596^. They are anatomically linked to the blood–brain barrier^597^ and mediate the uptake of substrates from the systemic circulation into the CNS. Numerous transporters are found in astrocyte plasmalemma, including those that transport glucose and fatty acids, and astrocytes store energy in the form of lipid droplets^586,598,599^ and glycogen^600,601^, which is key in generating L-lactate in the process of aerobic glycolysis^602^.

#### Adrenergic regulation of astrocyte aerobic glycolysis

The term aerobic glycolysis was introduced by Otto Heinrich Warburg^603^, who revealed that glycolysis with L-lactate production in cancer tissue occurred even in the presence of oxygen, which is generally known as the Warburg effect^604^. Aerobic glycolysis is operational in astrocytes during neurodevelopment and even in adulthood in some areas of the CNS^605^. Aerobic glycolysis is inefficient in producing ATP, but it generates biosynthetic intermediates, an advantage for developing and dynamically growing tissues^606^. Astrocytes are morphologically plastic cells, especially during learning and memory formation^416,607^, and biosynthetic aerobic glycolysis intermediates are probably key to supporting these processes^608^.

The phenomenon of aerobic glycolysis, together with its product L-lactate, is linked to the astrocyte-to-neuron lactate shuttle^609^, a concept that considers L-lactate as an energy source for neurons. Although this shuttle was presented as a cornerstone of brain energy metabolism, it has been known for a long time that glycolysis-derived pyruvate has two fates in astrocytes: conversion to L-lactate and decarboxylation in the mitochondria^610,611^. About 25% of the oxidative metabolism of the brain accounts for the pyruvate metabolism^612^. Moreover, astrocytes are metabolically unique because they preferentially produce and release citrate, a key component of mitochondrial function^613^. Furthermore, citrate is an intermediate for sterol and fatty acid biosynthesis^614,615^ and is used in astrocytes for the production of cholesterol, supporting synaptogenesis^616^, lipogenesis and lipid droplet formation in astrocytes^614,615^. Noradrenergic stimulation of astrocytes affects lipid droplet formation^598^. Pathological conditions related to neurodegeneration affect lipid droplets homeostasis, probably through impaired second messenger adrenergic signaling^617^.

#### Impaired noradrenergic regulation of astrocyte energy provision in neurodegenerative disorders

Brain activity, including cognitive functions and locomotion, increases metabolism in astrocytes and neurons. Locomotion induces energy processes differently in astrocytes and neurons. In neurons, activation of locomotion triggers a prompt increase in mitochondrial respiration, but in astrocytes, respiration develops much slower^618^. Enhanced L-lactate availability, produced by astrocytes stimulated with noradrenaline, supports learning^619^. Imaging of ^18^F 2-deoxy glucose by the MRI–PET method in patients revealed reduced D-glucose metabolism in patients suffering from neurodegeneration^356^. In part, this hypometabolic state reflects reduced levels of noradrenaline^595^ in the CNS due to degeneration of locus coeruleus. In addition to noradrenaline stimulating astrocytes to provide energy in the form of L-lactate, other mechanisms may also contribute to excitation–energy coupling. Activation of the adenosine A_2B_ receptor in astrocytes stimulated glucose metabolism and the release of L-lactate, which increases the extracellular pool of readily available energy substrates^620^.

**Fig. 15:**
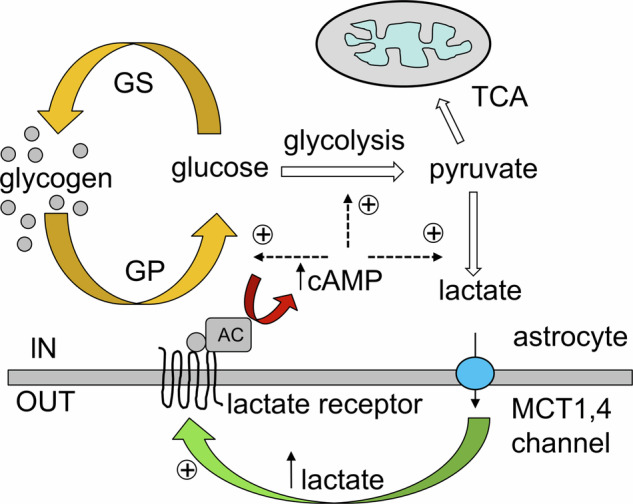
Metabolic excitability, a positive feedback mechanism enhancing L-lactate production in astrocytes. The schematic shows lactate turnover in astrocytes and its signalling function. Extracellular L-lactate controls cytosolic L-lactate synthesis in astrocytes by yet unidentified receptors coupled to adenylate cyclase activity and cytosolic cAMP increase in astrocytes. In the brain, L-lactate is formed in the cytoplasm of astrocytes (IN) and is released through monocarboxylate transporters (MCTs) 1 and 4 and/or lactate-permeable channels. Extracellularly (OUT), L-lactate can be transported to neighboring cells as an energy substrate. However, L-lactate can also act as a signaling molecule by binding to the L-lactate receptors of neighboring cells, stimulating adenylate cyclase activity (AC) and an increase in cAMP synthesis. Elevated cytosolic cAMP levels facilitate glycogen degradation by activating glycogen phosphorylase (GP) and glycolysis with L-lactate as the end product. In the absence of the L-lactate positive feedback mechanism (‘metabolic excitability’), the L-lactate tissue concentration gradient could be dissipated, reducing the availability of L-lactate as a metabolic fuel, when local energy demands, especially in the brain, are high. This model shares similarities with the ‘autocrine lactate loop’ acting (oppositely) on [cAMP]_i_ through the GPR81 receptor in adipocytes^675^. GS, glycogen synthase; TCA, tricarboxylic acid cycle. Glucose denotes phosphorylated and free glucose. Reproduced, with permission, from ref. ^624^.

When released from astrocytes, L-lactate may exit the CNS through systemic circulation^621^; what is remaining acts as a signaling molecule binding to L-lactate-sensitive receptors^622–624^. The expression of the canonical L-lactate hydroxycarboxylic acid receptor 1 (HCA1)^625^ is very low in the brain. HCA1, previously known as the orphan G protein-coupled receptor 81 (GPR81), is coupled to G_i_ protein and downregulates cAMP production. Exposure of astrocytes to extracellular L-lactate and selective HCA1 agonists, 3Cl-5OH-BA^626^ or Compound 2^627^, triggers responses similar to those mediated by β-adrenoceptors^628–630^, namely an increase in cAMP, cAMP-dependent PKA activity and L-lactate production. The latter was attenuated by adenylate cyclase pharmacological inhibition and is still present in HCA1-knockout astrocytes^624^. Thus, the effects of extracellular L-lactate and HCA1 agonists on cAMP signaling and metabolism in astrocytes are independent of HCA1 and are mediated by a yet unidentified G_s_ protein-coupled L-lactate receptor^624,631^. Upon noradrenergic stimulation L-lactate released from astrocytes can back-excite astrocytes to produce even more L-lactate during increased brain energy demand. This form of intercellular signaling was proposed to be the basis of metabolic excitability^624^ (Fig. 15), which contributes to the maintenance of the concentration gradient of the extracellular L-lactate pool favoring supplementation of neurons energy demand^624,632^. Astrocytic metabolic excitability is reduced in neurodegeneration owing to the reduced bioavailability of noradrenaline. By contrast, in a neurodevelopmental animal model of intellectual disability^631^, L-lactate sensing by astrocytes was reported to be increased, indicating the validity of the metabolic excitability model as a therapeutic target.

#### Targeting aerobic glycolysis in astrocytes

There are several strategies to contain astrocytic impairments associated with reduced levels of noradrenaline. Voluntary physical exercise improves memory by enhancing noradrenergic input^633^, an effect possibly mediated by β-adrenoceptors^634,635^. Pharmacological approaches to increase noradrenaline by using uptake inhibitors were not clinically effective^636^. Transplanting noradrenergic neurons into the CNS was shown to be effective in animal models^637^, but represents a challenging approach. Another possibility is to stimulate pathways mimicking the action of noradrenaline in producing L-lactate but independently of adrenergic receptors^638^. A possible receptor that enhances L-lactate production in astrocytes is an orphan GPR27^639^. Enhanced L-lactate production in astrocytes may engage the mechanism of metabolic excitability, maintaining the extracellular pool of L-lactate. A similar maintenance of the extracellular pool of L-lactate can be also mediated by the A_2B_ adenosine receptors^620^. While the concept is in line with improving the key pathobiological defect in neurodegeneration (locus coeruleus degeneration, reduced noradrenergic innervation and decreased L-lactate production), further developments are needed to bring the proposal to a feasible clinical outcome.

## Conclusions and further perspectives

Restoring brain function and prolonging cognitive longevity are the primary therapeutic challenges of the twenty-first century. Astrocytes—their function, protective capacity, malfunctions, and pathological changes—are fundamental to virtually every CNS disorder. Several astrocyte-specific molecules, most notably the EAAT1 and EAAT2 glutamate transporters, AQP4 water channels, Cx43 connexins, ezrin, and the intermediate filament proteins GFAP and vimentin, contribute to many of these pathologies. Targeting these molecules and developing astrocyte-specific therapeutic strategies may improve both disease-preventing and disease-modifying therapeutic outcomes in diseases of cognition.
