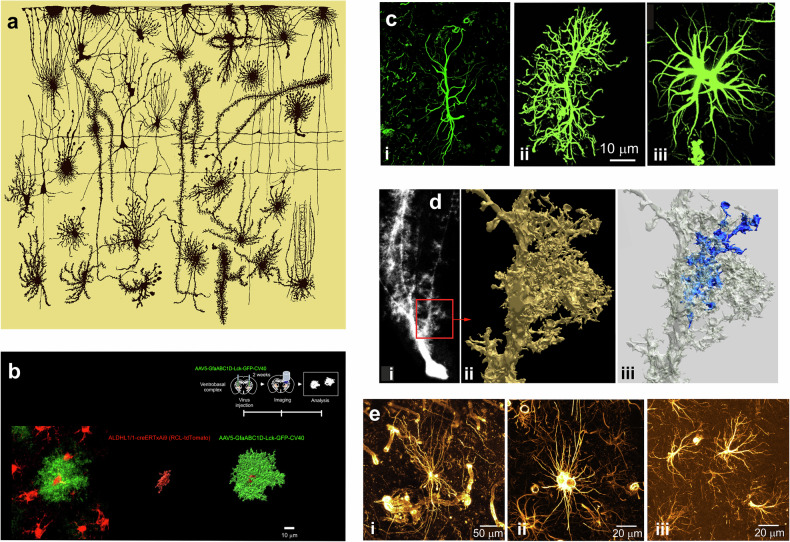# Author Correction: Curing the brain: in search for new astrocyte-specific therapies

**DOI:** 10.1038/s12276-026-01749-5

**Published:** 2026-05-11

**Authors:** Alexei Verkhratsky, C. Justin Lee, Heejung Chun, Christian Göritz, Tibor Harkany, Jae-Hun Lee, Sangkyu Lee, Maria Lindskog, Wuhyun Koh, Jan Mulder, Min-Ho Nam, Ole Petter Ottersen, Marcela Pekna, Milos Pekny, Aleksandra Pękowska, Hoon Ryu, Chang Ho Sohn, Evgenii O. Tretiakov, Verena Untiet, Tim J. Viney, Wongu Youn, Chenju Yi, Robert Zorec, Mijin Yun, Eunji Cheong, Agneta Nordberg

**Affiliations:** 1 Faculty of Biology, Medicine and Health, The University of Manchester, Manchester, UK; 2 International Joint Research Centre on Purinergic Signalling of Sichuan Province Chengdu University of Traditional Chinese Medicine, Chengdu, China; 3 Guangdong Provincial Key Laboratory of Digestive Cancer Research, The Seventh Affiliated Hospital of Sun Yat-sen University, Guangdong, China; 4 Celica, BIOMEDICAL, Ljubljana, Slovenia; 5 Department of Forensic Analytical Toxicology, School of Forensic Medicine, China Medical University, Shenyang, China; 6 Center for Memory and Glioscience, Institute for Basic Science, Daejeon, Republic of Korea; 7 Collage of Pharmacy, Yonsei-SL Institute, Yonsei University, Incheon, Republic of Korea; 8 Department of Cell and Molecular Biology, Karolinska Institutet, Stockholm, Sweden, Stockholm, Sweden; 9 Center for Neuromusculoskeletal Restorative Medicine, Hong Kong Science Park, Shatin, Hong Kong; 10 Department of Molecular Neurosciences, Center for Brain Research, Medical University of Vienna, Vienna, Austria; 11 Department of Neuroscience, Karolinska Institutet, Solna, Sweden; 12 Department of Medical Cell Biology, Uppsala University, Uppsala, Sweden; 13 Center for Brain Disorders, Brain Science Institute, Korea Institute of Science and Technology, Seoul, Republic of Korea; 14 Institute of Basic Medical Sciences, University of Oslo, Oslo, Norway; 15 Oslo New University College, Oslo, Norway; 16 Department of Clinical Neuroscience, Karolinska Institutet, Stockholm, Sweden; 17 Department of Clinical Neuroscience, Institute of Neuroscience and Physiology, Sahlgrenska Academy at the University of Gothenburg, Gothenburg, Sweden; 18 Dioscuri Centre for Chromatin Biology and Epigenomics, Nencki Institute of Experimental Biology, Polish Academy of Sciences, Warsaw, Poland; 19 Graduate School of Medical Science and Engineering, Korea Advanced Institute of Science and Technology, Daejeon, Republic of Korea; 20 Division of Astrocyte Driven Ionostasis, Center for Translational Neuromedicine, University of Copenhagen, Copenhagen, Denmark; 21 Department of Pharmacology, University of Oxford, Oxford, UK; 22 Department of Geriatrics, Seventh Affiliated Hospital of Sun Yat-sen University, Shenzhen, China; 23 Institute of Pathophysiology, Laboratory of Neuroendocrinology and Molecular Cell Physiology, University of Ljubljana, Ljubljana, Slovenia; 24 Department of Nuclear Medicine, Yonsei University College of Medicine, Seoul, Republic of Korea; 25 Department of Biotechnology, College of Life Science and Biotechnology, Yonsei University, Seoul, Republic of Korea; 26 Department of Neurobiology, Care Sciences and Society, Center for Alzheimer Research, Karolinska institutet, Stockholm, Sweden; 27 Theme Inflammation and Aging, Karolinska University Hospital, Stockholm, Sweden

**Keywords:** Astrocyte, Mechanisms of disease

Correction to: *Experimental & Molecular Medicine* 10.1038/s12276-026-01712-4, published online 24 April 2026

In Fig. 2, the figure parts are labeled as (a), (b), and (c), but should have appeared as (i), (ii), and (iii) to match the figure legend. The original article has been corrected.

incorrect figure 2
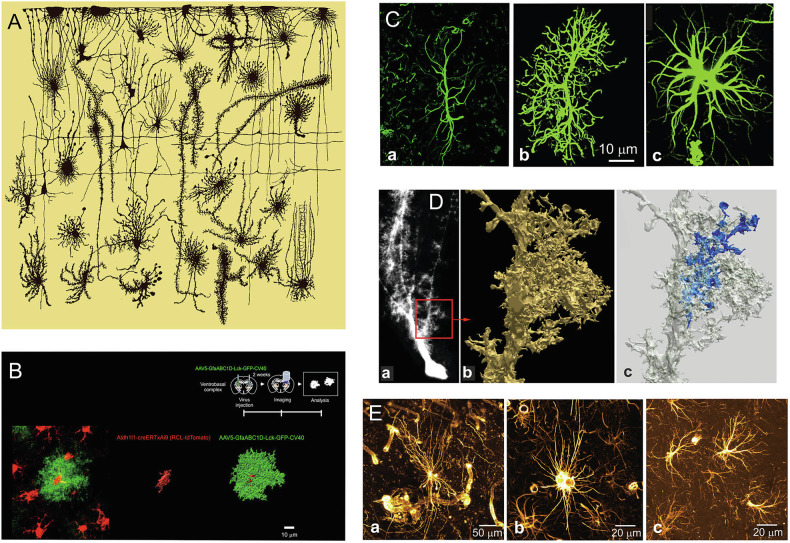


correct figure 2